# Crystal structures of seven gold(III) complexes of the form *L*Au*X*_3_ (*L* = substituted pyridine, *X* = Cl or Br)

**DOI:** 10.1107/S2056989024007266

**Published:** 2024-07-31

**Authors:** Cindy Döring, Peter G. Jones

**Affiliations:** a Institut für Anorganische und Analytische Chemie, Technische Universität Braunschweig, Hagenring 30, D-38106 Braunschweig, Germany; Vienna University of Technology, Austria

**Keywords:** crystal structure, gold, pyridine, polymorphs, twinning, halogen bonds, coinage bonds

## Abstract

The structures of seven gold(III) halide derivatives of general formula *L*Au*X*_3_ (*L* = methyl­pyridines or di­methyl­pyridines, *X* = Cl or Br) are presented: tri­chlorido­(2-methyl­pyridine)­gold(III), [AuCl_3_(C_6_H_7_N)], **1** (as two polymorphs **1a** and **1b**); tri­bromido­(2-methyl­pyridine)­gold(III), [AuBr_3_(C_6_H_7_N)], **2**; tri­bromido­(3-methyl­pyridine)­gold(III), [AuBr_3_(C_6_H_7_N)], **3**; tri­bromido­(2,4-di­meth­yl­pyridine)­gold(III), [AuBr_3_(C_7_H_9_N)], **4**; tri­chlorido­(3,5-di­methylpyridine)­gold(III), [AuCl_3_(C_7_H_9_N)], **5**; tri­bromido­(3,5-di­methyl­pyridine)­gold(III), [AuBr_3_(C_7_H_9_N)], **6**, and tri­chlorido­(2,6-di­methyl­pyridine)­gold(III), [AuCl_3_(C_7_H_9_N)], **7**. Additionally, the structure of **8**, the 1:1 adduct of **2** and **6**, [AuBr_3_(C_6_H_7_N)]·[AuBr_3_(C_7_H_9_N)], is included. All the structures crystallize solvent-free, and all have *Z*′ = 1 except for **5** and **7**, which display crystallographic twofold rotation symmetry, and **4**, which has *Z*′ = 2. **1a** and **2** are isotypic. The coordination geometry at the gold(III) atoms is, as expected, square-planar. Four of the crystals (**1a**, **1b**, **2** and **8**) were non-merohedral twins, and these structures were refined using the ‘HKLF 5’ method. The largest inter­planar angles between the pyridine ring and the coordination plane are observed for those structures with a 2-methyl substituent of the pyridine ring. The Au—N bonds are consistently longer *trans* to Br (average 2.059 Å) than *trans* to Cl (average 2.036 Å). In the crystal packing, a frequent feature is the offset-stacked and approximately rectangular dimeric moiety (Au—*X*)_2_, with anti­parallel Au—*X* bonds linked by Au⋯*X* contacts at the vacant positions axial to the coordination plane. The dimers are connected by further secondary inter­actions (Au⋯*X* or *X*⋯*X* contacts, ‘weak’ C—H⋯*X* hydrogen bonds) to form chain, double chain (‘ladder’) or layer structures, and in several cases linked again in the third dimension. Only **1b** and **7** contain no offset dimers; these structures instead involve C—H⋯Cl hydrogen bonds combined with Cl⋯Cl contacts (**1b**) or Cl⋯π contacts (**7**). The packing patterns of seven further complexes *L*Au*X*_3_ involving simple pyridines (taken from the Cambridge Structural Database) are compared with those of **1**–**8**.

## Abstract 2

The structures of seven complexes of general formula *L*Au*X*_3_ (*L* = methyl­pyridines or di­methyl­pyridines, *X* = Cl or Br) are presented. In the crystal packing, a frequent feature is the offset-stacked and approximately rectangular dimeric moiety (Au—*X*)_2_, linked by Au⋯*X* contacts.

## 1. Chemical context

In the previous part (Döring & Jones, 2024*a* ▸) of our series of publications ‘Gold complexes with amine ligands’, we reported the structures of four gold(I) halide complexes involving methyl­pyridine (picoline) and di­methyl­pyridine (lutidine) ligands. That publication presents much introductory material that we do not repeat here. For convenience, we have inter­preted the term ‘amine’ liberally to include aza-aromatics.

In this publication we describe the structures of seven gold(III) halide derivatives of general formula *L*Au*X*_3_ (*L* = methyl­pyridines or di­methyl­pyridines, *X* = Cl or Br). These are: tri­chlorido­(2-methyl­pyridine)­gold(III) **1** (as two polymorphs **1a** and **1b**); tri­bromido­(2-methyl­pyridine)­gold(III) **2**; tri­bromido­(3-methyl­pyridine)­gold(III) **3**; tri­bromido­(2,4-di­methyl­pyridine)­gold(III) **4**; tri­chlorido­(3,5-di­methyl­pyridine)gold(III) **5**; tri­bromido­(3,5-di­methyl­pyridine)­gold(III) **6** and tri­chlorido­(2,6-di­methyl­pyridine)­gold(III) **7**. Additionally, we present the structure of **8**, the 1:1 adduct of **2** and **6**.
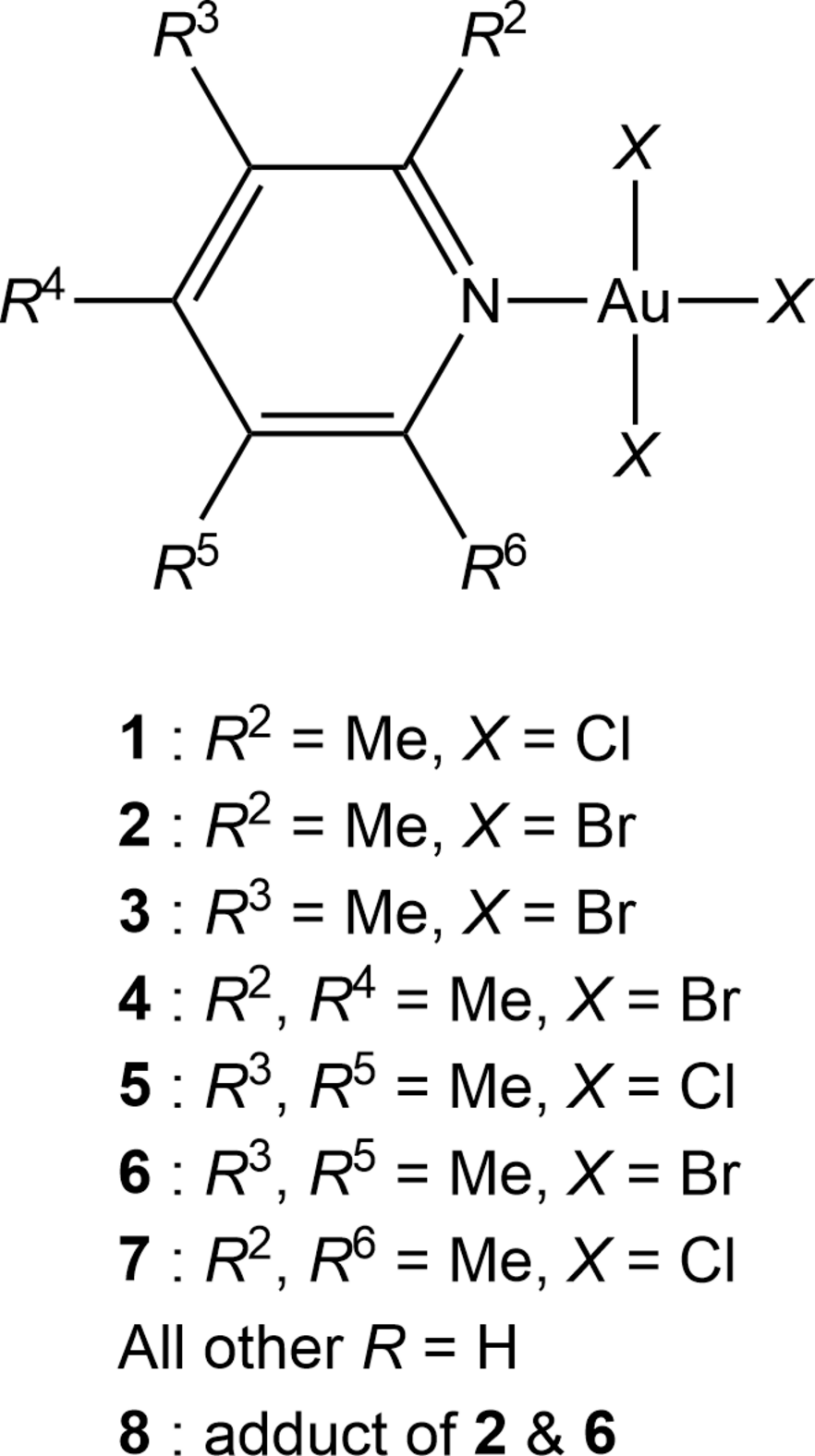


*Note added during revision*: A referee commented that **8** might be referred to as a co-crystal rather than an adduct. This is certainly a reasonable suggestion in view of the IUCr definition of a co-crystal (https://dictionary.iucr.org/Co-crystal): ‘Solid consisting of a crystalline single-phase material composed of two or more different mol­ecular and/or ionic compounds, generally in a stoichiometric ratio, which are neither solvates nor simple salts.’ The problem in our view is that a solid is not necessarily the same as a crystal. We would therefore prefer to say that we studied a co-crystal of the adduct **8**. The IUCr dictionary is an extremely useful document, but it is often difficult to provide watertight definitions of any given concept. For example, Bombicz (2024 ▸) recently offered reasoned criticism of the IUCr definition of ‘isostructural/isotypic’, and we supported her views in our previous paper (Döring & Jones, 2024*a* ▸).

The structure of the parent compound (py)AuCl_3_, Cambridge Structural Database (CSD; Groom *et al.*, 2016 ▸) refcode PYAUCL10, was presented by Adams & Strähle (1982 ▸) (‘py’ = ‘pyridine’ throughout this paper). Two other compounds with the composition (py)Au*X*_3_ were in fact adducts of the type {[(py)_2_Au*X*_2_]^+^[Au*X*_4_]^−^·[(py)Au*X*_3_]} [*X* = Cl, KILFIV; Bourosh *et al.* (2007 ▸); *X* = Br, WOQMEU; Peters *et al.*, 2000 ▸)]. The only related alkyl­pyridine structure is that of (4-Et-py)AuCl_3_ (ESITIM; Hobbollahi *et al.*, 2019 ▸). Other derivatives involving ‘simple’ substituted pyridines as ligands are the isotypic pair (4-CN-py)Au*X*_3_ (*X* = Cl, WIRGAH or Br, WIRFUA; Mohammad-Natij *et al.*, 2013 ▸) and several complexes (3-*X*^1^-py)Au*X*^2^_3_ (*X*^1^ = halogen, *X*^2^ = Cl or Br) (Pizzi *et al.*, 2022 ▸). The structures of this latter series and of (4-Et-py)AuCl_3_ are discussed in the section *Database survey*.

## 2. Structural commentary

**Figure 1:**
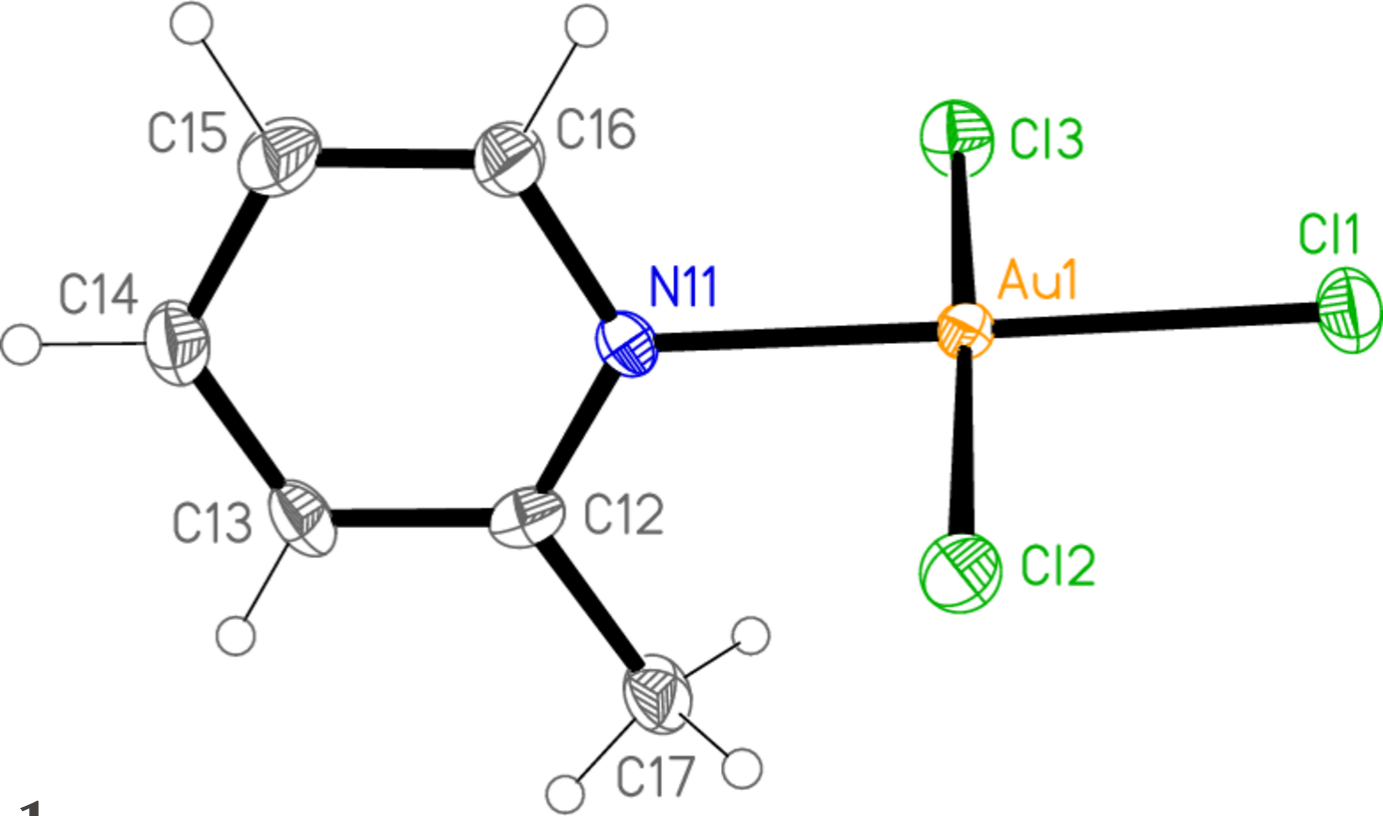
The mol­ecular structure of compound **1** (polymorph **1a**) in the crystal.

All the structures crystallize solvent-free; *Z*′ values are 0.5 for **5** and **7**, which display crystallographic twofold symmetry (with atoms N11, C14, Au1 and Cl1 on the twofold rotation axes 0.5, *y*, 0.75 and 0.5, *y*, 0.25, respectively), 2 for **4** and 1 for all other structures. Structures **1a** and **2** are isotypic, but **5** and **6**, which also differ only in the halogen, are not. Figs. 1 ▸–9 ▸ ▸ ▸ ▸ ▸ ▸ ▸ ▸ show the mol­ecules of these compounds in the crystal, with ellipsoids drawn at the 50% probability level. Selected bond lengths and angles are given in Tables 1 ▸–9 ▸ ▸ ▸ ▸ ▸ ▸ ▸ ▸. The mol­ecules are numbered such that atoms *X*1 (and *X*4, where two independent mol­ecules are present) are *trans* to the pyridinic nitro­gen atoms. The numbering of *X*2/*X*3, *cis* to the pyridinic nitro­gen, is chosen to make *X*2—Au1—N11—C12 the smallest absolute torsion angle (with appropriately altered numbering for structures with two residues). This does not apply to **5** and **7**, for which the *cis* sites are symmetry-related. The ring numbering of **6** (C12 to C16), otherwise ambiguous, is assigned by the same criterion.

The pyridine rings are as expected planar, with r.m.s. deviations of the six ring atoms between 0.002 and 0.01 Å. The coordination geometry at the central gold(III) atoms is, also as expected, square-planar; the r.m.s. deviations from the plane of Au, N and the three *X* atoms range from zero for **5** and **7** (by symmetry) to 0.058 Å for **3**, whereby the donor atoms alternate above and below the plane by *ca* 0.06 Å; a similar alternation is observed for the di­methyl­pyridine component of the adduct **8**, whereas the same mol­ecule alone (structure **6**) has a much lower r.m.s. deviation of 0.012 Å. The angles between these two planes are 78.4 (1)° for **1a**, 84.7 (2)° for **1b**, 78.7 (2)° for **2**, 57.2 (1)° for **3**, 84.5 (1)° and 74.8 (1)° for the two mol­ecules of **4**, 51.0 (1)° for **5**, 56.0 (1)° for **6**, 83.4 (1)° for **7** and 58.2 (2) and 84.3 (2)° for the two components of the adduct **8**, corresponding to compounds **6** and **2**. The largest angles are thus observed for those structures with a 2-methyl substituent of the pyridine ring, and presumably serve to reduce steric stress between these substituents and the *X* atoms *cis* to the nitro­gen donor atom at Au. The gold atoms lie up to 0.15 (1) Å (for **1b**) out of the pyridine plane, but lie exactly in this plane (by symmetry) for **5** and **7**.

Bond lengths and angles may be regarded as normal. The Au—N bonds are consistently longer *trans* to Br [average (av.) of seven bonds: 2.059 Å] than *trans* to Cl (av. of four bonds: 2.036 Å), reflecting a greater *trans* influence of the bromido ligand compared to chlorido. There is no clear difference between Au—Cl bond lengths *trans* to N compared with those *cis* to N, whereas Au—Br bonds *trans* to N (av. of seven bonds: 2.395 Å) are significantly shorter than those *cis* to N (av. of fourteen bonds: 2.421 Å). The bond angles at Au are close to the ideal 90°/180°; the angles with the largest deviations for the former are 88.25 (10)° for N1—Au1—Br2 of **3** and 91.17 (5)° for Cl3—Au1—Cl1 of **1b**, and for the latter 176.590 (17)° for Br6—Au2—Br5 of **4**. The C—N—C angles of the py ligands are all close to 120° (av. of eleven angles: 120.8°).

**Figure 10:**
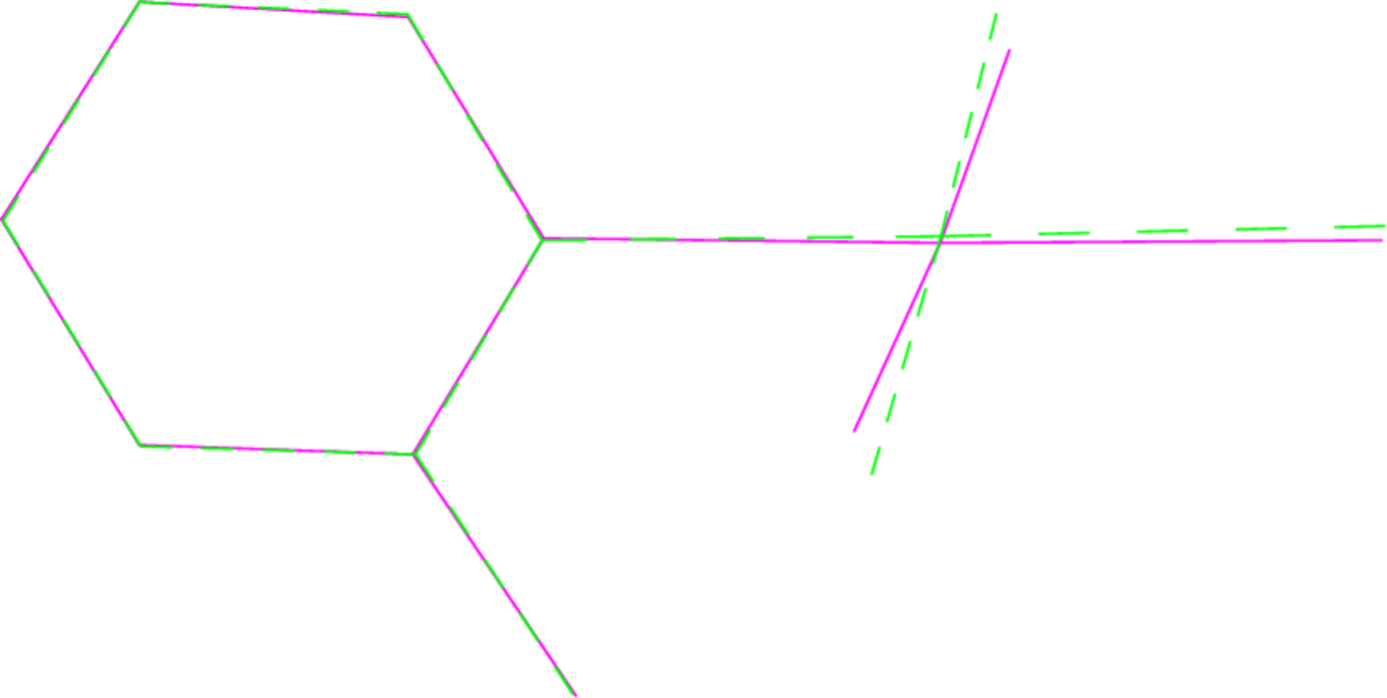
A least-squares fit of the pyridinic ligands of **1a** and **1b** (excluding H atoms). **1a** is the dotted mol­ecule.

**Figure 11:**
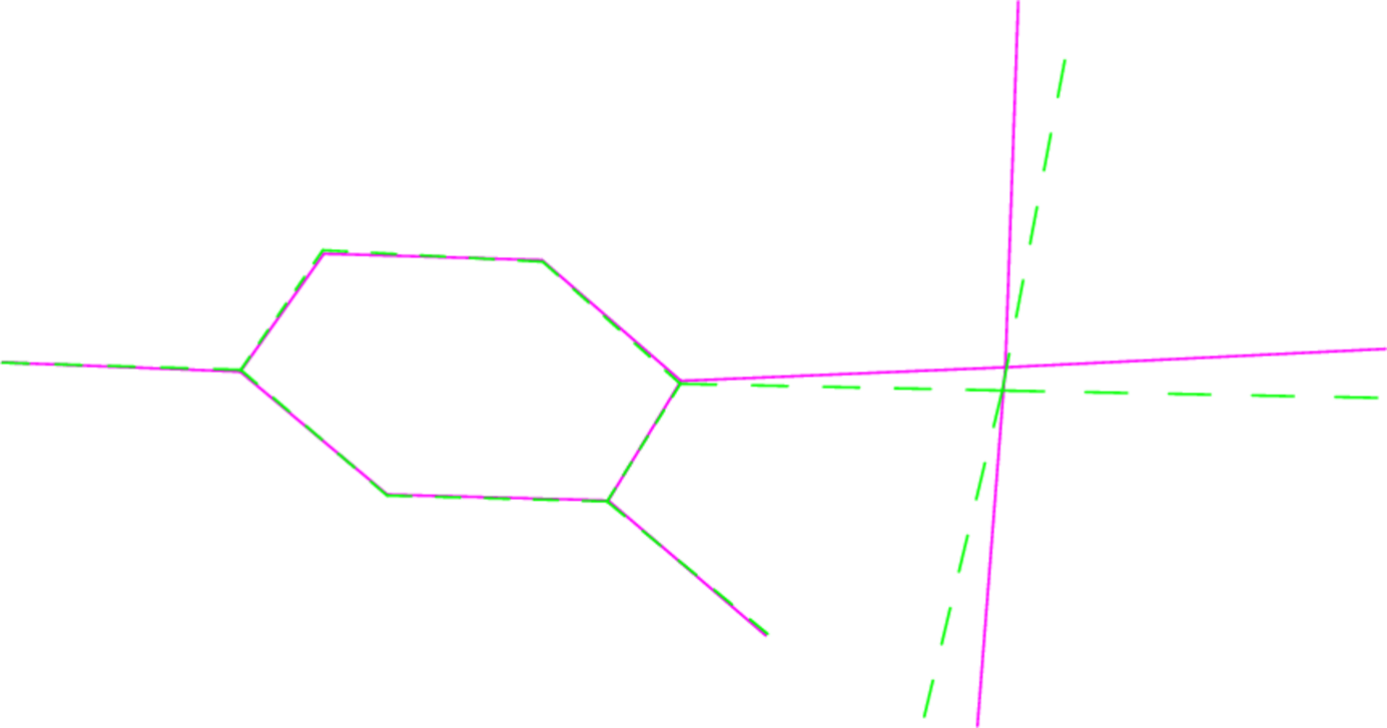
A least-squares fit of the pyridinic ligands of both mol­ecules of **4** (excluding H atoms). Mol­ecule 1 (centred on Au1) is dotted.

**Figure 12:**
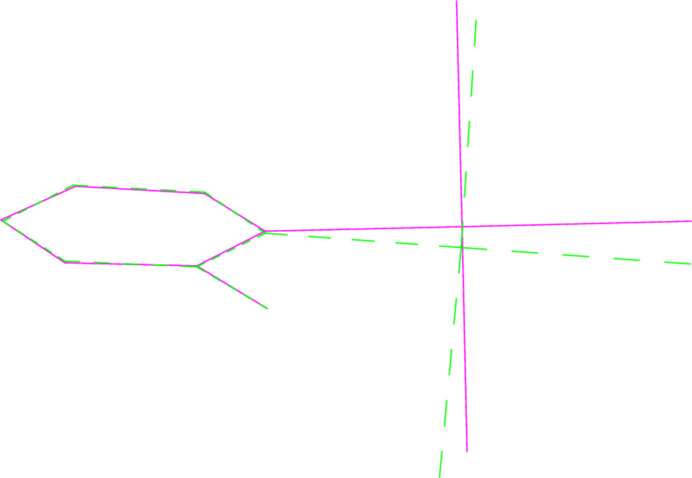
A least-squares fit of the pyridinic ligands of **1b** and its counterpart in the adduct **8** (excluding H atoms). **1b** is the dotted mol­ecule.

**Figure 13:**
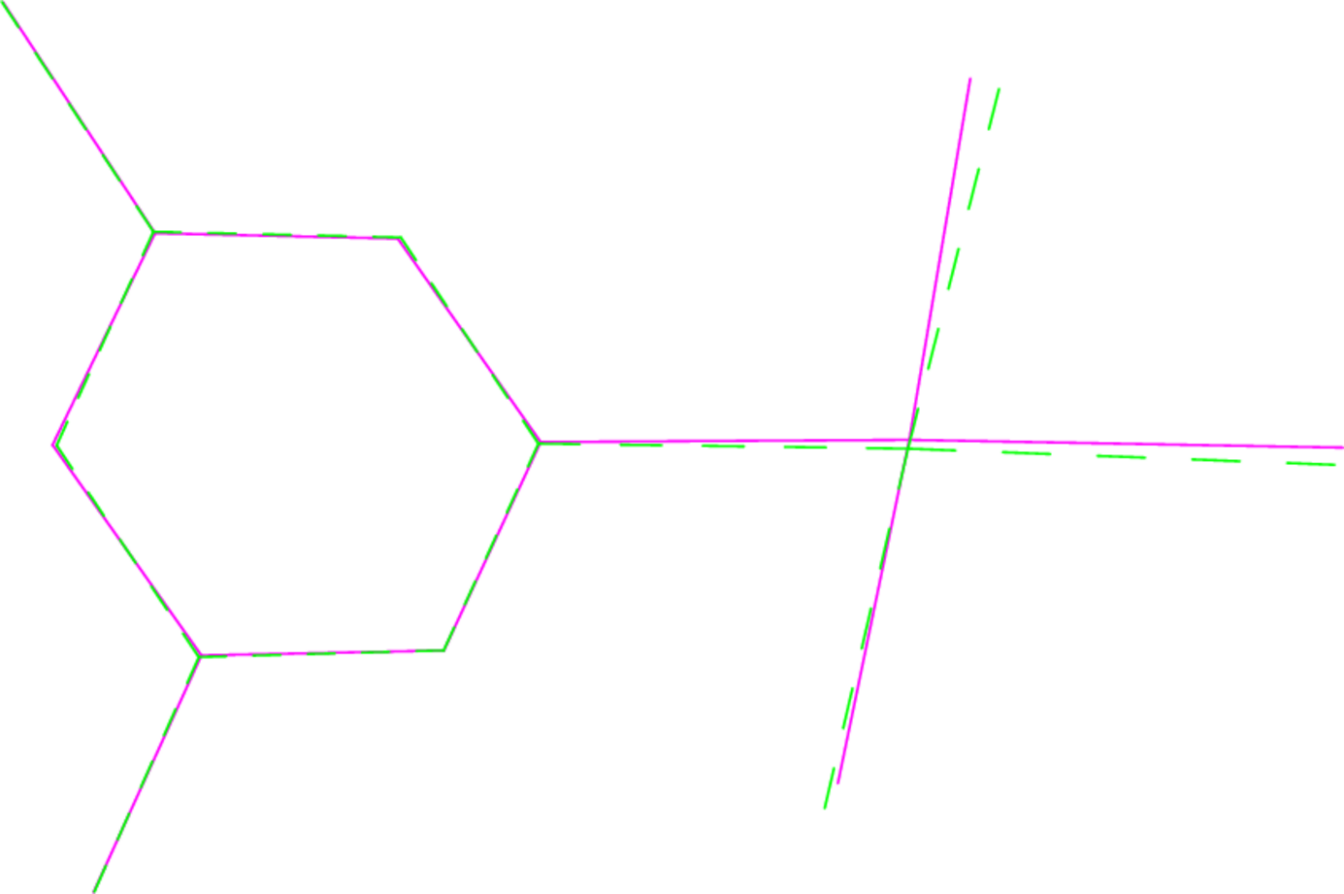
A least-squares fit of the pyridinic ligands of **6** and its counterpart in the adduct **8** (excluding H atoms). **6** is the dotted mol­ecule.

A least-squares fit of the polymorphs **1a** and **1b** gave an r.m.s. deviation of 0.08 Å; a similar fit of the two independent mol­ecules of **4** (one inverted) gave a deviation of 0.16 Å. Fits of mol­ecules **2** and **6** (the latter inverted) to the same mol­ecules of the adduct **8** gave r.m.s. deviations of 0.091 and 0.061 Å, respectively. More informative figures are however obtained by fitting only the pyridine ligands, which are closely similar; the differences associated with the Au*X*_3_ moieties are then shown more clearly. For **1a**/**1b**, the atoms Cl2 and Cl3 differ in position by 0.26 and 0.20 Å respectively (Fig. 10 ▸). For **4**, the gold atoms lie on opposite sides of the pyridine plane, and this, coupled with the 10° difference in the inter­planar angle, leads to significant differences in the positions of the bromine atoms (0.39, 0.50, 0.51 Å, respectively for Br1–3; Fig. 11 ▸). A similar effect, although the inter­planar angles are almost equal, is seen for the fit of **1b** with its counterpart in the adduct **8** (deviations 0.50, 0.43, 0.40 Å; Fig. 12 ▸), whereas the largest difference for the fit of **6** with its counterpart in **8** is for Br1 (0.22 Å; Fig. 13 ▸).

## 3. Supra­molecular features

Hydrogen bonds of the type C—H⋯*X* for all structures are given in Tables 10 ▸–18 ▸ ▸ ▸ ▸ ▸ ▸ ▸ ▸. These include several borderline cases that are not discussed explicitly. For all packing diagrams, the labelling indicates the asymmetric unit, and hydrogen atoms not involved in secondary contacts are omitted for clarity. The choice of ‘important’ inter­actions and their hierarchy is necessarily subjective, at least to some extent; diagrams with a small number of heavy-atom contacts are easier to inter­pret than those involving a larger number of hydrogen bonds, and this is especially true for H⋯Br contacts, which are probably weaker than H⋯Cl. Primes (′,′′) indicate symmetry-equivalent atoms; operators are not given in full each time. A summary of the packing features is given in Table 19 ▸.

**Figure 14:**
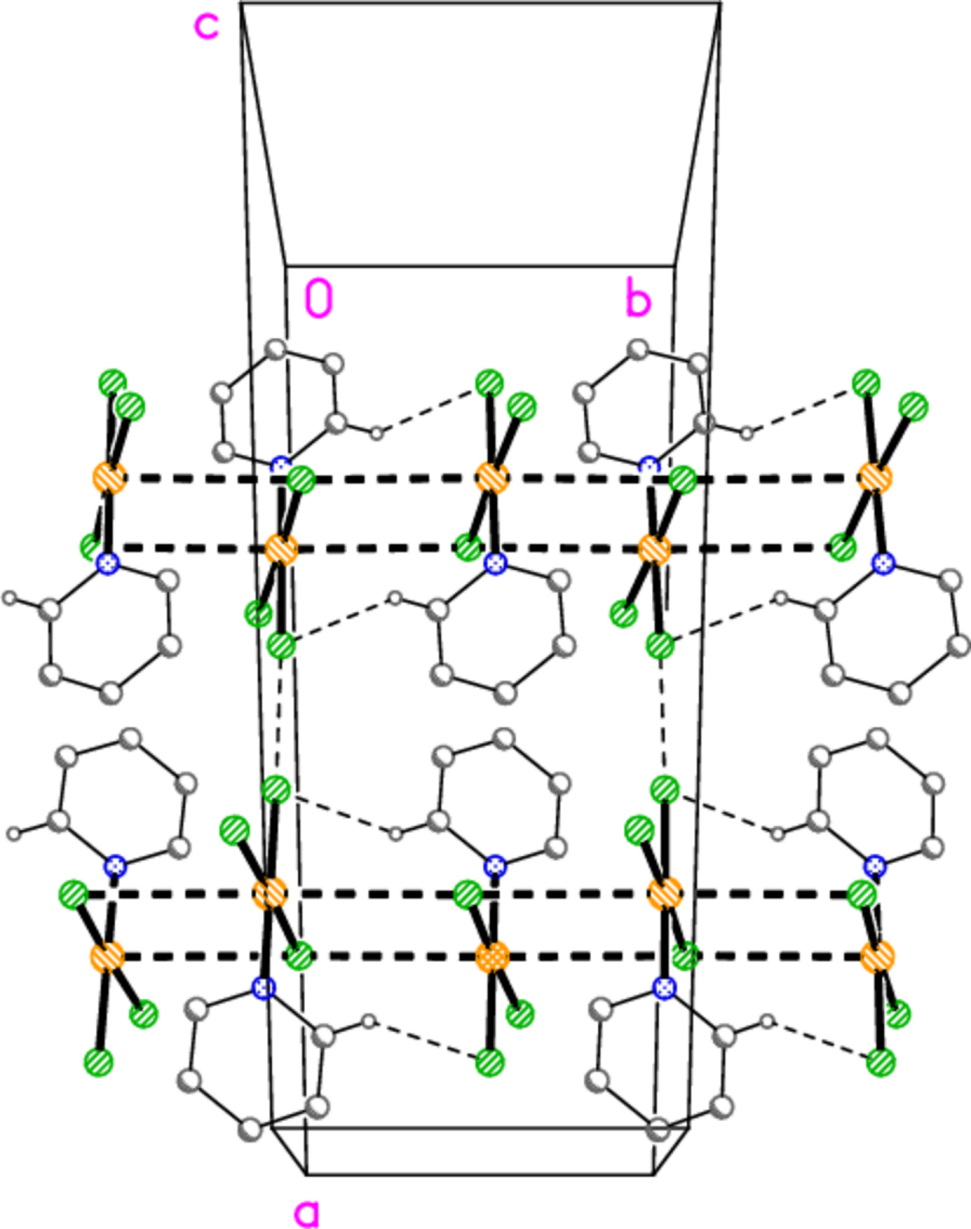
The packing of (py)AuCl_3_ (Adams & Strähle, 1982 ▸), showing two adjacent ladder-like double chains parallel to the *b* axis at (*x*, *z*) = (0.25, 0) and (0.75, 0.5). The view direction is approximately parallel to the *c* axis (but rotated slightly to reduce overlap). Thick dashed lines indicate Au⋯Cl contacts; thin dashed lines indicate ‘weak’ H⋯Cl hydrogen bonds or short Cl⋯Cl contacts. Atomic coordinates were taken from the database (refcode PYAUCL10); hydrogen-atom positions were calculated using the HADD option of XP (Bruker, 1998 ▸). Colour codes for this Figure and for Figs. 29 ▸–34 ▸ ▸ ▸ ▸ ▸ are the same as for those of **1**–**8** (C and H black, N dark blue, Au yellow, Cl green, Br brick-red), but we do not number the atoms in these Figures because the database numbering is not consistent *e.g.* for *cis* and *trans* halogen atoms.

Before discussing the packing of **1**–**8** in detail, it is useful to look back on the packing of (py)AuCl_3_ (Adams & Strähle, 1982 ▸; space group *C*2/*c*, *Z* = 8), to see what types of secondary inter­action can arise. Short non-bonded contacts were observed between the gold atom and two chlorine atoms, positioned axially to the main coordination plane in such a way as to complete a highly stretched octa­hedron at the gold atom (Au⋯Cl 3.636 and 3.648 Å, Cl⋯Au⋯Cl 173.0°; operators 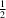 − *x*, −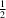 + *y*, 1 − *z* and 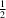 − *x*, 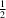 + *y*, 1 − *z* ). This leads to ladder-like double chains of residues (Fig. 14 ▸), parallel to the *b* axis, in which the mol­ecules display offset stacking of the Au*X*_3_ groups; one Au—Cl bond of each mol­ecule (the rungs of the ladder) shares two Au⋯Cl contacts with anti­parallel Cl—Au bonds of each neighbouring mol­ecule (the side rails of the ladder). The (Au—*X*)_2_ quadrilaterals, approximately rectangular and with side lengths corresponding to the Au—Cl bond length and the Au⋯Cl contact distance, are a recurring feature in the structures discussed here. Offset stacking of this type is a common feature in Au*X*_3_ complexes, and we have observed it *e.g.* in four modifications of (tetra­hydro­thio­phene)AuCl_3_ (Upmann *et al.*, 2017 ▸). In general, any suitable donor atoms can occupy these two contact sites. It might be argued that such contacts are merely connected with the steric ease of approach to the two sides of the coordination plane; this has also been argued for short contacts to the linearly coordinated gold atom of gold(I) complexes, although H⋯Au hydrogen bonding in such systems is reasonably well established (Schmidbaur, 2019 ▸; Schmidbaur *et al.*, 2014 ▸). However, recent studies and calculations (Daolio *et al.*, 2021 ▸; Pizzi *et al.*, 2022 ▸) have indicated that there is a π-hole at the gold atom, and that there is thus a definite attractive inter­action, a ‘coinage bond’, between the gold atom and the additional donor(s) (see below).

At the time of publication of the (py)AuCl_3_ structure, more than 40 years ago (the data were probably recorded in the late 1970s), the main inter­est in crystal structure determinations generally centred on the mol­ecule being studied, whereas inter­molecular contacts were often neglected. The analysis of the Au⋯Cl contacts in (py)AuCl_3_ constituted a welcome exception. However, the structure contains other secondary contacts that were not mentioned, probably because at the time such contacts were not regarded as significant. First, there is a short Cl⋯Cl contact of 3.462 Å connecting the ‘ladders’. Such formally non-bonding contacts between halogen atoms have been the subject of considerable inter­est for some time and are usually termed ‘halogen bonds’. For C—*X*⋯*X*—C systems, they are considered to involve a small region of positive charge in the extension of the C—*X* bond vectors beyond the atom *X*, often leading to one C—*X*⋯*X* angle of *ca* 90° and one of *ca* 180° (see *e.g.* Metrangolo *et al.*, 2008 ▸, or Cavallo *et al.*, 2016 ▸, for review articles); they are quite common for Au—*X* systems, but we are not aware of any systematic and/or theoretical study of *X*⋯*X* contacts in these systems. We have drawn attention to *X*⋯*X* contacts in various tetra­halogenidoaurate(III) salts (*e.g.* Döring & Jones, 2016 ▸) and in *L*AuX_3_ complexes (*e.g.* Döring & Jones, 2024*b* ▸), and recently presented a short database survey of the latter (Döring & Jones, 2023 ▸). Secondly, there are short contacts of the type C—H⋯Cl that are now regarded as hydrogen bonds and are, somewhat misleadingly, often termed ‘weak’ hydrogen bonds. These were not mentioned in the 1982 publication, and indeed no hydrogen atoms were included in the refinement, which was not unusual at the time for heavy-atom structures. We used the program *XP* (Bruker, 1998 ▸) to calculate the hydrogen-atom positions, and established that there are three short H⋯Cl contacts, one as short as 2.79 Å; this connects neighbouring mol­ecules in the ladders. Two further such contacts (2.85 and 2.86 Å) connect the ladders; these are omitted from Fig. 14 ▸ for clarity.

**Figure 15:**
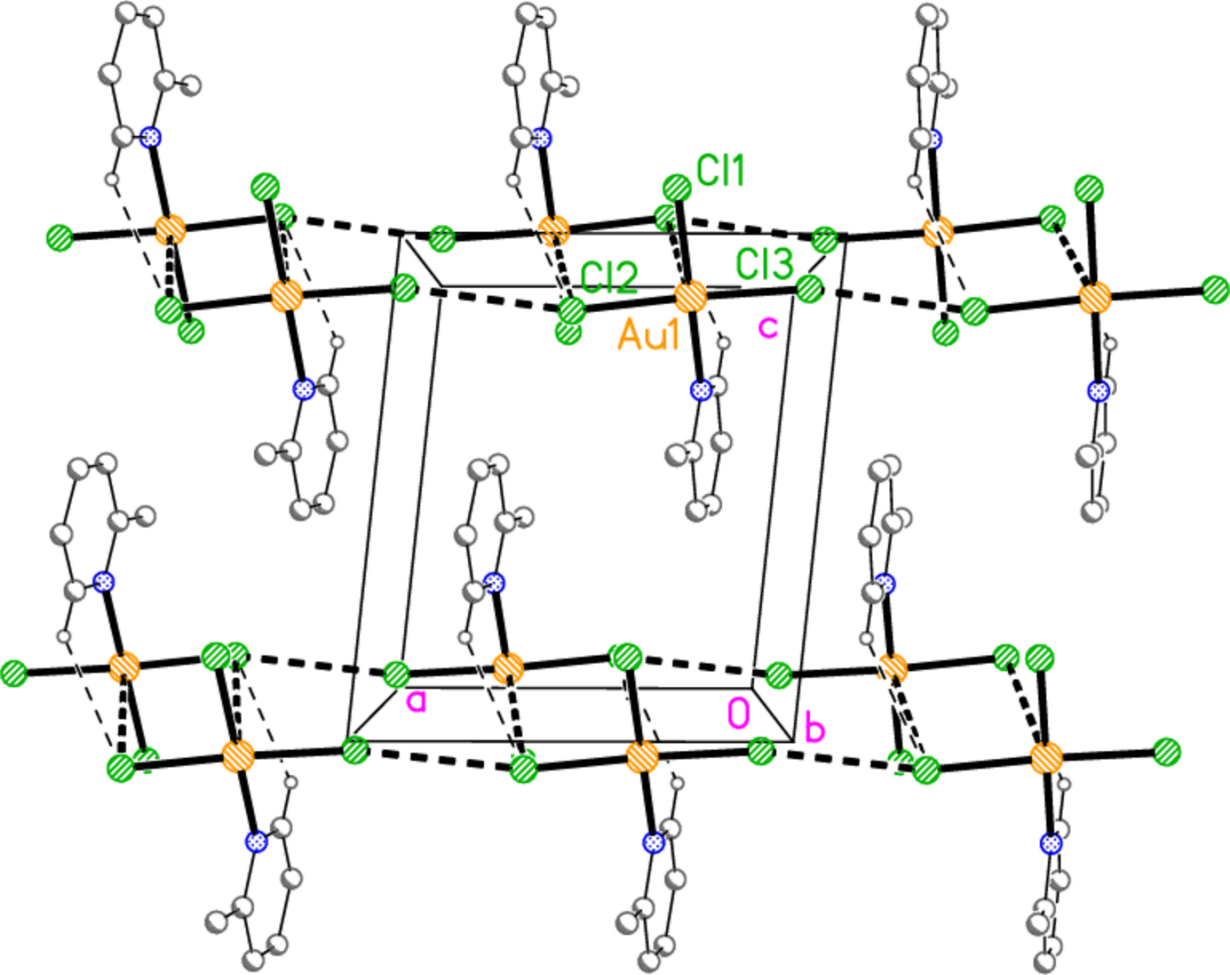
Packing diagram of compound **1**, polymorph **1a**, viewed parallel to the *b* axis in the region *y* ≃ 0.5. Dashed bonds indicate Au⋯Cl or Cl⋯Cl contacts (thick) or H⋯Cl hydrogen bonds (thin).

**Figure 16:**
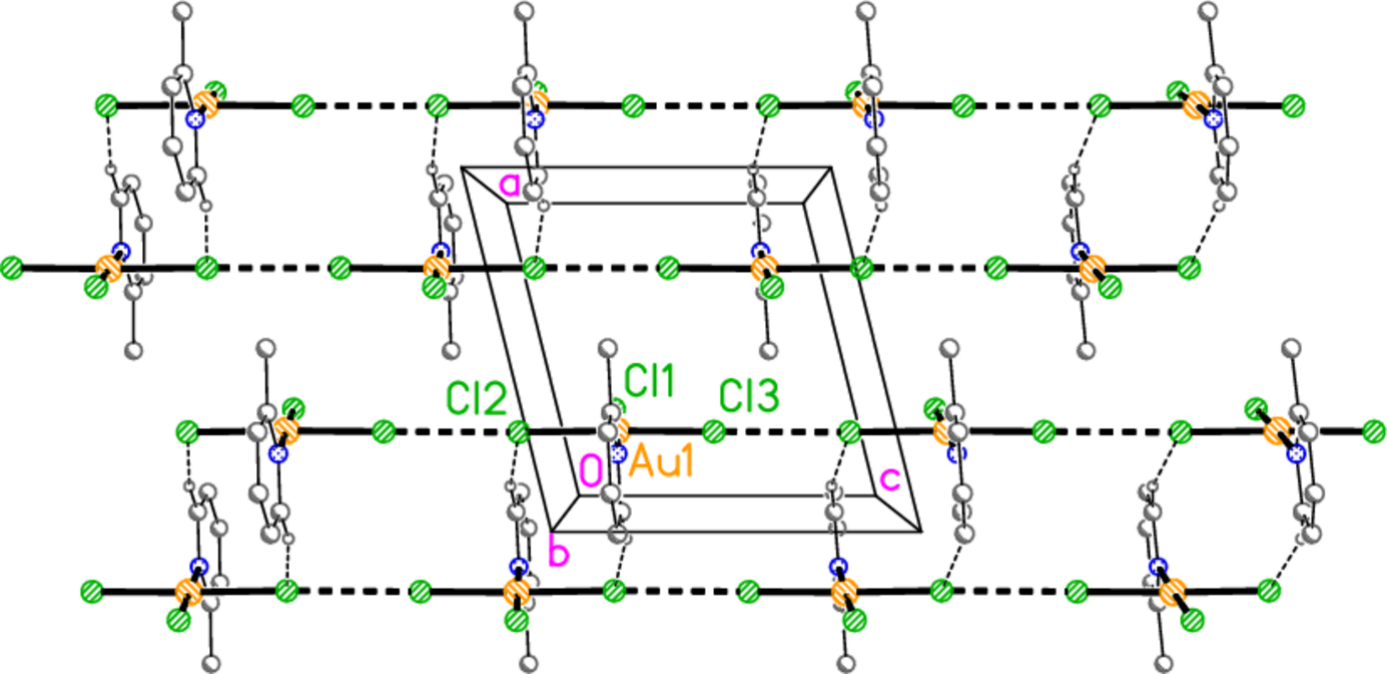
Packing diagram of compound **1**, polymorph **1b**, viewed parallel to the *b* axis in the region *y* ≃ 0.5. Dashed bonds indicate Cl⋯Cl contacts (thick) or H⋯Cl hydrogen bonds (thin).

The packing diagram of compound **1a** is shown in Fig. 15 ▸. The mol­ecules are linked to form inversion-symmetric dimers (operator 1 − *x*, 1 − *y*, 2 − *z*) in an offset packing pattern, with Au1⋯Cl2′ = 3.441 (2) Å; reinforcement is provided by the shortest hydrogen bond H16⋯Cl2′. The dimers are in turn linked by a short contact Cl2⋯Cl3(1 + *x*, *y*, *z*) = 3.239 (2) Å to form double chains parallel to the *a* axis. The angles Au1—Cl2⋯Cl3′ and Au1—Cl3⋯Cl2′ are 161.09 (6) and 162.30 (7)°, respectively. There are no other Cl⋯Cl contacts < 3.8 Å. In the packing of compound **2** (isotypic to **1a**) the corresponding dimensions are Au1⋯Br2′ = 3.5654 (8), Br2⋯Br3 = 3.3840 (9) Å, Au1—Br2⋯Br3′ = 164.38 (3), Au1—Br3⋯Br2′ = 159.34 (3)°. The second polymorph **1b** has no Au⋯Cl contact shorter than 3.833 (2) Å for Au1⋯Cl2(−*x*, 1 − *y*, −*z*), but has an even shorter Cl⋯Cl contact: Cl2⋯Cl3(*x*, *y*, −1 + *z*) = 3.164 (2) Å. This combines with the shortest H⋯Cl hydrogen bond, again H16⋯Cl2, to form double chains of mol­ecules parallel to the *a* axis (Fig. 16 ▸), with Au1—Cl2⋯Cl3′ = 172.65 (7)° and Au1—Cl3⋯Cl2′ = 174.55 (8)°. The double chains are linked in the **b** direction by the hydrogen bond H13⋯Cl1. It is notable throughout this series of structures that the *cis* (to the pyridine ligands) halogen atoms *X*2 and *X*3 (or *X*5 and *X*6) tend to be involved in the main packing features, whereas the *trans* halogen atom *X*1 (or *X*4) often provides the additional linkages.

**Figure 17:**
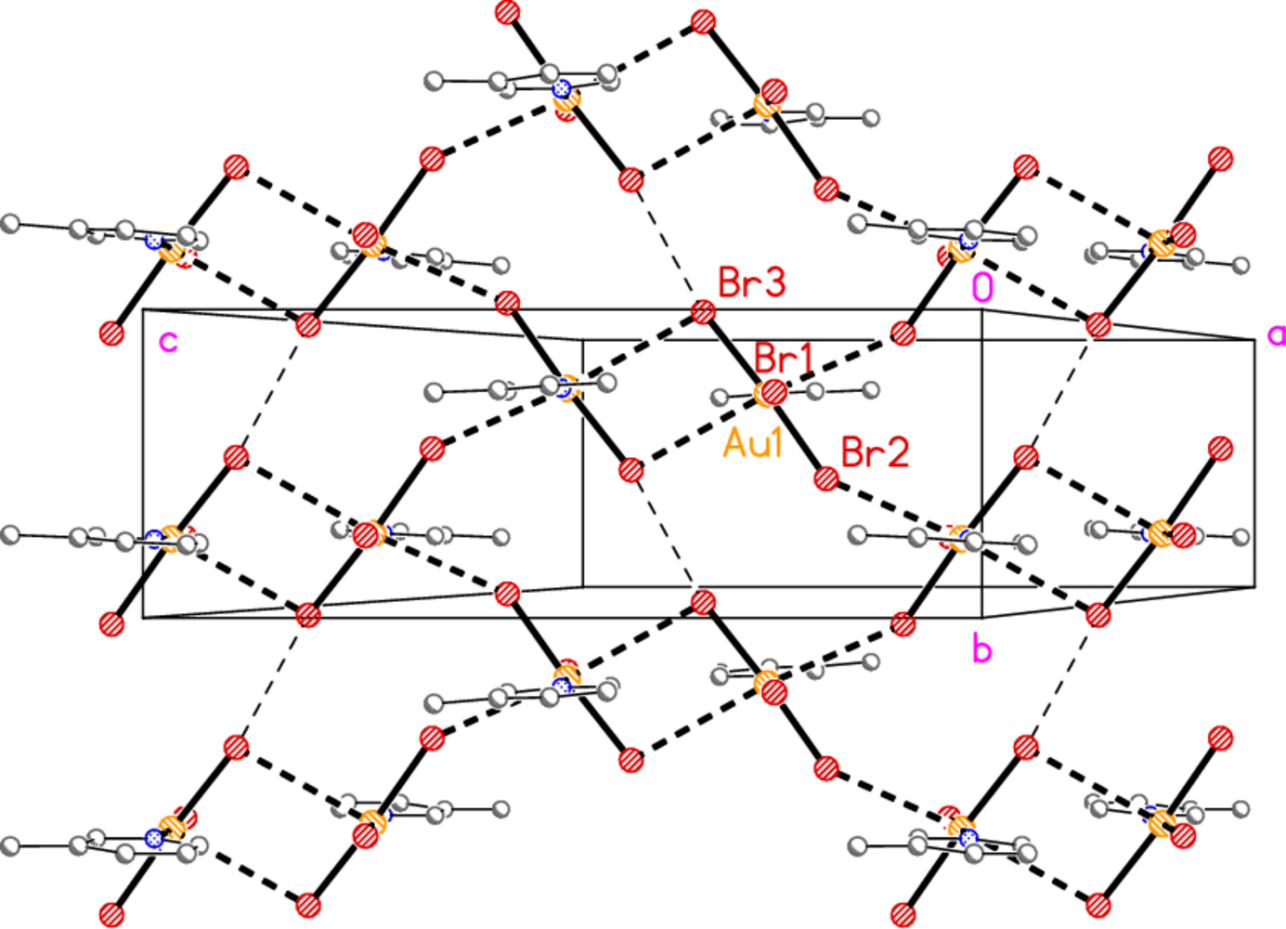
Packing diagram of compound **3**, viewed perpendicular to the *bc* plane in the region *x* ≃ 0.25. Dashed bonds indicate Au⋯Br contacts (thick) or Br3⋯Br3 contacts (thin).

**Figure 18:**
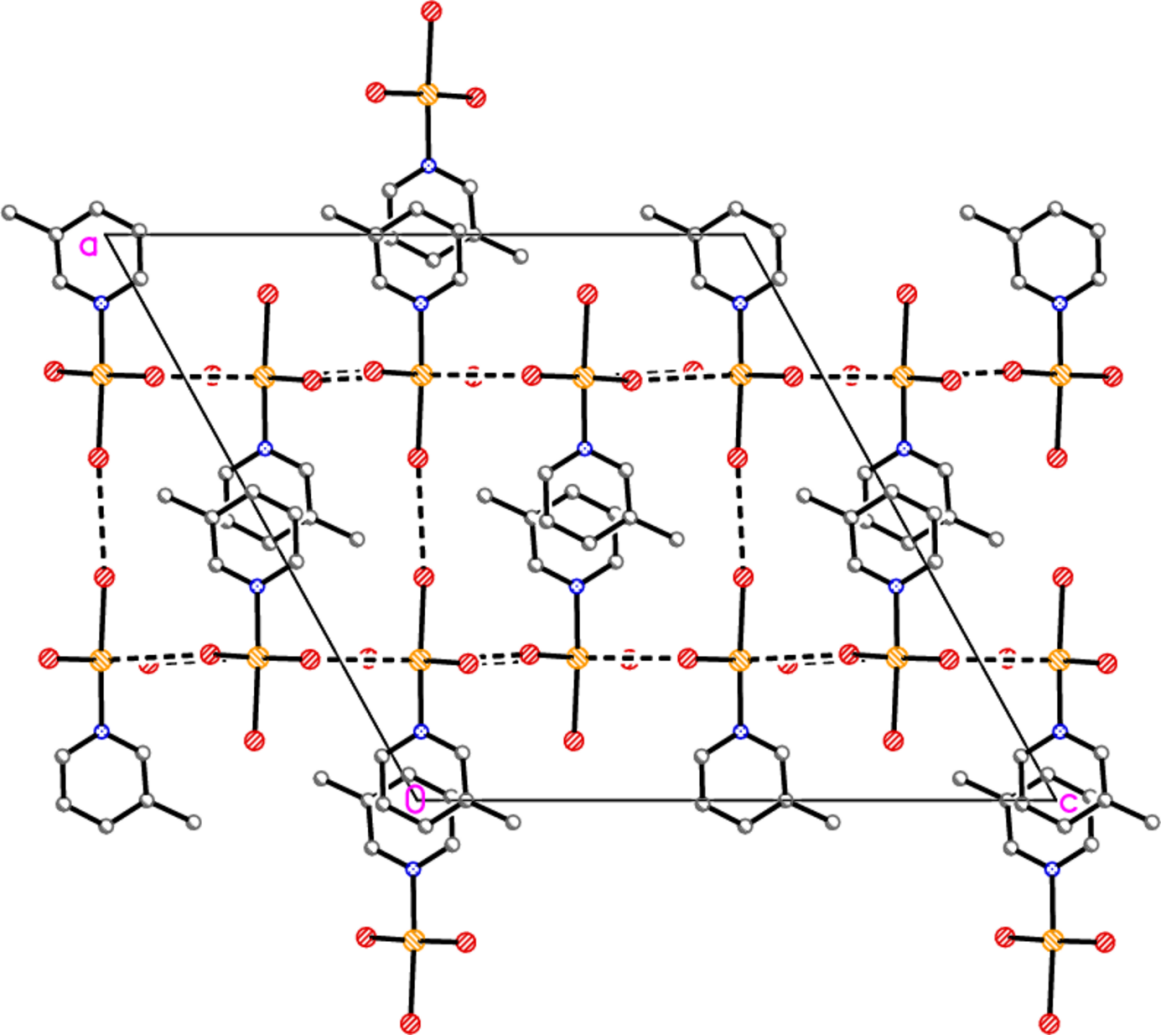
Packing diagram of compound **3**, projected parallel to the *b* axis, showing the linking of the layers of Fig. 17 ▸ by the Br1⋯Br1 contacts (thick dashed lines, vertical).

The packing of compound **3** consists of layers parallel to the *bc* plane (Fig. 17 ▸) at *x* ≃ 0.25 and 0.75, in which the two contacts Au1⋯Br2(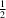 − *x*, −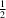 + *y*, 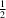 − *z*) = 3.4688 (5) Å and Au1⋯Br3(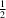 − *x*, 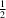 − *y*, 1 − *z*) = 3.6535 (5) Å complete a stretched octa­hedron at the gold atom [angles Br2′⋯Au1⋯Br3′ = 173.91 (1)°, Au1—Br2⋯Au1′ = 148.42 (2)°]. The Au1⋯Br3 contacts generate an offset-stacked dimer, but this stacking is not further extended. The contact Br3⋯Br3(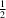 − *x*, −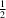 − *y*, 1 − *z*) = 3.5256 (9) Å completes the layer, with Au1—Br3⋯Br3′ = 170.31 (3)°. The two shortest H⋯Br contacts also lie within this layer, but are omitted from Fig. 17 ▸ for clarity. The layers are linked by the Br1⋯Br1(−*x*, *y*, 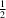 + *z*) contact of 3.4531 (9) Å, with Au1—Br1⋯Br1′ = 174.62 (3) Å, and by stacking of the pyridine rings [inter­centroid distances 3.657 (2) and 3.619 (2) Å, slippage 1.28 and 1.38 Å, operators 1 − *x*, −*y*, 1 − *z* and 1 − *x*, 1 − *y*, 1 − *z* respectively] (Fig. 18 ▸). No other structure presented here has an inter­centroid distance between the rings < 3.70 Å.

**Figure 19:**
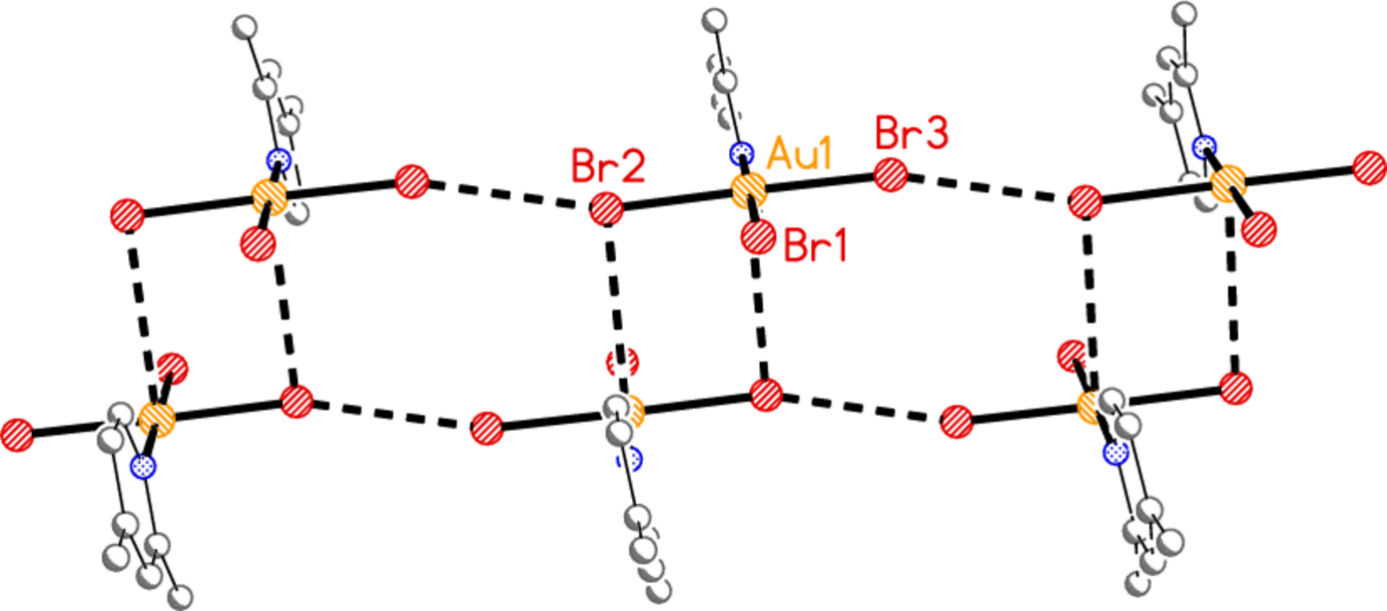
A double chain of mol­ecules 1 for compound **4**, with view direction parallel to the *b* axis. Dashed lines indicate Au⋯Br and Br⋯Br contacts.

**Figure 20:**
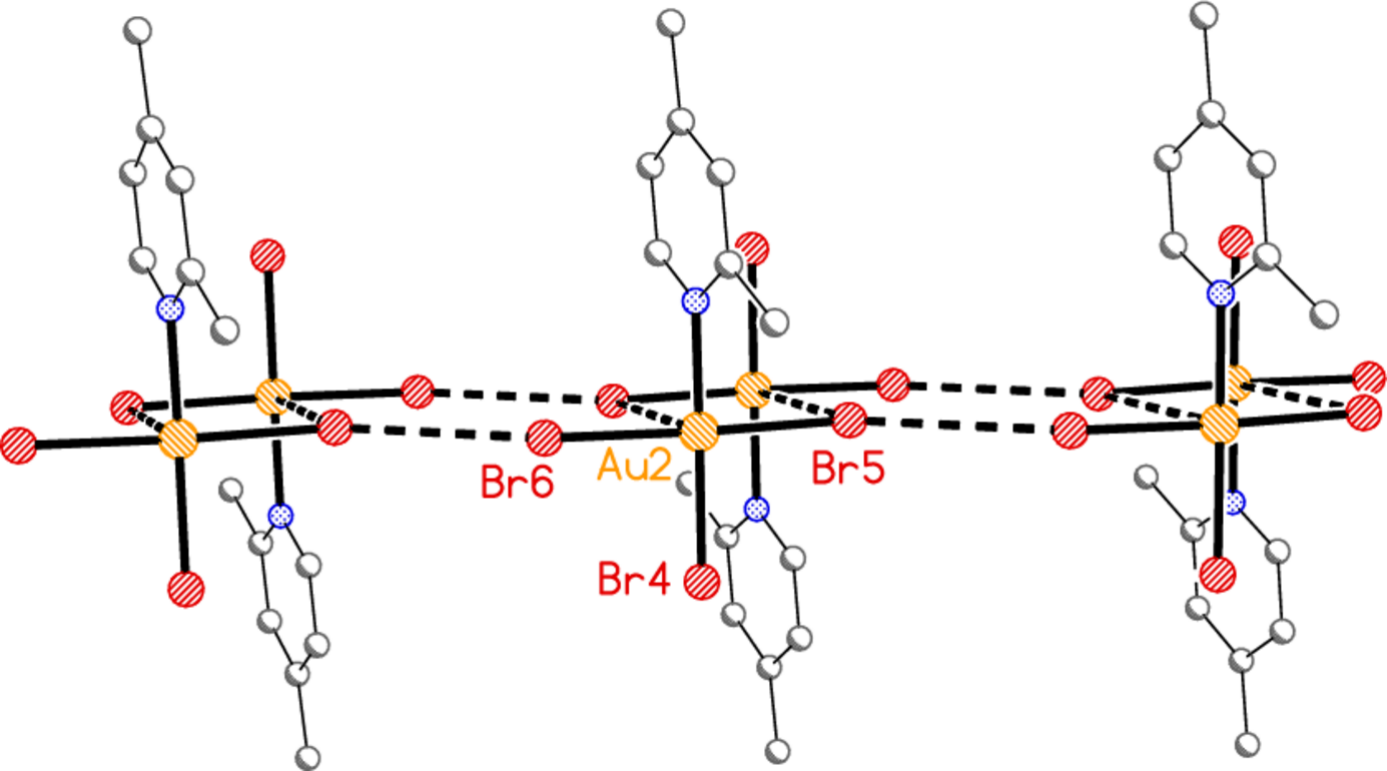
A double chain of mol­ecules 2 for compound **4**, with view direction parallel to the *b* axis. Dashed lines indicate Au⋯Br and Br⋯Br contacts.

**Figure 21:**
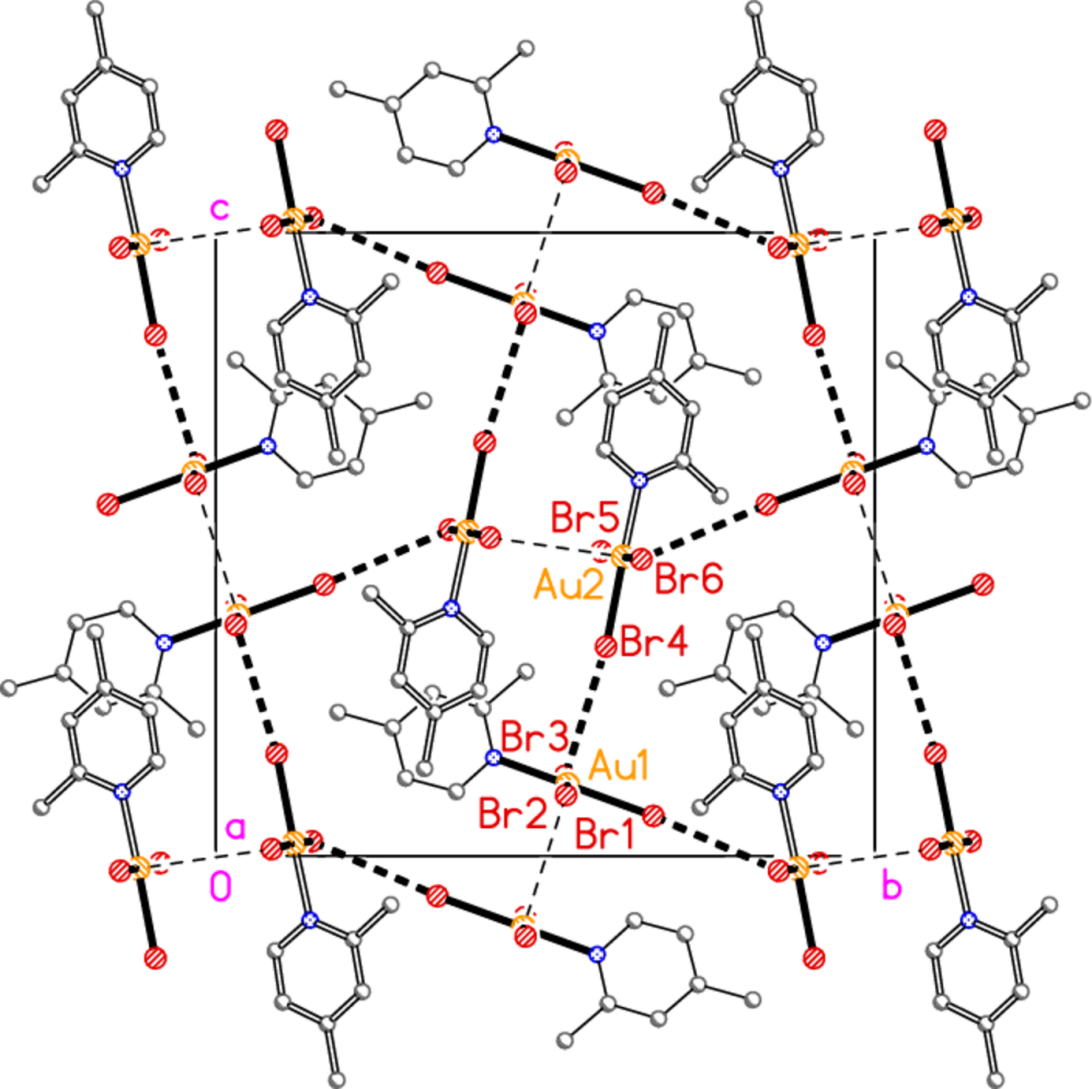
Projection of the structure of compound **4** parallel to the *a* axis. Mol­ecules 2 are indicated by the thicker bonds of the rings. Thick dashed lines indicate Br⋯Br contacts between the double chains of mol­ecule 1 and 2 (in the regions *y* ≃ 0.5, *z* ≃ 0 and *y* ≃ 0.5, *z* ≃ 0.5 respectively, and in regions related to these by symmetry). Thin dashed lines are contacts within the chains, as seen in the previous two figures.

The packing of compound **4** is closely related to that of **1a**. Each independent mol­ecule forms double chains parallel to the *a* axis that are topologically analogous to those of **1a**, with dimers arising from anti­parallel (Au—Br)_2_ contacts and further linked by Br⋯Br contacts; dimensions (Å and °) are Au1⋯Br2(1 − *x*, 1 − *y*, −*z*) = 3.6606 (5), Br2⋯Br3(1 + *x*, *y*, *z*) = 3.3117 (6), Au—Br2⋯Br3′ = 164.67 (2), Au1—Br3⋯Br2′ = 165.45 (2) for the first mol­ecule (Fig. 19 ▸) and Au2⋯Br5(−*x*, 1 − *y*, 1 − *z*) = 3.8328 (5), Br5⋯Br6(−1 + *x*, *y*, *z*) = 3.5191 (6), Au2—Br5⋯Br6′ = 147.93 (2), Au2—Br6⋯Br5′ = 151.00 (2) for the second mol­ecule (Fig. 20 ▸). It is notable that the contact distances are shorter and the angles more linear for mol­ecule 1 than for mol­ecule 2, for reasons that are not apparent. The main difference from **1a** is that the two chains are further linked in **4** by the contacts Br1⋯Br6(*x*, 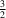 − *y*, −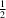 + *z*) = 3.5977 (6) and Br3⋯Br4 = 3.5591 (6) Å (Fig. 21 ▸).

**Figure 22:**
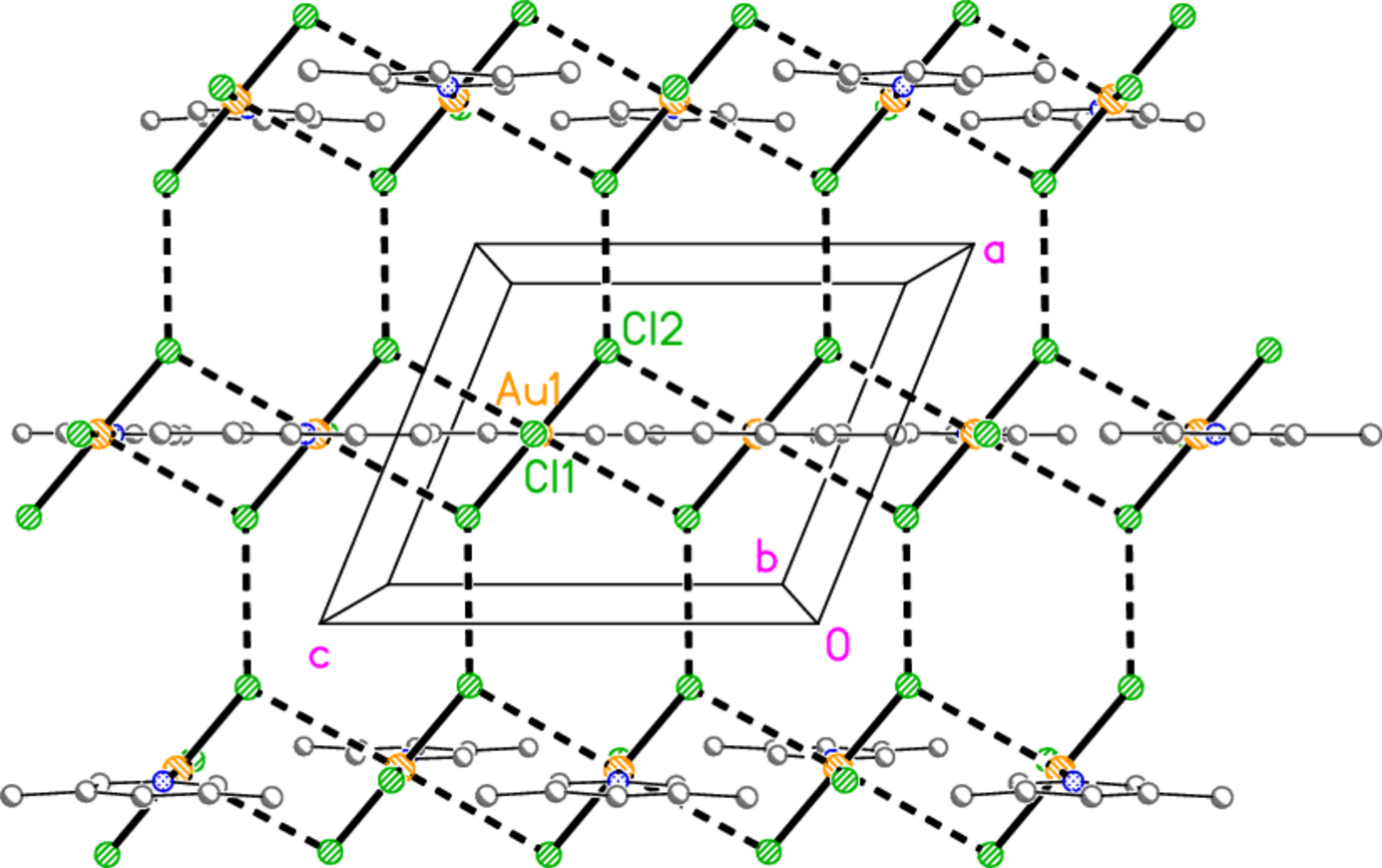
The layer structure of compound **5** viewed parallel to the *b* axis. Dashed lines indicate Au⋯Cl and Cl⋯Cl inter­actions. The Au coordination planes are seen edge-on, so that Au1 obscures Cl1 or *vice versa*.

**Figure 23:**
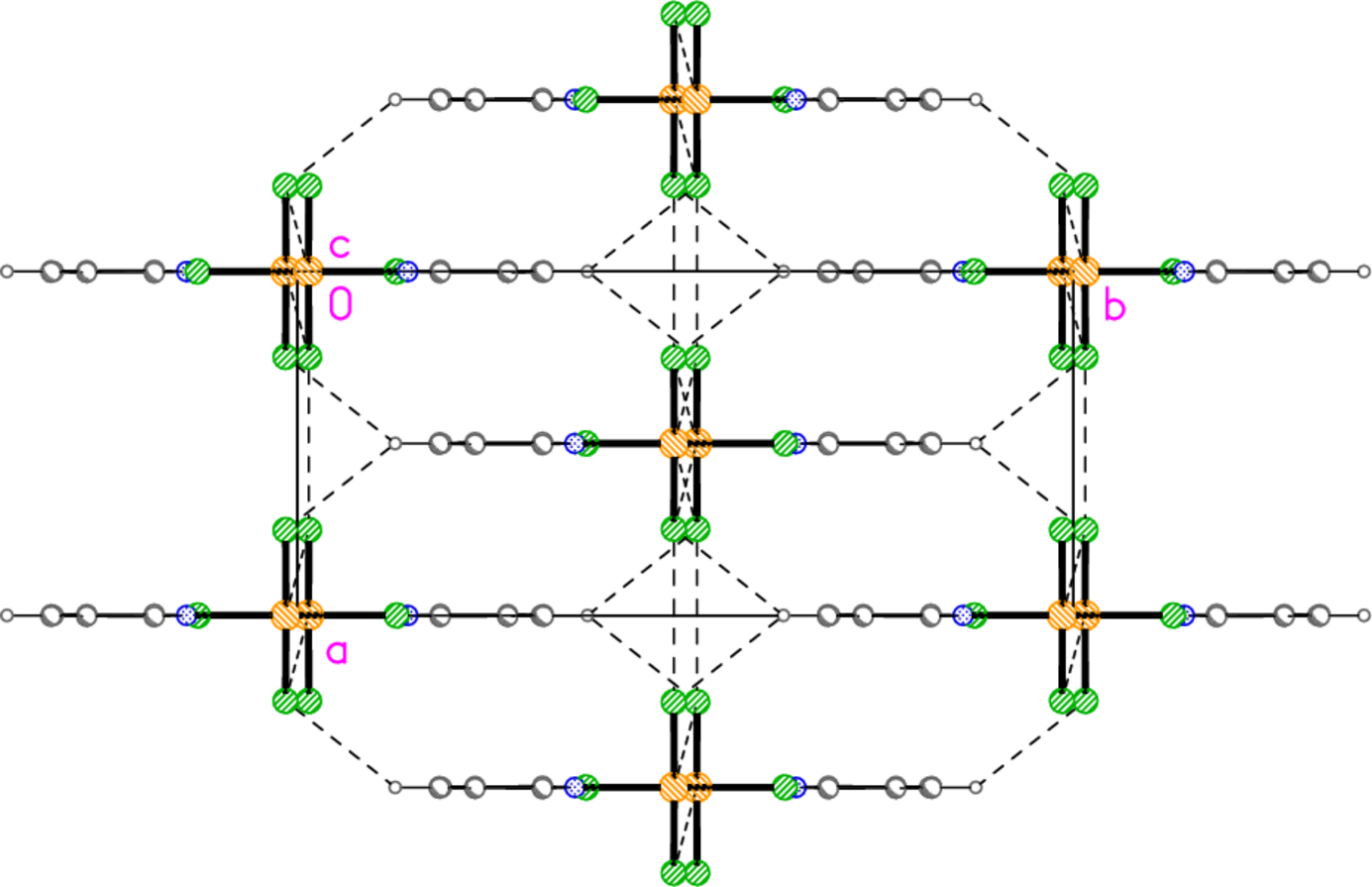
Projection of the structure of compound **5** viewed perpendicular to the *ab* plane. The dashed lines connecting the layers (see Fig. 22 ▸) are the three-centre hydrogen bonds H14⋯Cl2 (with two symmetry-equivalent Cl2 atoms).

The packing of compound **5** resembles that of (py)AuCl in that the mol­ecules are assembled into chains linked by offset stacking, with Au1⋯Cl2(1 − *x*, 1 − *y*, 1 − *z* and *x*, 1 − *y*, 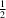 + *z*) = 3.6401 (7) Å, Cl2′⋯Au1⋯Cl2′′ = 163.15 (2)°; the chains run parallel to the *a* axis. However, the Au_2_Cl_2_ quadrilaterals are not edge-linked as in (py)AuCl, but apex-linked. Similar chains were observed in the isotypic pair (4-CN-py)Au*X*_3_ (*X* = Cl or Br; Mohammad-Natij *et al.*, 2013 ▸). Adjacent chains are linked by the contact Cl2⋯Cl2(2 − *x*, *y*, 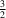 − *z*) = 3.5501 (13) Å to form layers parallel to the *ac* plane (Fig. 22 ▸). The hydrogen bond H2⋯Cl1, 2.67 Å, is not included in Fig. 22 ▸ because of the view direction, in which the rings, lying in or close to the planes at *x* ≃ 0, 0.5, 1 *etc*., are seen edge-on. Layers are connected in the **b** direction by the three-centre hydrogen bond from H14 to two Cl2 atoms (Fig. 23 ▸).

**Figure 24:**
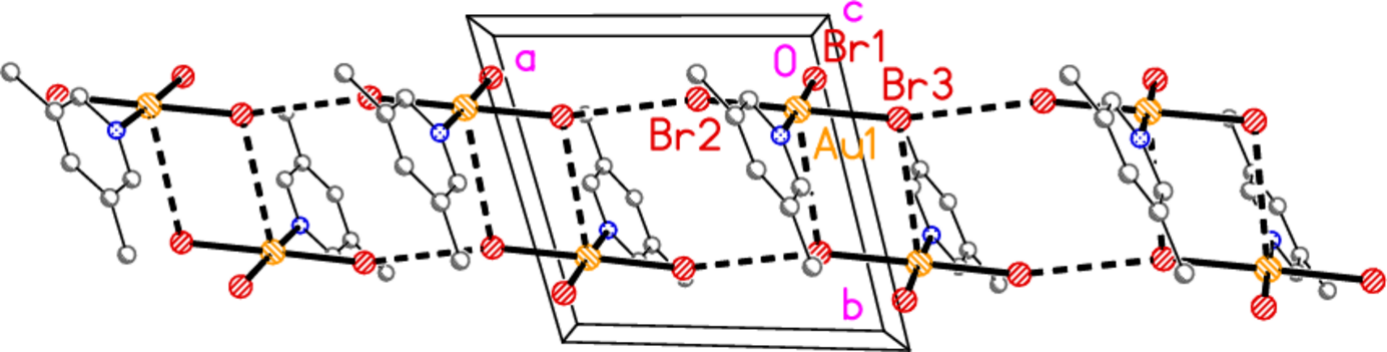
A double chain of compound **6**, viewed parallel to the *c* axis. Dashed bonds indicate Au⋯Br or Br⋯Br inter­actions.

**Figure 25:**
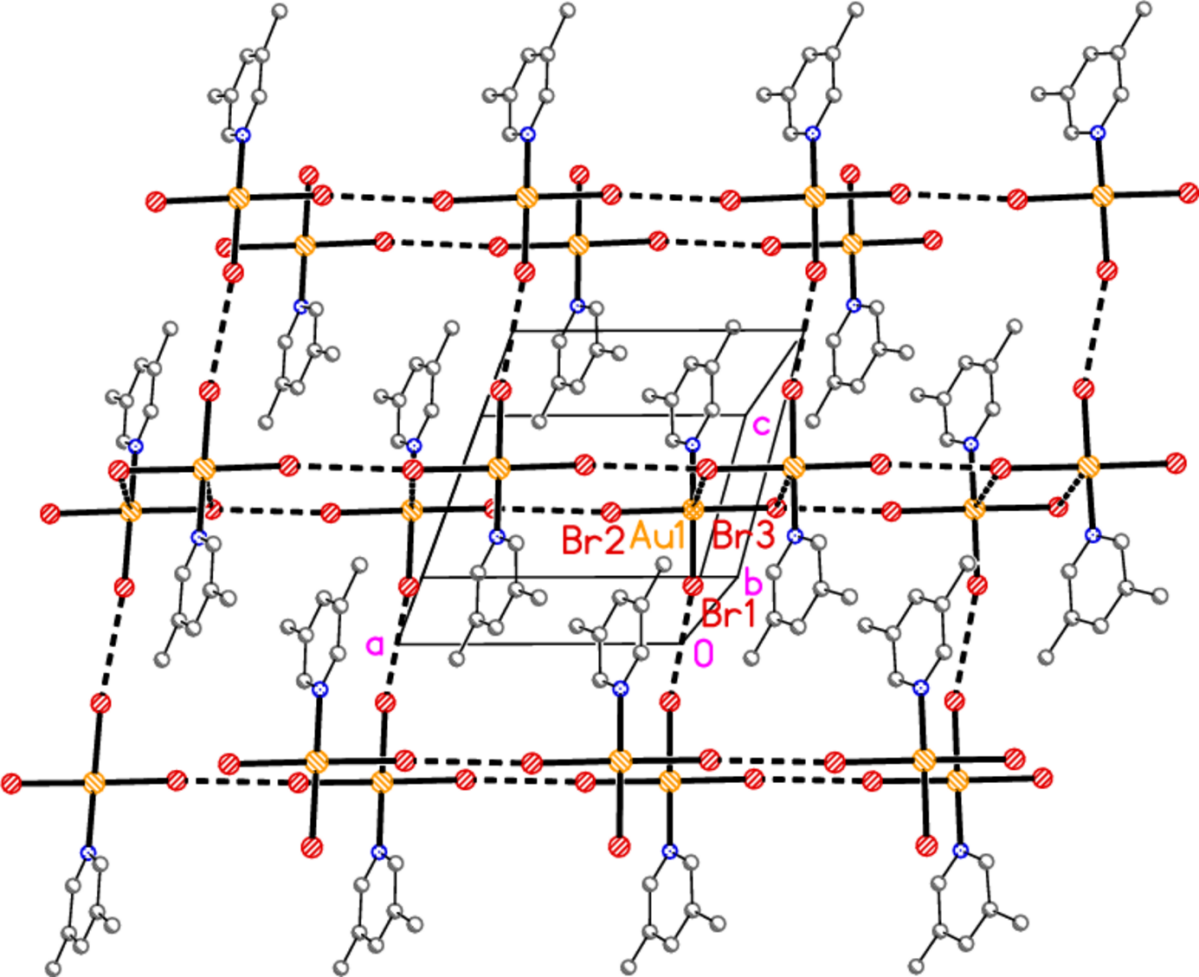
A double layer of compound **6** parallel to the plane (0
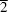
1). The double chains of Fig. 24 ▸ are linked by Br1⋯Br1 contacts [approximately vertical in this view, which is rotated by *ca* 20° around the horizontal axis from the direction perpendicular to (0
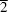
1)].

The packing of compound **6** involves double chains, parallel to the *a* axis (Fig. 24 ▸) that are topologically the same as those of **1a** and **4**. The usual dimers are formed, although the contact distance is rather long: Au1⋯Br3(−*x*, 1 − *y*, 1 − *z*) = 3.7738 (6) Å. The dimers are connected by the contacts Br2⋯Br3(1 + *x*, *y*, *z*) = 3.4644 (6) Å, with angles Au1—Br2⋯Br3′ = 165.93 (2)° and Au1—Br3⋯Br2′ = 166.15 (2)°. The double chains are connected by the contacts Br1⋯Br1(−*x*, −*y*, −*z*) = 3.4284 (9) Å, with Au1—Br1⋯Br1′ = 156.36 (3)°, to form a double layer parallel to (0
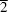
1) (Fig. 25 ▸).

**Figure 26:**
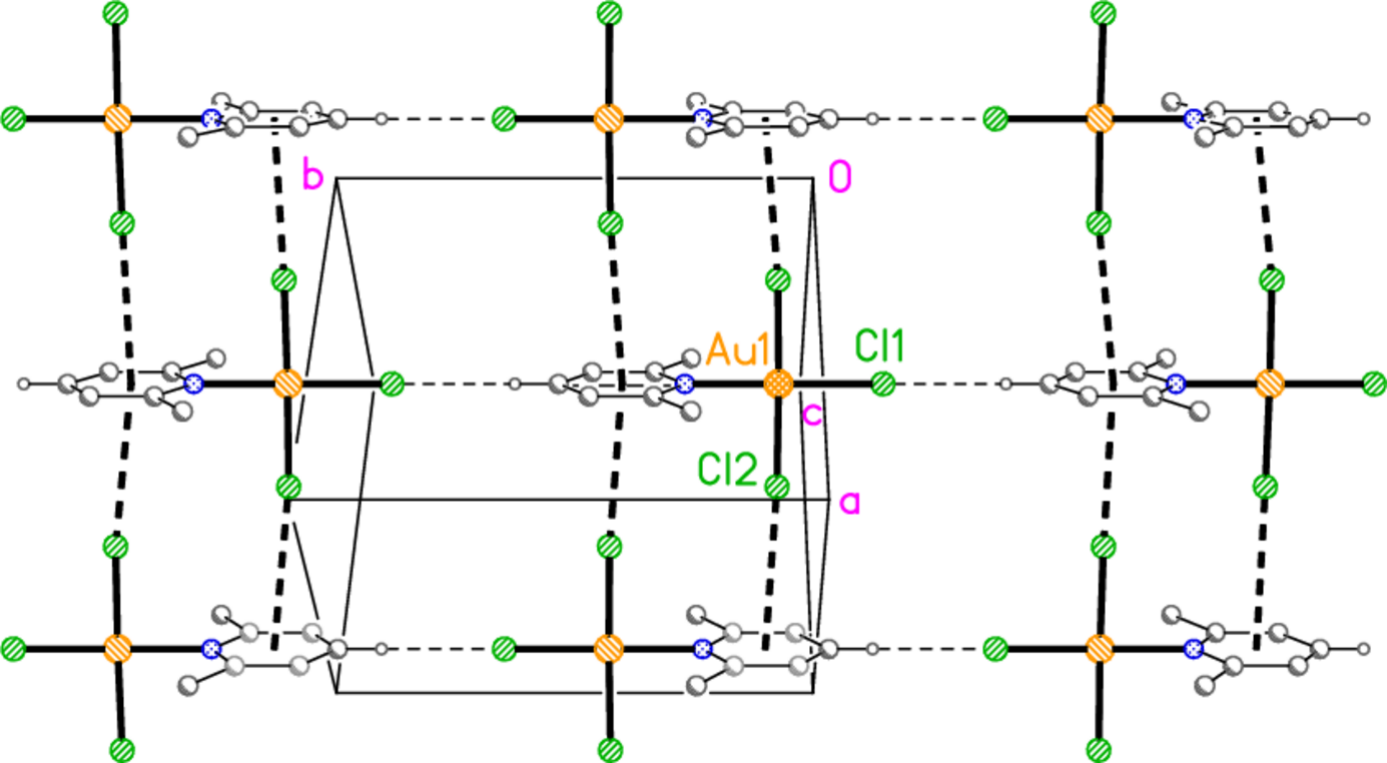
The layer structure of compound **7** viewed perpendicular to (10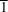). The dashed lines indicate H⋯Cl hydrogen bonds (thin) or Cl⋯π inter­actions (thick).

The packing of compound **7** is unexpected; it involves neither Au⋯Cl nor Cl⋯Cl inter­actions. Instead, the two important contacts are the short hydrogen bond H14⋯Cl1′(*x*, 1 + *y*, *z*) and a Cl⋯π contact from Cl2 to the centroid (*Cg*) of the pyridine ring at (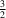 − *x*, 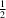 − *y*, 1 − *z*); the contact distance Cl2⋯*Cg*′ is 3.5458 (5) Å, with angles Au1—Cl2⋯*Cg*′ = 162.6° and *Cg*′⋯Cl2⋯*Cg*" = 171.4°. The Cl⋯π inter­actions propagate parallel to [101], so that the result is a layer structure parallel to (10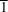) (Fig. 26 ▸). This type of inter­action can be regarded as a halogen bond from the chlorine atom to the π electron cloud of the pyridine ligand.

**Figure 27:**
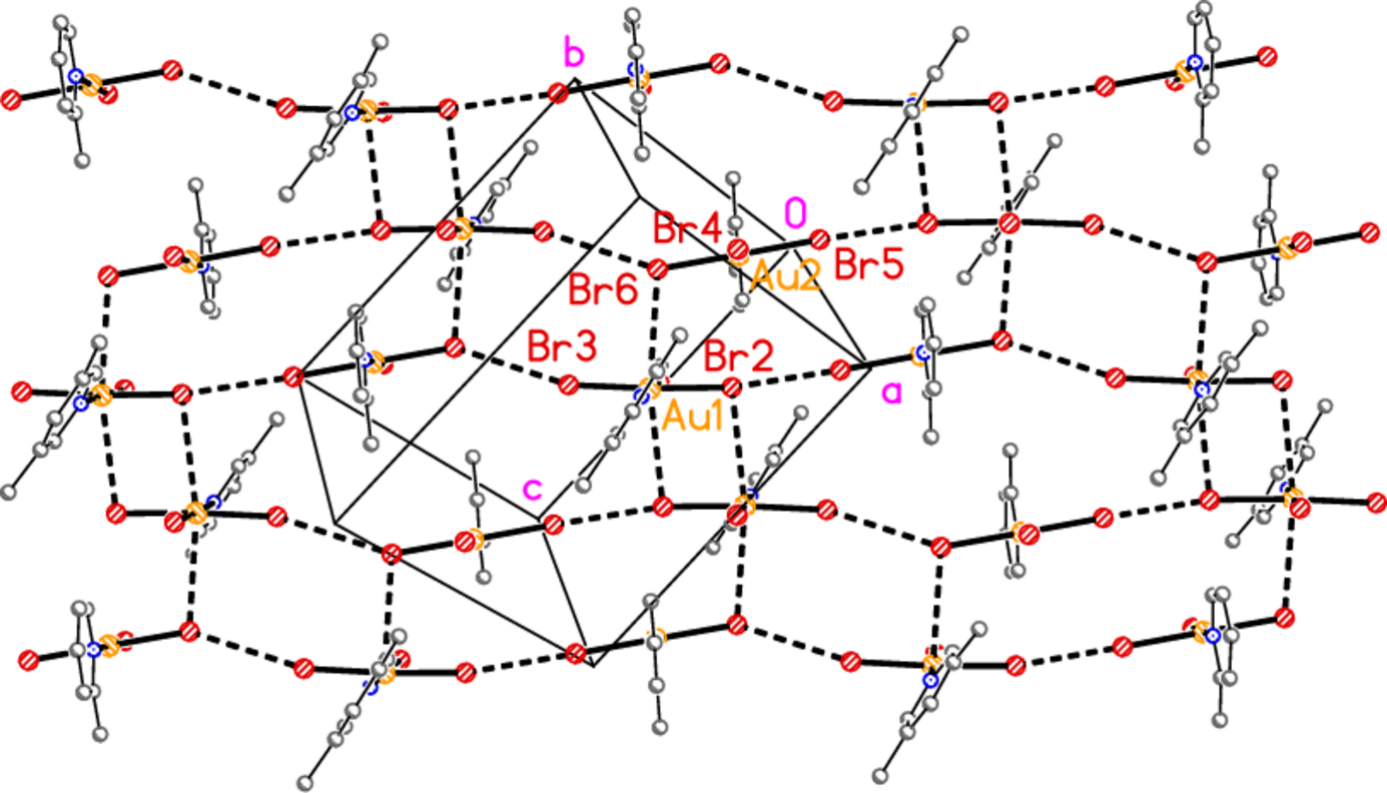
The layer structure of adduct **8** viewed perpendicular to (110). The dashed lines indicate Au⋯Br or Br⋯Br contacts.

**Figure 28:**
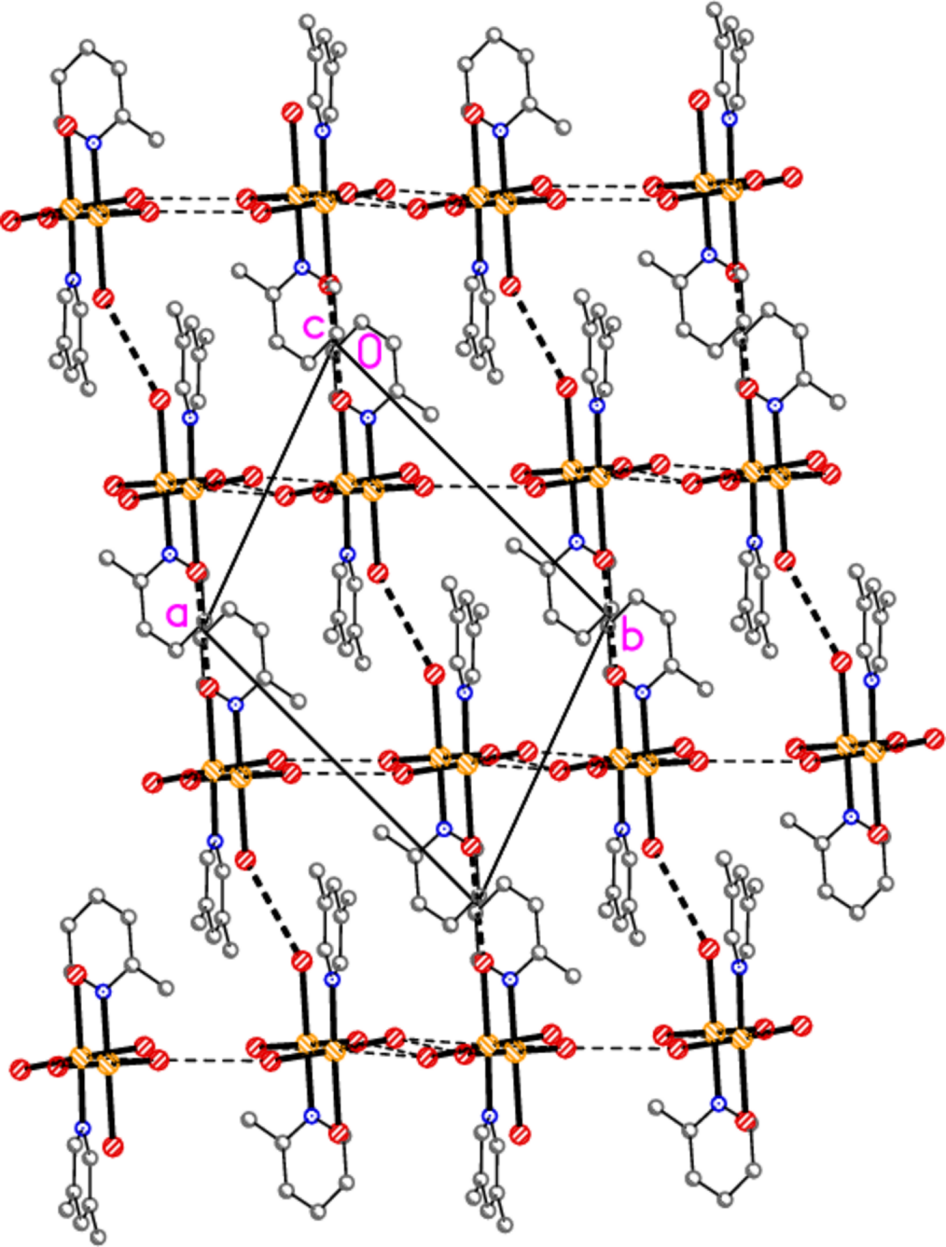
The links between the layers (seen edge-on) of adduct **8** are provided by the contacts Br1⋯Br1′ and Br4⋯Br4′, drawn with thick dashed lines. The former are almost exactly vertical in this diagram; the latter are slightly angled to the vertical direction. The view direction is parallel to the *c* axis.

The main feature of the packing of adduct **8** (composed of **2** and **6**) is a layer structure (Fig. 27 ▸) parallel to (110). Chains of alternating mol­ecules of **2** and **6**, horizontal in Fig. 27 ▸, run parallel to [1
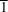

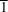
]; they are propagated by the contacts Br2⋯Br5(1 − *x*, −*y*, −*z*) = 3.2915 (11) Å and Br3⋯Br6(−*x*, 1 − *y*, 1 − *z*) = 3.5493 (13) Å, with Au1—Br2⋯Br5′ = 161.86 (5), Au2—Br5⋯ Br2′ = 169.76 (5), Au1—Br3⋯Br6′ = 162.49 (5) and Au2—Br6⋯Br3′ = 151.56 (5)°. As in the structure of **2** alone, there are no axial contacts to the gold atom Au2 [discounting Au2⋯Br2 3.9093 (11) Å as too long]. The gold atom of mol­ecule **6** has two axial contacts, Au1⋯Br2(1 − *x*, −*y*, 1 − *z*) = 3.5279 (10) and Au1⋯Br6 = 3.5169 (10) Å, with Br2′⋯Au1⋯Br6 = 169.74 (2)°, in contrast to its single axial contact in the structure of **6** alone. The former contact is part of an offset-stacked dimer (see the small quadrilaterals in Fig. 27 ▸), but these quadrilaterals do not associate directly to form more extensive elements of the packing. The linkages between layers are provided by the contacts Br1⋯Br1(−*x*, −*y*, 1 − *z*) = 3.362 (2) and Br4⋯Br4(1 − *x*, 1 − *y*, −*z*) = 3.343 (2) Å, with Au1—Br1⋯Br1′ = 175.87 (7) and Au2—Br4⋯Br4′ = 153.75 (6)° (Fig. 28 ▸), in a manner reminiscent of the inter­layer links in **3** and those within the double layers of **6**.

## 4. Database survey

The searches employed the routine ConQuest (Bruno *et al.*, 2002 ▸), part of Version 2024.1.0 of the CSD (Groom *et al.*, 2016 ▸). A search for ‘simple’ compounds of the form *L*AuCl_3_ (*L* = pyridine ligand with no substituents involved in further rings, *X* = halogen) gave 21 hits. The Au—N bond lengths were 2.015–2.073, av. 2.043 (13) Å, the Au—Cl bond lengths *trans* to N were 2.255–2.273, av. 2.263 (3) Å, and the Au—Cl bond lengths *cis* to Au—N were 2.221–2.29, av. 2.275 (11) Å. No clear *trans* influences can be recognised in these values. The three hits for *X* = Br were the (py)AuBr_3_ component of {[(py)_2_AuBr_2_]^+^[AuBr_4_]^−^·[(py)AuBr_3_]} (WOQMEU, Peters *et al.*, 2000 ▸); (4-CN-py)AuBr_3_ (WIRFUA, Mohammad-Natij *et al.*, 2013 ▸); and (3-F-py)AuBr_3_ (WEFRAE, Pizzi *et al.*, 2022 ▸). All showed Au—Br_*trans*_ bonds significantly shorter than Au—Br_*cis*_, by *ca* 0.02–0.03 Å, but the Au—N bond lengths were variable at 2.040–2.098 Å. The sample is probably too small to draw reliable conclusions.

It is instructive to take six of the simplest compounds thus found and briefly compare their packing features with those of **1**–**8**. The compounds chosen are: *L* = 4-ethyl­pyridine, *X* = Cl (ESITIM, Hobbollahi *et al.*, 2019 ▸); *L* = 3-bromo­pyridine, *X* = Cl (WEFQAD); *L* = 3-fluoro­pyridine, *X* = Cl (WEFQEH); *L* = 3-chloro­pyridine, *X* = Cl (WEFQIL); *L* = 3-iodo­pyridine, *X* = Cl (WEFQOR); and *L* = 3-fluoro­pyridine, *X* = Br (WEFRAE; all from Pizzi *et al.*, 2022 ▸). In all cases, the authors drew attention to the short Au⋯*X* contacts. These compounds are included in Table 19 ▸. C—H⋯*X* hydrogen bonding is neglected.

**Figure 29:**
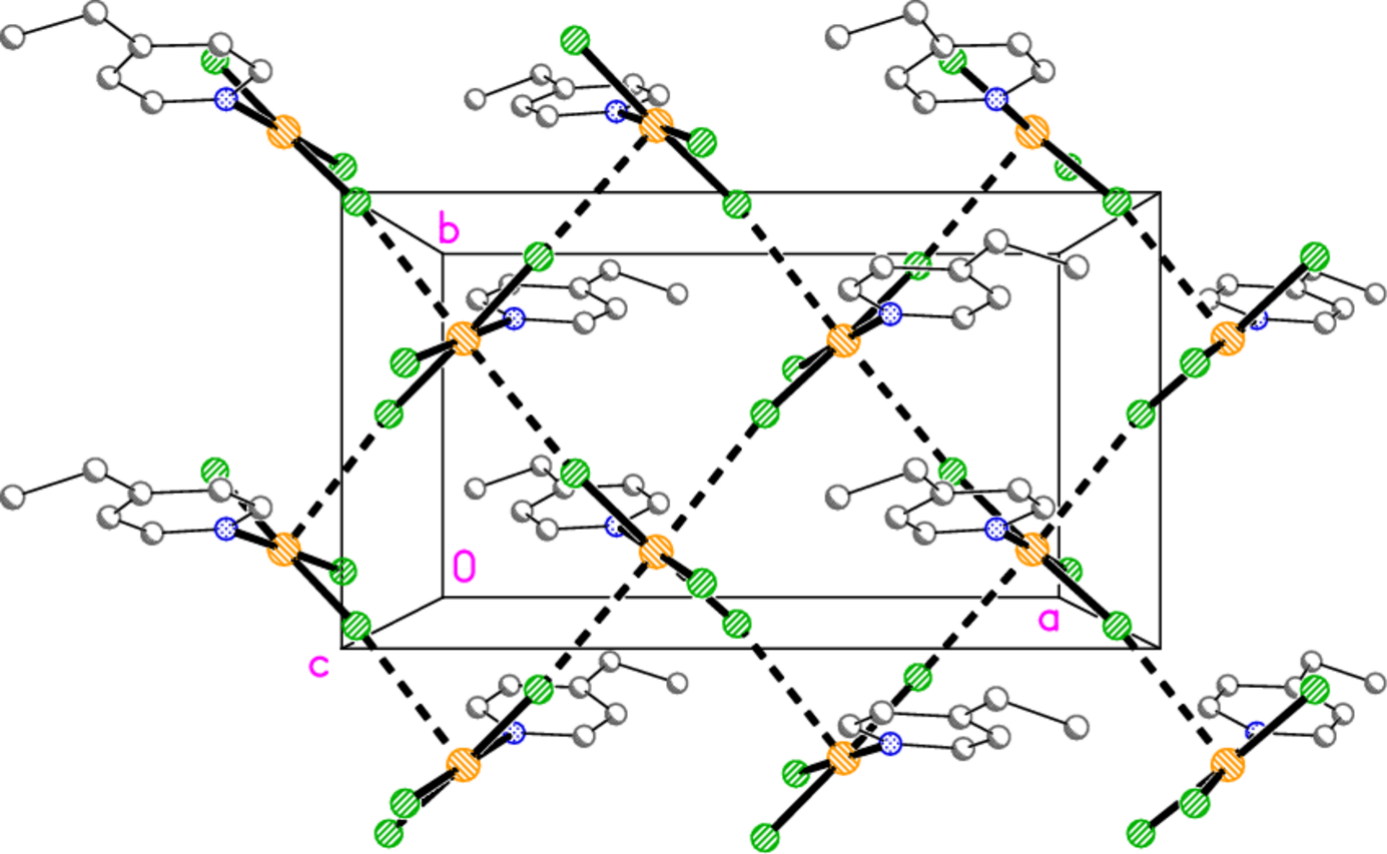
The packing of ESITIM, tri­chlorido­(4-ethyl­pyridine)­gold(III), viewed parallel to the *c* axis in the region *z* ≃ 0.75. Dashed lines indicate Au⋯Cl contacts.

**Figure 30:**
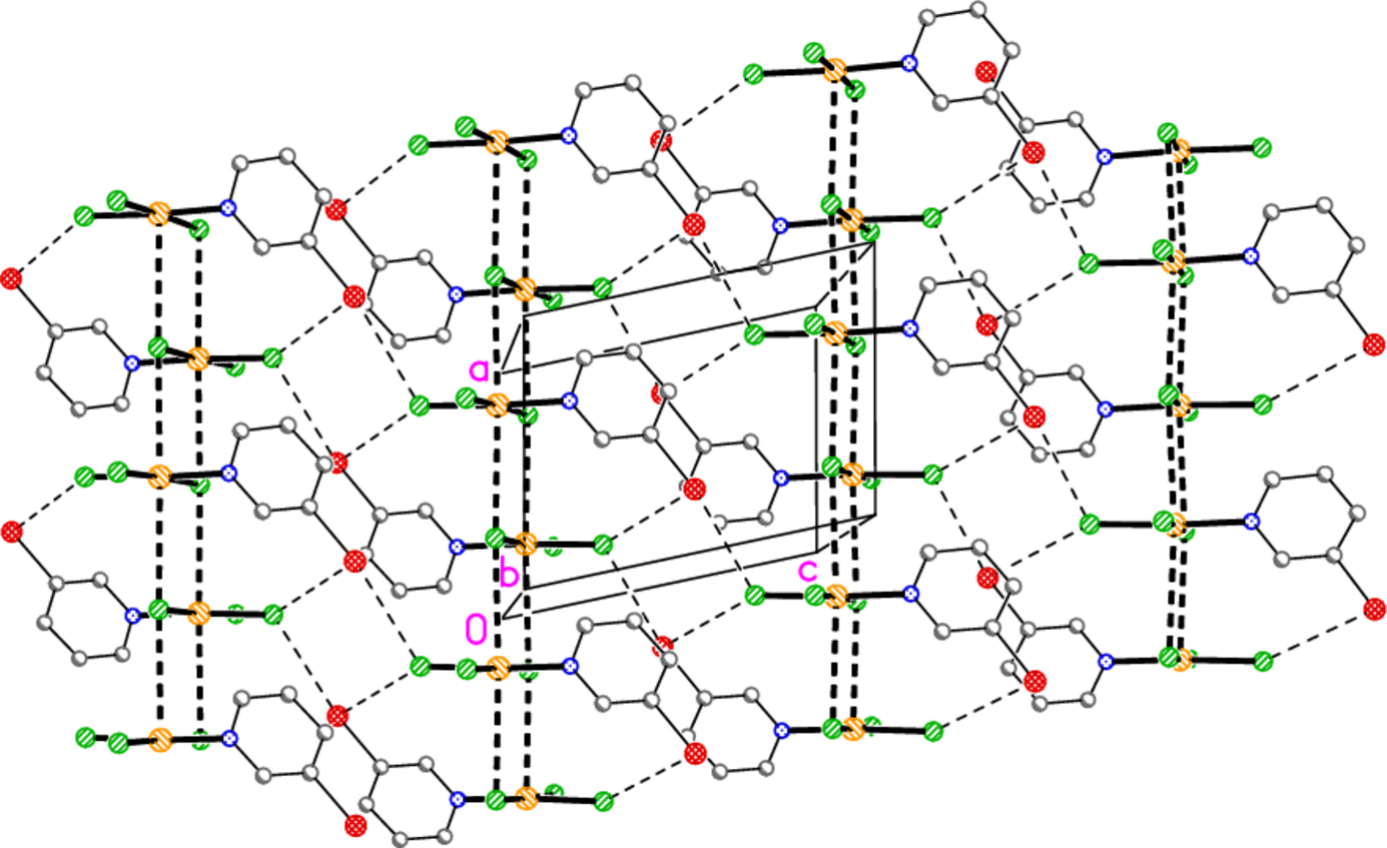
The packing of WEFQAD, (3-bromo­pyridine)­tri­chlorido­gold(III), viewed perpendicular to the *ac* plane. Dashed lines indicate Au⋯Cl (thick) or Br⋯Cl (thin) contacts.

**Figure 31:**
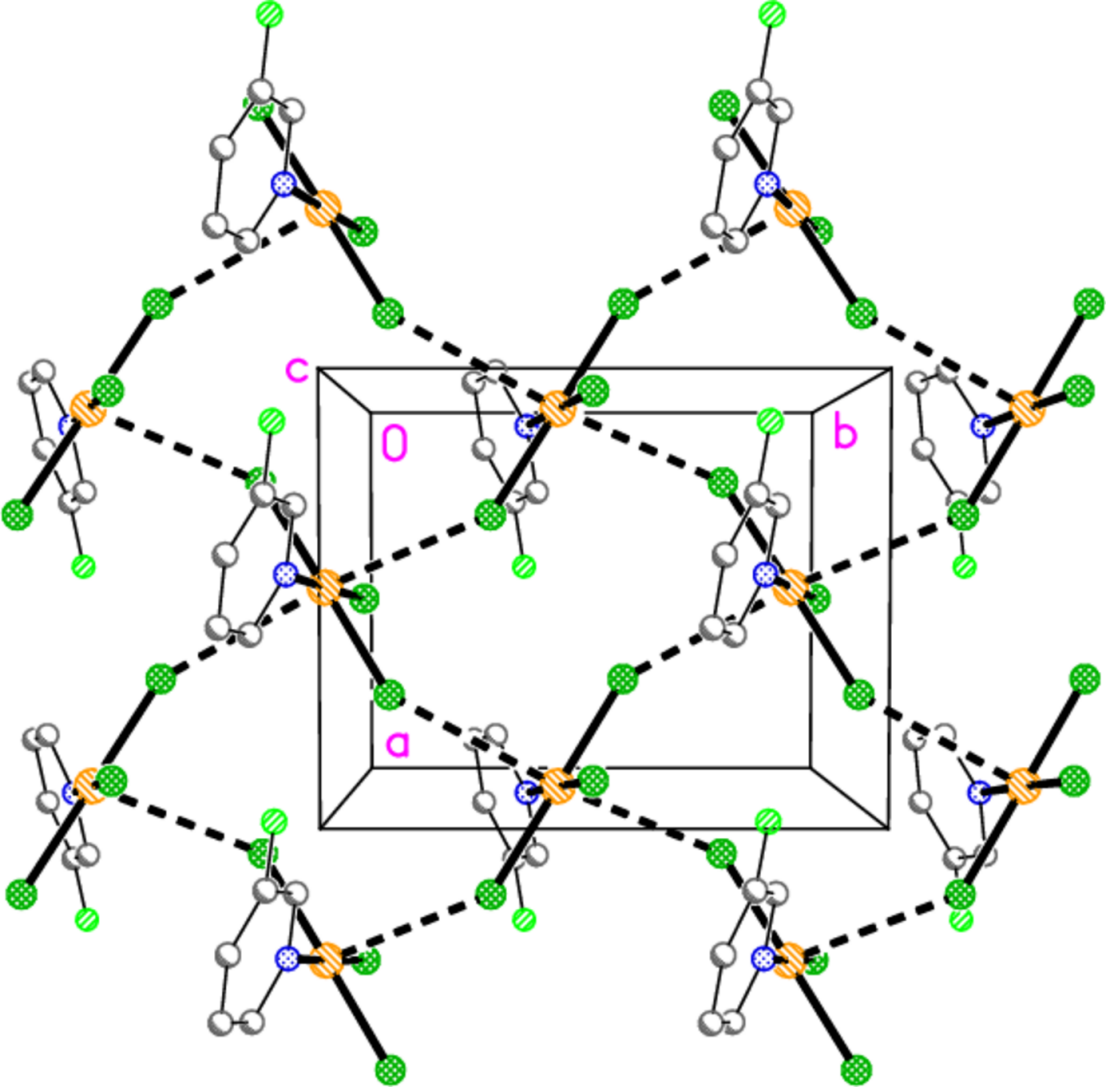
The packing of WEFQEH, tri­chlorido­(3-fluoro­pyridine)­gold(III), viewed perpendicular to the *ab* plane in the region *z* ≃ 0.25. Dashed lines indicate Au⋯Cl contacts. Fluorine atoms are the smaller green circles. This is a redrawn version of Fig. 2 ▸ of Pizzi *et al.* (2022 ▸).

**Figure 32:**
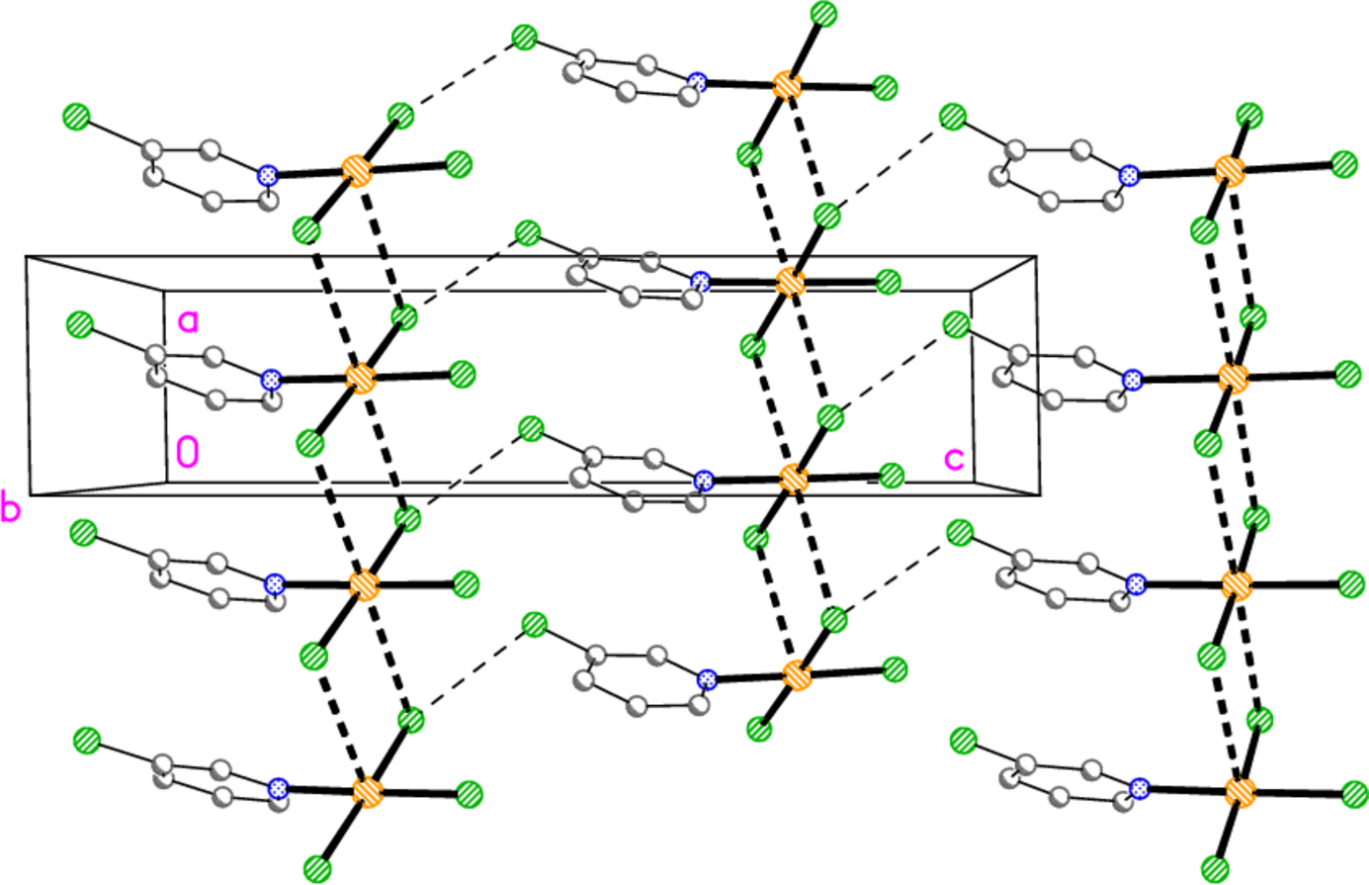
The packing of WEFQIL, tri­chlorido­(3-chloro­pyridine)­gold(III), viewed perpendicular to the *ac* plane in the region *y* ≃ 0.25. Dashed lines indicate Au⋯Cl (thick) or Cl⋯Cl (thin) contacts.

**Figure 33:**
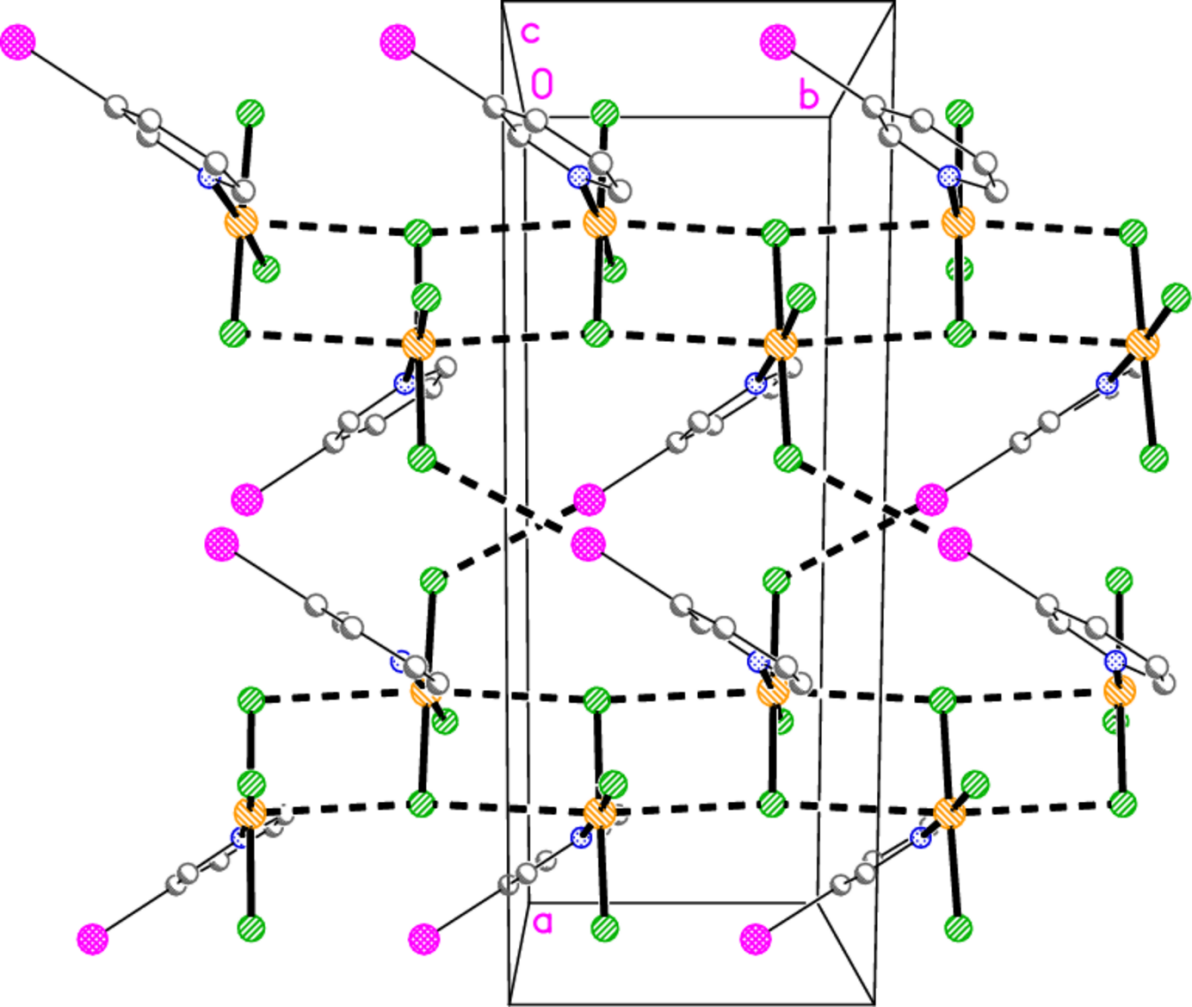
The packing of WEFQOR, tri­chlorido­(3-iodo­pyridine)­gold(III), viewed perpendicular to the *ab* plane in the region *z* ≃ 0.75. Dashed lines indicate Au⋯Cl or I⋯Cl contacts. Iodine atoms are coloured violet.

**Figure 34:**
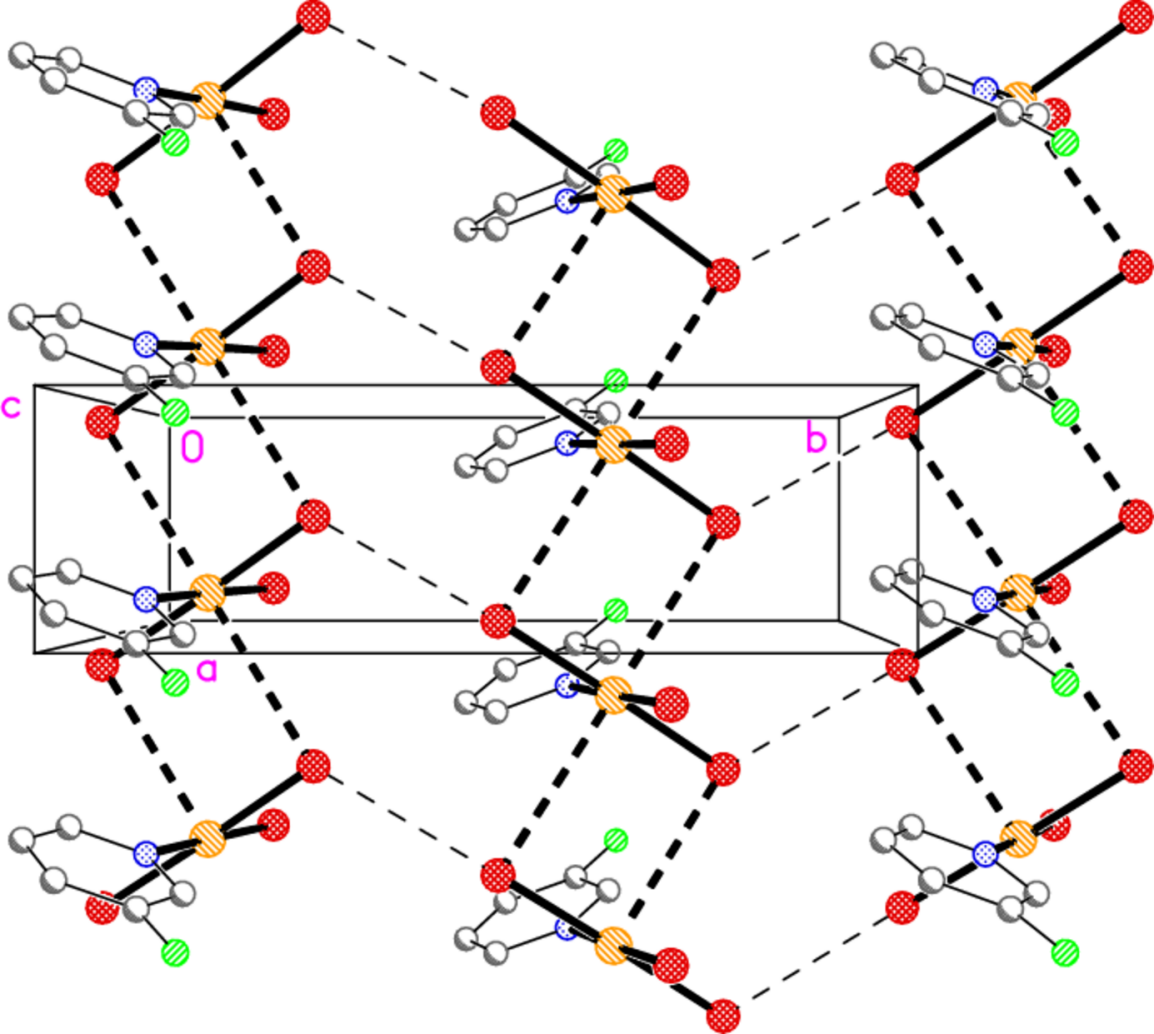
The packing of WEFRAE, tri­bromido­(3-fluoro­pyridine)­gold(III), viewed perpendicular to the *ab* plane in the region *z* ≃ 0.75. Dashed lines indicate Au⋯Br (thick) or Br⋯Br (thin) contacts. Fluorine atoms are coloured green.

ESITIM crystallizes in *Pcab* with *Z* = 8. In the original publication, the Au⋯Cl contacts (3.244, 3.409 Å) were described as linking the mol­ecules to form infinite chains. In fact, they combine to form a layer structure, involving Au_4_Cl_4_ rings, parallel to the *ab* plane at *z* ≃ 0.25, 0.75 (Fig. 29 ▸). In the series of 3-halo­pyridine complexes, the halogen substituents of the pyridine rings are ‘non-innocent’ atoms as regards to inter­molecular inter­actions. In WEFQAD (*P*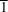, *Z* = 2), the Au⋯Cl contacts (3.492, 3.579 Å) combine to form a ‘ladder’ structure parallel to the *a* axis. Two short Br⋯Cl contacts to the *trans* chlorine atom (3.490, 3.690 Å) are observed, which link the layers (Fig. 30 ▸). In the corresponding 3-fluoro derivative WEFQEH (*P*2_1_/*n, Z* = 4), the Au⋯Cl contacts (3.373, 3.426 Å) combine to form a layer structure, involving Au_4_Cl_4_ rings, parallel to the *ab* plane at *z* ≃ 0.25, 0.75 (Fig. 31 ▸; an equivalent diagram was presented by Pizzi *et al.* (2022 ▸) but we include this Figure for completeness and for consistency of format). A short F⋯F contact of 2.684 Å links the layers. In the 3-chloro derivative WEFQIL (*P*2_1_/*n*, *Z* = 4), the Au⋯Cl contacts (3.402, 3.412 Å) combine to form a chain of apex-linked quadrilaterals (analogous to the chains in **5**) parallel to the *a* axis; these are liked by Cl⋯Cl_py_ contacts of 3.536 Å to form layers parallel to the *ac* plane at *y* ≃ 0.25, 0.75 (Fig. 32 ▸), and the layers are connected in the third dimension by another Cl⋯Cl_py_ contact of 3.495 Å. In the 3-iodo derivative WEFQOR (*C*2/*c*, *Z* = 8), the Au⋯Cl contacts (3.368, 3.483 Å) combine to form a ‘ladder’ structure parallel to the *b* axis; ladders are linked by I⋯Cl contacts of 3.500 Å to form layers parallel to the *ab* plane at *z* ≃ 0.25, 0.75 (Fig. 33 ▸). The layers are linked in the third dimension by a Cl_*trans*_⋯Cl_*trans*_ contact of 3.433 Å. In WEFRAE (*P*2_1_2_1_2_1_, *Z* = 4), the tri­bromido analogue of WEFQEH, the Au⋯Br contacts (3.542, 3.588 Å) combine to form a chain of apex-linked quadrilaterals parallel to the *a* axis; these are linked directly by quite long Br⋯Br contacts of 3.710 Å to form a layer structure parallel to the *ab* plane at *z* ≃ 0.25, 0.75 (Fig. 34 ▸). Layers are linked in the third dimension by Br_*trans*_⋯Br_*trans*_ contacts of 3.712 Å. The fluorine atom is not involved in short contacts.

A search for Au^III^ structures related to **7**, containing Au—Cl and Au—N_pyridinic_ bonds together with a short Cl⋯π contact (defined by the distance from Cl to the pyridine ring centroid *Cg*) gave thirteen hits with Cl⋯*Cg* < 3.7 Å. The shortest distance is 3.344 Å in tri­chlorido-(1,7,15,15-tetra­methyl-3,10-di­aza­tetra­cyclo­[10.2.1.0^2,11^.0^4,9^]penta­deca-2,4,6,8,10-penta­ene)gold(III), a camphorquinoxaline complex (SUYXAN; Glišić *et al.*, 2018 ▸).

## 5. Synthesis and crystallization

*Tri­chlorido­(2-methyl­pyridine)­gold(III)* (**1**): 114.2 mg (0.351 mmol) of the gold(I) precursor chlorido­(2-methyl­pyridine)­gold(I) was prepared by the method of Ahrens (1999 ▸). This was dissolved in 5 ml of di­chloro­methane, and the solution was added to a solution of 100 mg (0.363 mmol) of PhICl_2_ in 5 ml of di­chloro­methane. Equal (0.4 ml) portions of the solution were transferred to five ignition tubes and overlayered with the five precipitants *n*-pentane, *n*-heptane, diethyl ether, diisopropyl ether and petroleum ether (b.p. 313–333 K). The tubes were stoppered and transferred to the refrigerator overnight. Crystals of compound **1**, polymorph **a**, were obtained as yellow prisms and tablets from the tube with diisopropyl ether. In general for these syntheses, crystals also formed in at least some of the other tubes, but the best, judged by inspection under a microscope, were selected for X-ray measurements. Elemental analysis [%]: calc.: C 18.18, H 1.78, N 3.53; found C 17.78, H 1.79, N 3.59. Because of the problem of incomplete oxidation that we have sometimes encountered using PhICl_2_, the procedure was repeated in parallel using two equivalents of PhICl_2_, although this precaution later proved to have been unnecessary for the reactions presented here. The same crystallization experiments were carried out. Crystals of compound **1**, polymorph **b**, were obtained as yellow plates from the tube with *n*-pentane. Elemental analysis [%]: calc.: C 18.18, H 1.78, N 3.53; found: C 17.63, H 1.78, N 3.58.

*Tri­bromido­(2-methyl­pyridine)­gold(III)* (**2**): 90 mg (0.247 mmol) of (tht)AuBr (tht = tetra­hydro­thio­phene) were converted to bis­(2-methyl­pyridine)­gold(I) di­bromido­aurate(I) (Döring & Jones, 2024*a* ▸), which was immediately (without drying) dissolved in 2 ml of di­chloro­methane, and two drops of elemental bromine were added. The usual crystallization experiments were carried out. Crystals of compound **2** were obtained in the form of red blocks and tablets from the tube with *n*-pentane. Elemental analysis [%]: calc: C 13.60, H 1.33, N 2.64; found: C 12.60, H 1.37, N 2.62.

*Tri­bromido­(3-methyl­pyridine)­gold(III)* (**3**): 90 mg (0.247 mmol) of (tht)AuBr were converted to bis­(3-methyl­pyridine)­gold(I) di­bromido­aurate(I) (Döring & Jones, 2024*a* ▸), which was immediately (without drying) dissolved in 2 ml of di­chloro­methane. Two drops of elemental bromine were added. The usual crystallization experiments were carried out. Crystals of compound **3** were obtained in the form of red plates from the tube with *n*-pentane. Elemental analysis [%]: calc.: C 13.60, H 1.33, N 2.64; found: C 13.47, H 1.35, N 2.78.

*Tri­bromido­(2,4-di­methyl­pyridine)­gold(III)* (**4**): 45,2 mg (0.124 mmol) of (tht)AuBr were dissolved in 2 ml of 2,4-di­methyl­pyridine. The solution was transferred to a 5 ml glass vial and overlayered with diisopropyl ether. The vial was closed and stored in the refrigerator. The supernatant was then pipetted off and the remaining colourless crystals, assumed to be bis­(2,4-di­methyl­pyridine)­gold(I) di­bromido­aurate(I), dried *in vacuo*, yielded 32.5 mg (48%). The crystals proved to be unsuitable for structure determination because of streaking of the diffraction peaks. They were dissolved in 2 ml of di­chloro­methane and 3 drops of elemental bromine were added, leading to a red solution. This was overlayered with *n*-pentane and stored in the refrigerator for a week. Crystals of **4** were obtained in the form of red plates and needles. The elemental analysis gave an unsatisfactory value for C: [%] calc.: C 15.46, H 1.67, N 2.58; found: C 13.81, H 1.53, N 2.43.

*Tri­chlorido­(3,5-di­methyl­pyridine)­gold(III)* (**5**): 166 mg (0.518 mmol) of (tht)AuCl were converted to bis­(3,5-di­methyl­pyridine)­gold(I) di­chlorido­aurate(I) (Döring & Jones, 2024*a* ▸). The sample was divided in half; each half was dissolved in 5 ml of di­chloro­methane, and then treated with one or two equivalents of PhICl_2_. The solutions were subjected to the usual crystallization experiments. Crystals of **5** were obtained in the form of yellow blocks from all tubes; those chosen were from the 1:2 experiment using diethyl ether. Elemental analysis [%]: calc.: C 20.48, H 2.21, N 3.41; found: C 20.23, H 2.121, N 3.58.

*Tri­bromido­(3,5-di­methyl­pyridine)­gold(III)* (**6**): see (**8**) below.

*Tri­chlorido­(2,6-di­methyl­pyridine)­gold(III)* (**7***)*: 122.5 mg (0.382 mmol) of (tht)AuCl were converted to 119 mg (0.175 mmol) of (2,6-di­methyl­pyridine)­gold(I) di­chloro­aurate(I) (Hashmi *et al.*, 2010 ▸)**.** This was divided into two portions, and 2 ml of di­chloro­methane were added to each. A solution of 24.1 mg (0.088 mmol) of PhICl_2_ in 2 ml of di­chloro­methane was added to one aliquot and a solution of 48.2 mg (0.175 mmol) of PhICl_2_ in 2 ml of di­chloro­methane to the other. These solutions were subjected to the usual crystallization experiments. Crystals of **7** in the form of yellow blocks were obtained from the 1:2 experiment using *n*-heptane. Elemental analysis [%]: calc.: C 20.48, H 2.21, N 3.41; found: C 20.71, H 2.20, N 3.53.

*Tri­bromido­(2-methyl­pyridine)­gold(III)/tri­bromido­(3,5-di­methyl­pyridine)­gold(III) (1/1)* (**8**): Crystals of compounds **8** and **6** arose serendipitously, partly as a result of human error, as follows. 137.3 mg (0.376 mmol) of (tht)AuBr were converted to 84.0 mg (0.114 mmol) of bis­(2-methyl­pyridine)­gold(I) di­bromo­aurate(I) as above, of which 75.1 mg (0.102 mmol) were dissolved in 5 ml of di­chloro­methane. Five drops of elemental bromine were added. Half of the resulting red solution was overlayered with *n*-pentane. At some stage, which can no longer be identified (but the 2-picoline was checked by NMR and was pure), the system became contaminated with 3,5-di­methyl­pyridine. One of the red crystals that formed was investigated and proved to be the 1/1 adduct **8**. The ^1^H NMR spectrum of the sample showed the expected two methyl singlets, but in the ratio 4:1 rather than the expected 2:1 for a 1/1 mixture of **2** and **6**; this would suggest that the sample of red crystals from which **8** was taken consisted of both **6** and **8**. Consistent with this, the solution of the red crystals in CDCl_3_, left to stand for some time, deposited a few red crystals that proved on X-ray examination to be compound **6**.

## 6. Refinement

Details of the measurements and refinements are given in Table 20 ▸. For all structures, multi-scan absorption corrections were applied using spherical harmonics, as implemented in the SCALE3 ABSPACK scaling algorithm (Rigaku OD, 2020 ▸). For compound **6**, analytical numeric absorption corrections using a face-indexed crystal model, based on expressions derived by Clark & Reid (1995 ▸), were applied first.

Aromatic hydrogen atoms were included at calculated positions and refined using a riding model with C—H = 0.95 Å. Methyl groups were included as idealized rigid groups with C—H = 0.98 Å and H—C—H = 109.5°, and were allowed to rotate but not tip (command ‘AFIX 137’). *U* values of the hydrogen atoms were fixed at 1.5 × *U*_eq_ of the parent carbon atoms for methyl groups and 1.2 × *U*_eq_ of the parent carbon atoms for other hydrogens. A small number of badly fitting reflections were omitted (**1a**, two reflections with deviations > 8*σ*; **1b**, seven reflections > 7*σ*; **2**, one reflection > 15*σ*; **5**, one reflection > 6*σ*; **8**, one reflection > 29*σ*).

Four of the crystals (**1a**, **1b**, **2** and **8**) were non-merohedral twins, with twinning by 180° rotation about the *a* axis for **1a**, **1b** and **2** and about [1
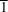
1] for **8**. These structures were refined using the ‘HKLF 5’ method (Sheldrick, 2015 ▸). The relative volumes of the smaller twinning components refined to 0.4710 (6), 0.4583 (6), 0.4641 (5) and 0.4440 (5), respectively. The twin data reduction merges equivalent reflections before writing the intensity file, so that *R*_int_ is meaningless (and is not given in Table 20 ▸). The intensity datasets comprise all non-overlapped reflections from both components and all overlapped reflections, so that the number of reflections should be inter­preted with caution. More stringent checks during the data reduction of twins (*e.g.* the command ‘remove outliers’) mean that the completeness of some datasets is less than ideal, typically around 95%.

*Special features and exceptions*: For **1b**, the large difference peak of 4.5 e Å^−3^ has coordinates that are arithmetically related to those of the gold atom and thus may represent residual twinning errors. For **3**, the *x* and *y* coordinates of the gold atom are *ca* 0.25, which leads to systematically weak reflection classes; *checkCIF* comments on (pseudo-) *B*-centring. The second weighting parameter *b* (Sheldrick, 2015 ▸) does not converge, but oscillates over a small range. For **6**, the methyl hydrogen atoms at C18 were unclear, and were therefore refined as an ideal hexa­gon of half-occupied sites (command ‘AFIX 127’). However, the disorder may be more extensive than this simple model.

## Supplementary Material

Crystal structure: contains datablock(s) 1a, 1b, 2, 3, 4, 5, 6, 7, 8, global. DOI: 10.1107/S2056989024007266/wm5729sup1.cif

Structure factors: contains datablock(s) 1a. DOI: 10.1107/S2056989024007266/wm57291asup2.hkl

Structure factors: contains datablock(s) 1b. DOI: 10.1107/S2056989024007266/wm57291bsup3.hkl

Structure factors: contains datablock(s) 2. DOI: 10.1107/S2056989024007266/wm57292sup4.hkl

Structure factors: contains datablock(s) 3. DOI: 10.1107/S2056989024007266/wm57293sup5.hkl

Structure factors: contains datablock(s) 4. DOI: 10.1107/S2056989024007266/wm57294sup6.hkl

Structure factors: contains datablock(s) 5. DOI: 10.1107/S2056989024007266/wm57295sup7.hkl

Structure factors: contains datablock(s) 6. DOI: 10.1107/S2056989024007266/wm57296sup8.hkl

Structure factors: contains datablock(s) 7. DOI: 10.1107/S2056989024007266/wm57297sup9.hkl

Structure factors: contains datablock(s) 8. DOI: 10.1107/S2056989024007266/wm57298sup10.hkl

CCDC references: 2145206, 2145207, 2145208, 2145212, 2145216, 2145218, 2145221, 2145222, 2145223

Additional supporting information:  crystallographic information; 3D view; checkCIF report

## Figures and Tables

**Figure 2:**
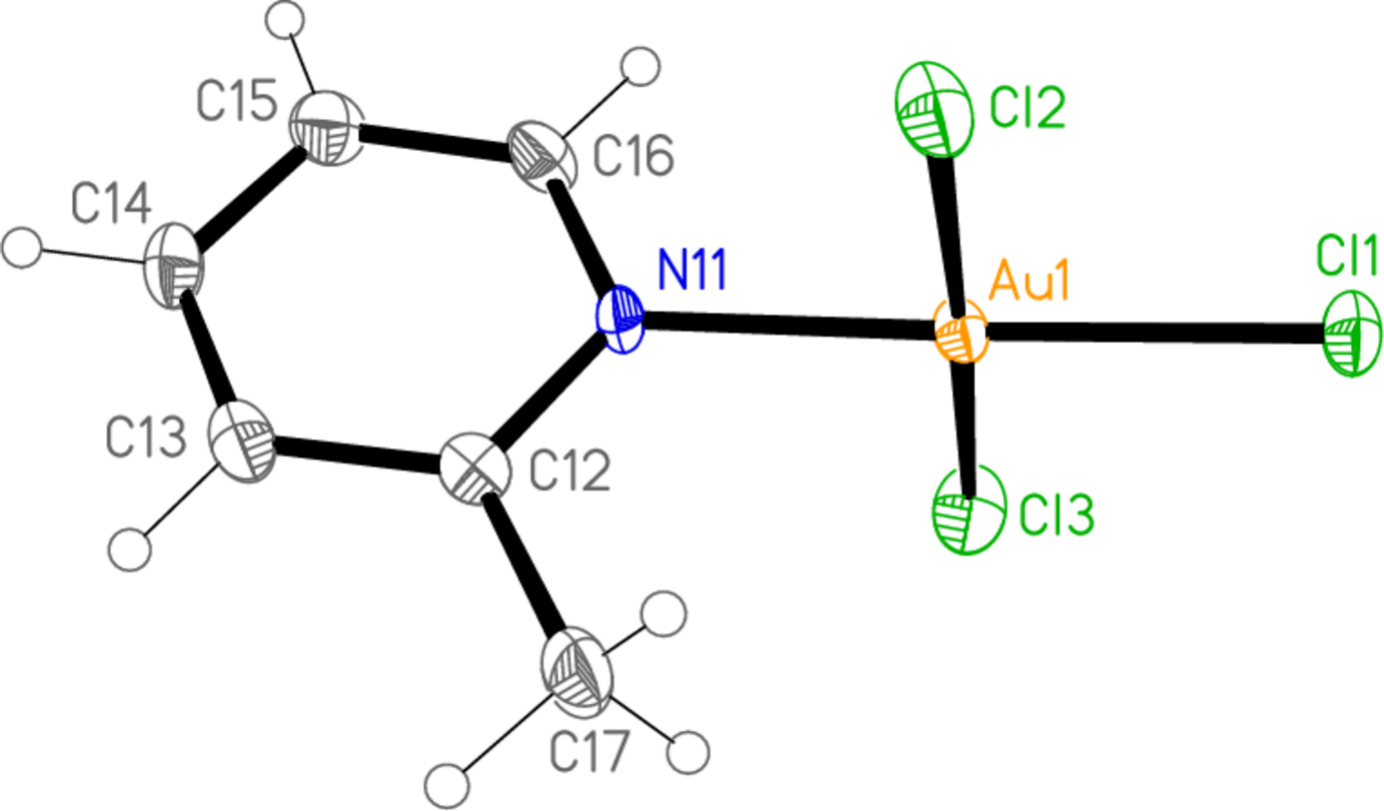
The mol­ecular structure of compound **1** (polymorph **1b**) in the crystal.

**Figure 3:**
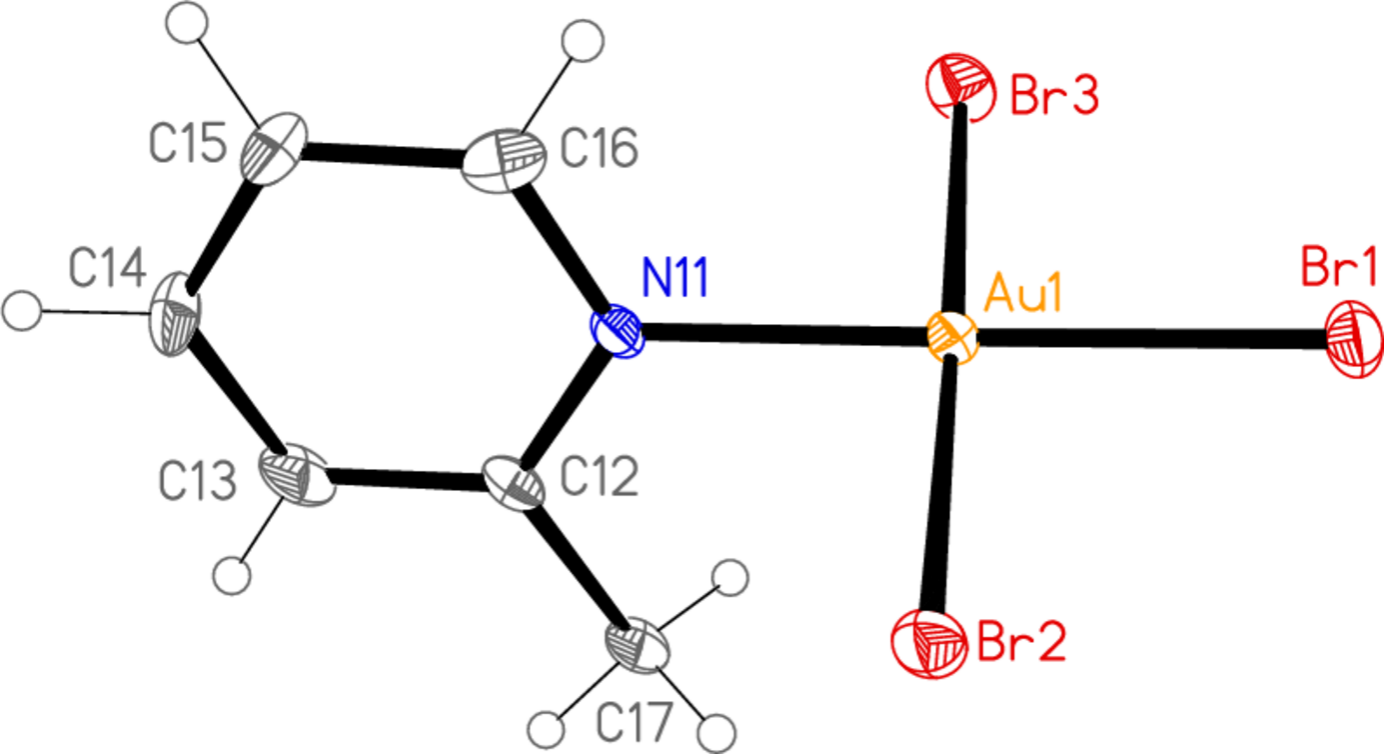
The mol­ecular structure of compound **2** in the crystal.

**Figure 4:**
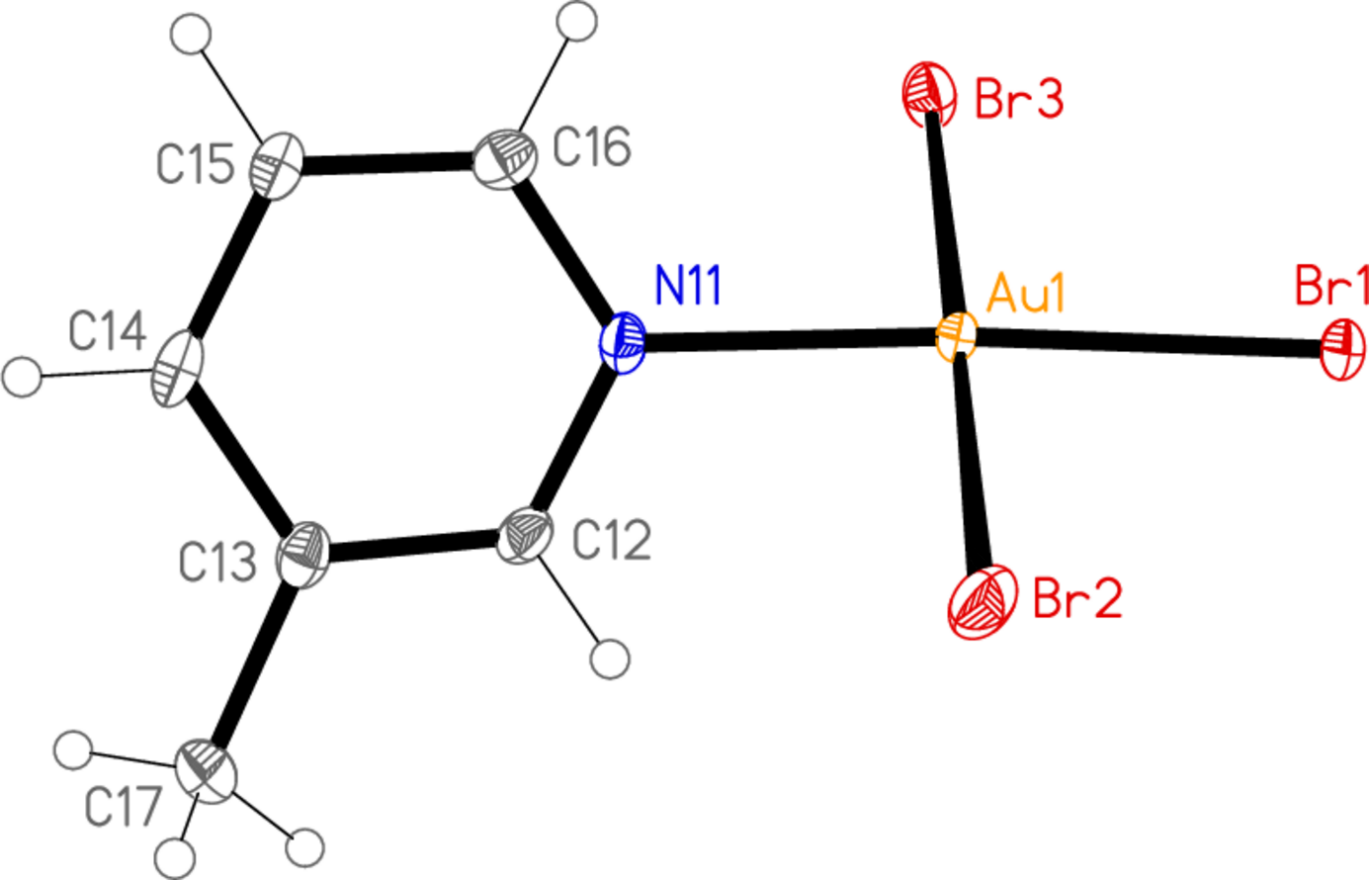
The mol­ecular structure of compound **3** in the crystal.

**Figure 5:**
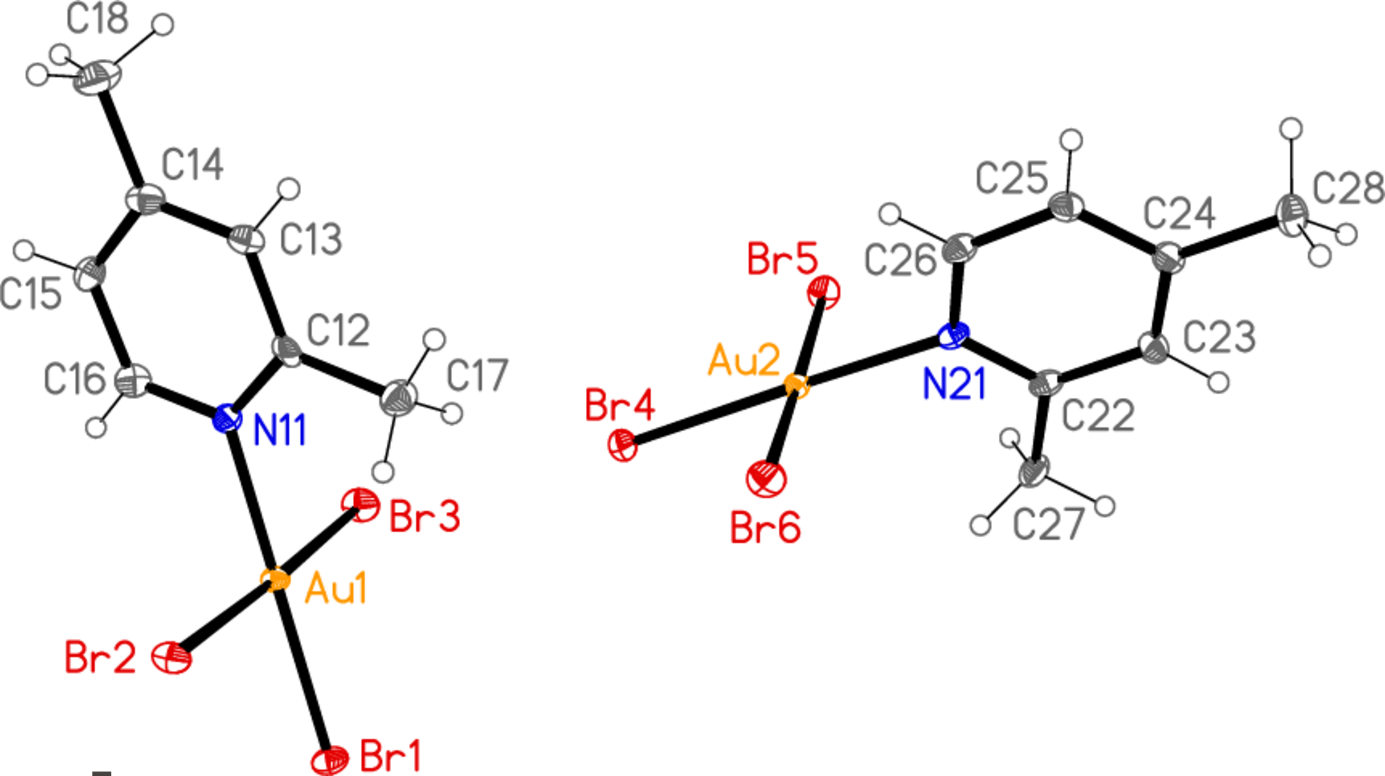
The mol­ecular structure of compound **4** (with two independent mol­ecules) in the crystal.

**Figure 6:**
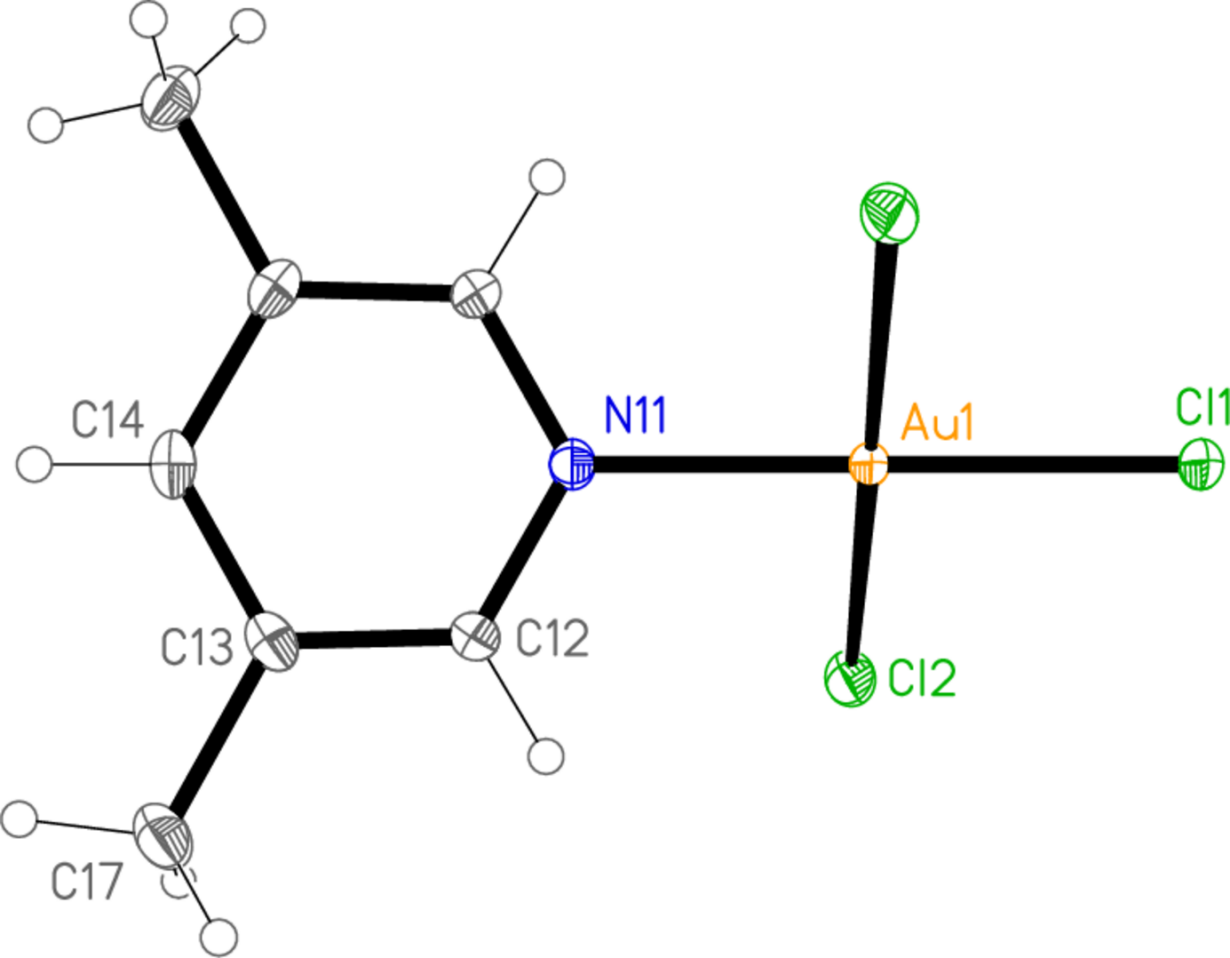
The mol­ecular structure of compound **5** in the crystal. Only the asymmetric unit is numbered.

**Figure 7:**
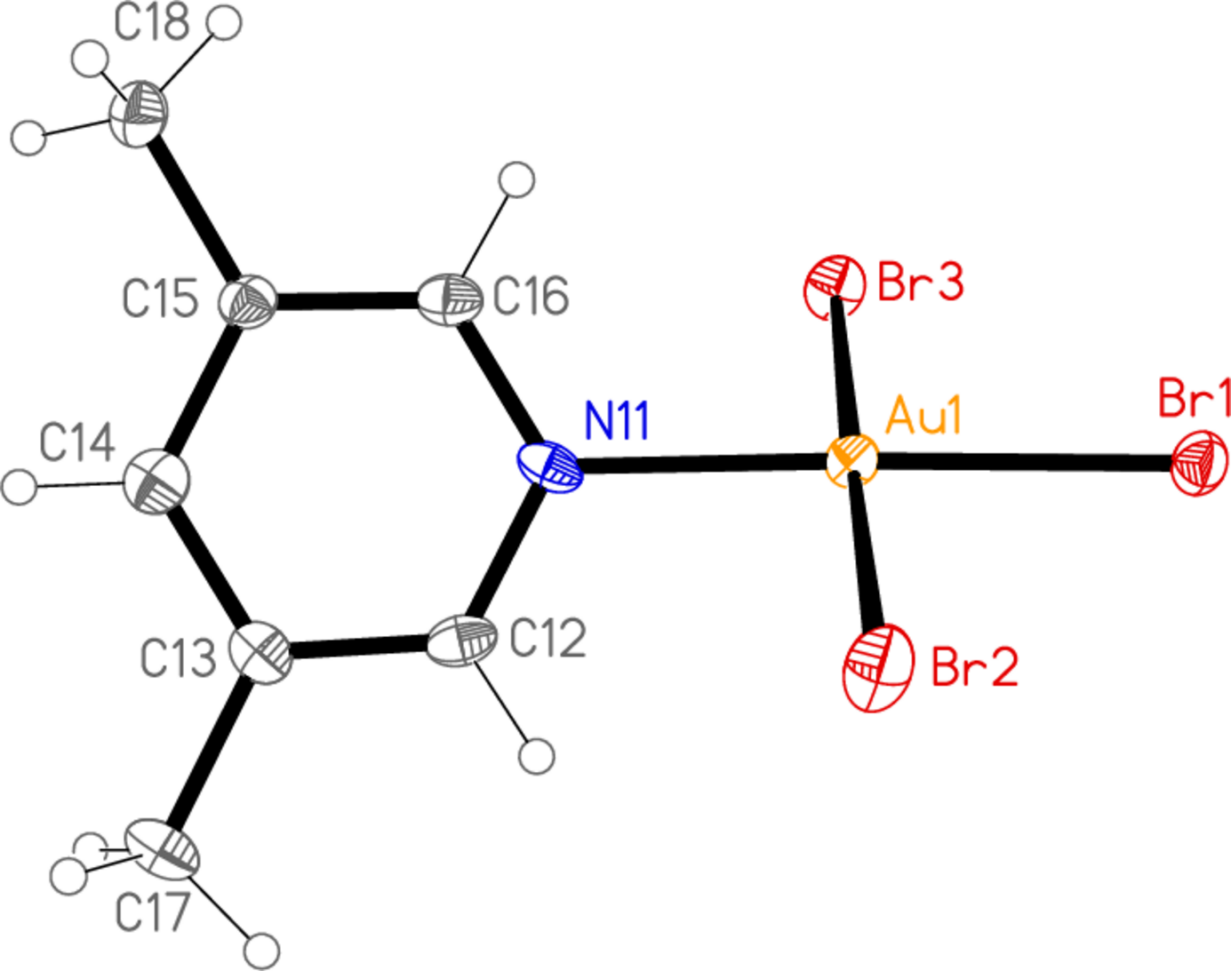
The mol­ecular structure of compound **6** in the crystal.

**Figure 8:**
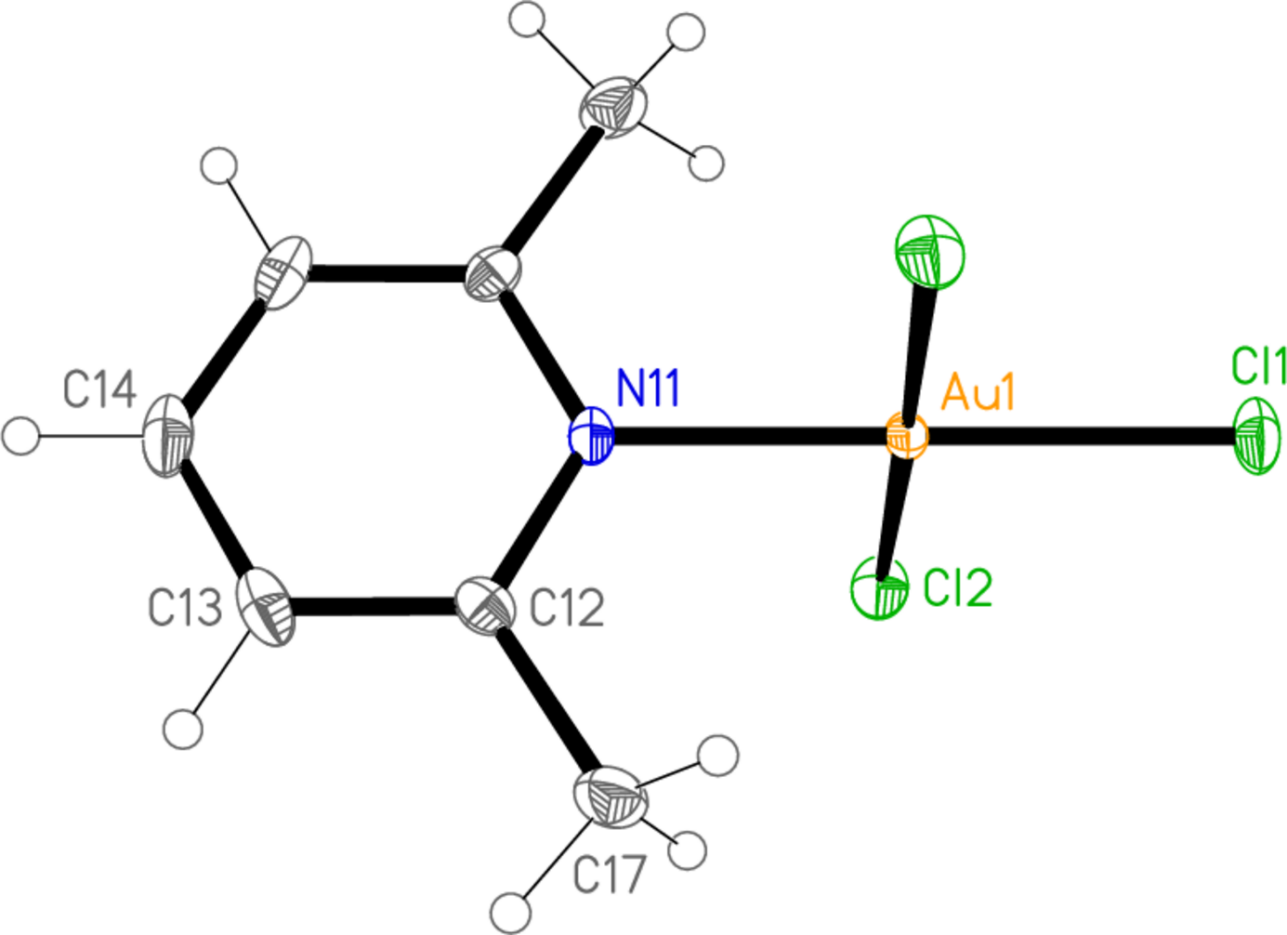
The mol­ecular structure of compound **7** in the crystal. Only the asymmetric unit is numbered.

**Figure 9:**
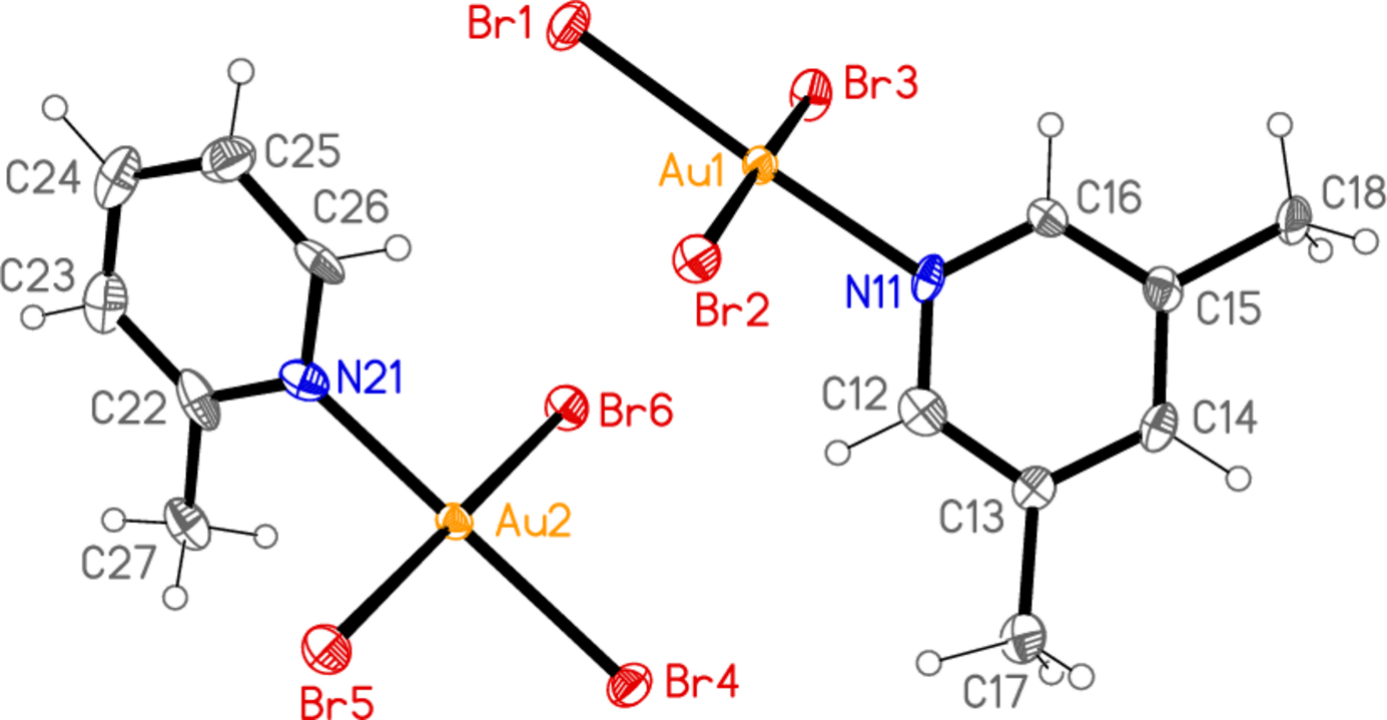
The mol­ecular structures of compound **8** (an adduct of **2** and **6**) in the crystal.

**Table 1:** Selected geometric parameters (Å, °) for **1a**[Chem scheme1]

Au1—N11	2.039 (4)	Au1—Cl2	2.2799 (15)
Au1—Cl3	2.2632 (15)	N11—C12	1.333 (7)
Au1—Cl1	2.2708 (14)	N11—C16	1.364 (7)
			
N11—Au1—Cl3	89.27 (13)	Cl3—Au1—Cl2	177.20 (5)
N11—Au1—Cl1	178.94 (13)	Cl1—Au1—Cl2	90.91 (5)
Cl3—Au1—Cl1	91.06 (6)	C12—N11—C16	121.2 (5)
N11—Au1—Cl2	88.81 (13)		
			
Cl3—Au1—N11—C12	101.8 (4)	Cl3—Au1—N11—C16	−80.4 (4)
Cl2—Au1—N11—C12	−76.2 (4)	Cl2—Au1—N11—C16	101.6 (4)

**Table 2:** Selected geometric parameters (Å, °) for **1b**[Chem scheme1]

Au1—N11	2.030 (4)	Au1—Cl2	2.2766 (12)
Au1—Cl3	2.2652 (14)	N11—C16	1.347 (6)
Au1—Cl1	2.2688 (13)	N11—C12	1.354 (7)
			
N11—Au1—Cl3	89.65 (14)	Cl3—Au1—Cl2	177.79 (5)
N11—Au1—Cl1	178.32 (13)	Cl1—Au1—Cl2	90.90 (5)
Cl3—Au1—Cl1	91.17 (5)	C16—N11—C12	120.1 (5)
N11—Au1—Cl2	88.27 (13)		
			
Cl3—Au1—N11—C16	−86.0 (4)	Cl3—Au1—N11—C12	98.2 (4)
Cl2—Au1—N11—C16	93.3 (4)	Cl2—Au1—N11—C12	−82.5 (4)

**Table 3:** Selected geometric parameters (Å, °) for **2**[Chem scheme1]

Au1—N11	2.050 (5)	Au1—Br2	2.4220 (8)
Au1—Br1	2.3996 (7)	N11—C16	1.349 (7)
Au1—Br3	2.4085 (8)	N11—C12	1.359 (8)
			
N11—Au1—Br1	178.93 (15)	Br1—Au1—Br2	90.88 (3)
N11—Au1—Br3	89.24 (16)	Br3—Au1—Br2	177.16 (3)
Br1—Au1—Br3	90.84 (3)	C16—N11—C12	120.3 (6)
N11—Au1—Br2	89.08 (16)		
			
Br3—Au1—N11—C16	−80.7 (5)	Br3—Au1—N11—C12	101.2 (5)
Br2—Au1—N11—C16	101.6 (5)	Br2—Au1—N11—C12	−76.5 (5)

**Table 4:** Selected geometric parameters (Å, °) for **3**[Chem scheme1]

Au1—N11	2.062 (4)	Au1—Br3	2.4276 (5)
Au1—Br1	2.4009 (5)	N11—C16	1.341 (6)
Au1—Br2	2.4225 (5)	N11—C12	1.346 (6)
			
N11—Au1—Br1	176.62 (10)	Br1—Au1—Br3	90.653 (17)
N11—Au1—Br2	88.25 (10)	Br2—Au1—Br3	176.494 (17)
Br1—Au1—Br2	90.649 (17)	C16—N11—C12	120.3 (4)
N11—Au1—Br3	90.63 (10)		
			
Br2—Au1—N11—C16	120.5 (3)	Br2—Au1—N11—C12	−58.4 (3)
Br3—Au1—N11—C16	−56.1 (3)	Br3—Au1—N11—C12	124.9 (3)

**Table 5:** Selected geometric parameters (Å, °) for **4**[Chem scheme1]

Au1—N11	2.062 (3)	Au2—N21	2.054 (4)
Au1—Br1	2.3998 (4)	Au2—Br4	2.3963 (4)
Au1—Br3	2.4070 (5)	Au2—Br6	2.4187 (5)
Au1—Br2	2.4235 (4)	Au2—Br5	2.4266 (5)
N11—C16	1.336 (5)	N21—C22	1.346 (5)
N11—C12	1.350 (5)	N21—C26	1.348 (5)
			
N11—Au1—Br1	179.86 (11)	N21—Au2—Br4	178.91 (10)
N11—Au1—Br3	88.97 (10)	N21—Au2—Br6	88.46 (10)
Br1—Au1—Br3	90.901 (16)	Br4—Au2—Br6	90.810 (16)
N11—Au1—Br2	89.16 (10)	N21—Au2—Br5	90.10 (10)
Br1—Au1—Br2	90.974 (15)	Br4—Au2—Br5	90.672 (16)
Br3—Au1—Br2	177.518 (17)	Br6—Au2—Br5	176.590 (17)
C16—N11—C12	120.5 (4)	C22—N21—C26	120.8 (4)
			
Br3—Au1—N11—C16	84.7 (3)	Br6—Au2—N21—C22	106.4 (3)
Br2—Au1—N11—C16	−93.7 (3)	Br5—Au2—N21—C22	−76.7 (3)
Br3—Au1—N11—C12	−97.3 (3)	Br6—Au2—N21—C26	−72.5 (3)
Br2—Au1—N11—C12	84.3 (3)	Br5—Au2—N21—C26	104.4 (3)

**Table 6:** Selected geometric parameters (Å, °) for **5**[Chem scheme1]

Au1—N11	2.037 (3)	Au1—Cl2	2.2867 (6)
Au1—Cl1	2.2716 (8)	N11—C12	1.352 (3)
			
N11—Au1—Cl1	180.0	Cl2—Au1—Cl2^i^	179.56 (3)
N11—Au1—Cl2	89.779 (14)	C12—N11—C12^i^	121.0 (3)
Cl1—Au1—Cl2	90.221 (14)		
			
Cl2—Au1—N11—C12	51.15 (11)	Cl2—Au1—N11—C12^i^	−128.85 (11)

Symmetry code: (i) 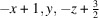.

**Table 7:** Selected geometric parameters (Å, °) for **6**[Chem scheme1]

Au1—N11	2.078 (4)	Au1—Br3	2.4295 (4)
Au1—Br1	2.3892 (5)	N11—C16	1.344 (6)
Au1—Br2	2.4167 (5)		
			
N11—Au1—Br1	178.39 (10)	Br1—Au1—Br3	89.674 (17)
N11—Au1—Br2	90.03 (10)	Br2—Au1—Br3	179.045 (17)
Br1—Au1—Br2	89.437 (17)	C16—N11—C12	121.8 (4)
N11—Au1—Br3	90.85 (10)		
			
Br2—Au1—N11—C16	124.0 (3)	Br2—Au1—N11—C12	−55.8 (3)
Br3—Au1—N11—C16	−56.3 (3)	Br3—Au1—N11—C12	123.9 (3)

**Table 8:** Selected geometric parameters (Å, °) for **7**[Chem scheme1]

Au1—N11	2.036 (2)	Au1—Cl2	2.2811 (5)
Au1—Cl1	2.2648 (7)	N11—C12	1.360 (2)
			
N11—Au1—Cl1	180.0	Cl2—Au1—Cl2^i^	178.75 (2)
N11—Au1—Cl2	89.375 (12)	C12^i^—N11—C12	121.7 (2)
Cl1—Au1—Cl2	90.625 (12)		
			
Cl2—Au1—N11—C12^i^	−92.76 (10)	Cl2—Au1—N11—C12	87.24 (10)

Symmetry code: (i) 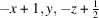.

**Table 9:** Selected geometric parameters (Å, °) for **8**[Chem scheme1]

Au1—N11	2.053 (8)	Au2—Br5	2.4136 (9)
Au1—Br1	2.3922 (11)	Au2—Br6	2.4253 (10)
Au1—Br3	2.4162 (9)	N11—C12	1.347 (11)
Au1—Br2	2.4306 (10)	N11—C16	1.360 (10)
Au2—N21	2.056 (8)	N21—C26	1.348 (11)
Au2—Br4	2.3901 (11)	N21—C22	1.360 (11)
			
N11—Au1—Br1	177.6 (2)	N21—Au2—Br5	89.90 (19)
N11—Au1—Br3	90.3 (2)	Br4—Au2—Br5	90.14 (4)
Br1—Au1—Br3	90.02 (4)	N21—Au2—Br6	89.95 (19)
N11—Au1—Br2	89.0 (2)	Br4—Au2—Br6	90.01 (4)
Br1—Au1—Br2	90.84 (4)	Br5—Au2—Br6	179.59 (4)
Br3—Au1—Br2	176.66 (4)	C12—N11—C16	120.2 (9)
N21—Au2—Br4	179.8 (2)	C26—N21—C22	120.6 (9)
			
Br3—Au1—N11—C12	−121.6 (6)	Br5—Au2—N21—C26	96.6 (6)
Br2—Au1—N11—C12	55.2 (6)	Br6—Au2—N21—C26	−83.8 (6)
Br3—Au1—N11—C16	60.5 (6)	Br5—Au2—N21—C22	−86.0 (7)
Br2—Au1—N11—C16	−122.7 (6)	Br6—Au2—N21—C22	93.7 (7)

**Table 10:** Hydrogen-bond geometry (Å, °) for **1a**[Chem scheme1]

*D*—H⋯*A*	*D*—H	H⋯*A*	*D*⋯*A*	*D*—H⋯*A*
C17—H17*B*⋯Cl1^i^	0.98	2.96	3.888 (6)	159
C14—H14⋯Cl2^ii^	0.95	2.93	3.538 (6)	123
C16—H16⋯Cl2^iii^	0.95	2.75	3.553 (6)	142
C17—H17*C*⋯Cl2	0.98	2.90	3.596 (6)	129

Symmetry codes: (i) 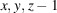; (ii) 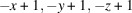; (iii) 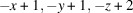.

**Table 11:** Hydrogen-bond geometry (Å, °) for **1b**[Chem scheme1]

*D*—H⋯*A*	*D*—H	H⋯*A*	*D*⋯*A*	*D*—H⋯*A*
C13—H13⋯Cl1^i^	0.95	2.79	3.683 (6)	157
C15—H15⋯Cl1^ii^	0.95	2.88	3.527 (6)	126
C17—H17*C*⋯Cl1^i^	0.98	2.86	3.798 (6)	160
C16—H16⋯Cl2^iii^	0.95	2.70	3.610 (6)	162

Symmetry codes: (i) 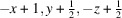; (ii) 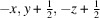; (iii) 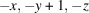.

**Table 12:** Hydrogen-bond geometry (Å, °) for **2**[Chem scheme1]

*D*—H⋯*A*	*D*—H	H⋯*A*	*D*⋯*A*	*D*—H⋯*A*
C14—H14⋯Br1^i^	0.95	3.09	3.754 (7)	129
C17—H17*A*⋯Br1^ii^	0.98	3.05	3.972 (7)	156
C17—H17*B*⋯Br1^iii^	0.98	3.06	3.982 (6)	157
C14—H14⋯Br2^iv^	0.95	3.04	3.636 (7)	122
C16—H16⋯Br2^v^	0.95	2.86	3.683 (7)	146
C17—H17*C*⋯Br2	0.98	2.96	3.690 (7)	132

Symmetry codes: (i) 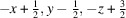; (ii) 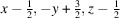; (iii) 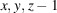; (iv) 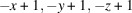; (v) 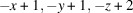.

**Table 13:** Hydrogen-bond geometry (Å, °) for **3**[Chem scheme1]

*D*—H⋯*A*	*D*—H	H⋯*A*	*D*⋯*A*	*D*—H⋯*A*
C15—H15⋯Br1^i^	0.95	2.97	3.905 (5)	168
C16—H16⋯Br1^ii^	0.95	2.86	3.721 (5)	151
C12—H12⋯Br2^iii^	0.95	2.88	3.684 (4)	144
C14—H14⋯Br3^iv^	0.95	3.08	3.880 (5)	143
C17—H17*B*⋯Br3^iv^	0.98	3.06	3.984 (5)	159

Symmetry codes: (i) 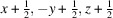; (ii) 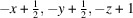; (iii) 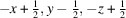; (iv) 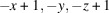.

**Table 14:** Hydrogen-bond geometry (Å, °) for **4**[Chem scheme1]

*D*—H⋯*A*	*D*—H	H⋯*A*	*D*⋯*A*	*D*—H⋯*A*
C16—H16⋯Br2^i^	0.95	2.86	3.755 (5)	158
C17—H17*C*⋯Br4	0.98	2.89	3.861 (5)	171
C23—H23⋯Br4^ii^	0.95	2.95	3.765 (4)	145
C25—H25⋯Br2^iii^	0.95	2.92	3.851 (4)	166
C26—H26⋯Br5^iv^	0.95	2.98	3.890 (4)	161
C27—H27*B*⋯Br3^ii^	0.98	3.07	3.855 (4)	138
C27—H27*C*⋯Br5	0.98	3.01	3.647 (4)	124

Symmetry codes: (i) 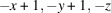; (ii) 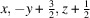; (iii) 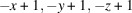; (iv) 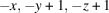.

**Table 15:** Hydrogen-bond geometry (Å, °) for **5**[Chem scheme1]

*D*—H⋯*A*	*D*—H	H⋯*A*	*D*⋯*A*	*D*—H⋯*A*
C12—H12⋯Cl1^ii^	0.95	2.67	3.564 (2)	158
C14—H14⋯Cl2^iii^	0.95	2.87	3.659 (3)	142
C14—H14⋯Cl2^iv^	0.95	2.87	3.659 (3)	142

Symmetry codes: (ii) 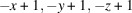; (iii) 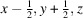; (iv) 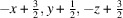.

**Table 16:** Hydrogen-bond geometry (Å, °) for **6**[Chem scheme1]

*D*—H⋯*A*	*D*—H	H⋯*A*	*D*⋯*A*	*D*—H⋯*A*
C16—H16⋯Br1^i^	0.95	2.91	3.792 (4)	154
C17—H17*C*⋯Br1^ii^	0.98	2.87	3.749 (5)	149
C18—H18*A*⋯Br1^i^	0.98	2.89	3.784 (5)	151
C18—H18*C*⋯Br3^iii^	0.98	2.93	3.902 (5)	174
C14—H14⋯Br2^iv^	0.95	3.02	3.826 (5)	144

Symmetry codes: (i) 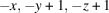; (ii) 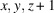; (iii) 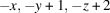; (iv) 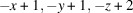.

**Table 17:** Hydrogen-bond geometry (Å, °) for **7**[Chem scheme1]

*D*—H⋯*A*	*D*—H	H⋯*A*	*D*⋯*A*	*D*—H⋯*A*
C17—H17*C*⋯Cl1^ii^	0.98	2.87	3.701 (2)	144
C14—H14⋯Cl1^iii^	0.95	2.67	3.621 (3)	180

Symmetry codes: (ii) 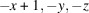; (iii) 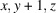.

**Table 18:** Hydrogen-bond geometry (Å, °) for **8**[Chem scheme1]

*D*—H⋯*A*	*D*—H	H⋯*A*	*D*⋯*A*	*D*—H⋯*A*
C18—H18*C*⋯Br3^i^	0.98	3.05	3.892 (10)	145
C23—H23⋯Br4^ii^	0.95	3.01	3.815 (10)	143
C17—H17*C*⋯Br6^i^	0.98	2.90	3.792 (10)	152
C16—H16⋯Br5^iii^	0.95	3.06	4.007 (9)	172
C25—H25⋯Br1^iv^	0.95	3.06	3.721 (10)	128
C26—H26⋯Br2	0.95	2.94	3.835 (10)	159

Symmetry codes: (i) 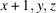; (ii) 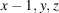; (iii) 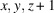; (iv) 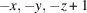.

**Table 19:** Summary of packing features

Compound	No. of axial Au⋯*X* contacts	Offset-stacked dimers [(Au—*X*)_2_ quadrilaterals]	Connected to form ⋯*^*b*^*	Further linkages*^*b*^*
(py)AuCl (Adams & Strähle, 1982 ▸)	2	yes	double chain (ladder) *via* edge-linked quadrilaterals	
**1a** and **2** (isotypic)	1	yes	double chain *via X*⋯*X* contacts	
**1b**	0	no	double chain *via* Cl⋯Cl and H⋯Cl contacts	
**3**	2	yes	layer *via* Br⋯Br contacts	layers connected *via* Br⋯Br contacts
**4** (both mol­ecules)	1	yes	double chain *via* Br⋯Br contacts (analogous to **1a** and **2**)	layers connected *via* Br⋯Br contacts
**5**	2	yes	chain *via* apex-linked quadrilaterals	connected to form layers *via* Cl⋯Cl contacts
**6**	1	yes	double chain *via* Br⋯Br contacts (analogous to **1a** and **2**)	connected to form double layer *via* Br⋯Br contacts
**7**	0	no	layer *via* H⋯Cl and Cl⋯π contacts	
**8**	Au1 2, Au2 0	Au1 yes, Au2 no	layer *via* Br⋯Br contacts	layers connected *via* Br⋯Br contacts
ESITIM (Hobbollahi *et al.*, 2019 ▸)*^*a*^*	2	no	layer structure with linked tetra­meric rings	
WEFQAD (Pizzi *et al.*, 2022 ▸)*^*a*^*	2	yes	ladder structure	ladders connected *via* Br⋯Cl contacts
WEFQEH (Pizzi *et al.*, 2022 ▸)*^*a*^*	2	no	layer structure analogous to ESITIM	layers connected *via* F⋯F contacts
WEFQIL (Pizzi *et al.*, 2022 ▸)*^*a*^*	2	yes	chain *via* apex-linked quadrilaterals	connected to form layers *via* Cl⋯Cl_py_ contacts
WEFQOR (Pizzi *et al.*, 2022 ▸)*^*a*^*	2	yes	ladder structure	connected to form layers *via* I⋯Cl contacts.
WEFRAE (Pizzi *et al.*, 2022 ▸)*^*a*^*	2	yes	chain *via* apex-linked quadrilaterals	connected to form layers *via* Br⋯Br contacts

Notes: (*a*) Refcodes refer to structures whose packing is discussed in the section *Database survey*; (*b*) these columns do not necessarily present an exhaustive list; see text for further details.

**Table:** 

	**1a**	**1b**	**2**	**3**	**4**
Crystal data					
Chemical formula	[AuCl_3_(C_6_H_7_N)]	[AuCl_3_(C_6_H_7_N)]	[AuBr_3_(C_6_H_7_N)]	[AuBr_3_(C_6_H_7_N)]	[AuBr_3_(C_7_H_9_N)]
*M*_r_	396.44	396.44	529.82	529.82	543.85
Crystal system, space group	Monoclinic, *P*2_1_/*n*	Monoclinic, *P*2_1_/*c*	Monoclinic, *P*2_1_/*n*	Monoclinic, *C*2/*c*	Monoclinic, *P*2_1_/*c*
Temperature (K)	100	100	100	100	100
*a*, *b*, *c* (Å)	7.6852 (7), 14.3992 (12), 8.7963 (7)	7.8077 (8), 16.4881 (15), 7.6929 (9)	8.0776 (4), 14.7230 (7), 8.9682 (4)	18.6763 (6), 6.78409 (14), 18.4807 (5)	8.07477 (17), 17.2853 (4), 16.3527 (4)
α, β, γ (°)	90, 95.860 (8), 90	90, 103.920 (12), 90	90, 96.617 (5), 90	90, 118.887 (4), 90	90, 90.818 (2), 90
*V* (Å^3^)	968.32 (14)	961.26 (18)	1059.45 (9)	2050.18 (12)	2282.19 (8)
*Z*	4	4	4	8	8
Radiation type	Mo *K*α	Mo *K*α	Mo *K*α	Mo *K*α	Mo *K*α
μ (mm^−1^)	15.96	16.07	25.14	25.99	23.35
Crystal size (mm)	0.30 × 0.03 × 0.02	0.22 × 0.10 × 0.02	0.07 × 0.07 × 0.07	0.12 × 0.10 × 0.02	0.10 × 0.10 × 0.02

Data collection					
Diffractometer	Oxford Diffraction Xcalibur, Eos	Oxford Diffraction Xcalibur, Eos	Oxford Diffraction Xcalibur, Eos	Oxford Diffraction Xcalibur, Eos	Oxford Diffraction Xcalibur, Eos
Absorption correction	Multi-scan (*CrysAlis PRO*; Rigaku OD, 2020 ▸)	Multi-scan (*CrysAlis PRO*; Rigaku OD, 2020 ▸)	Multi-scan (*CrysAlis PRO*; Rigaku OD, 2020 ▸)	Multi-scan (*CrysAlis PRO*; Rigaku OD, 2020 ▸)	Multi-scan (*CrysAlis PRO*; Rigaku OD, 2020 ▸)
*T*_min_, *T*_max_	0.333, 1.000	0.254, 1.000	0.571, 1.000	0.309, 1.000	0.338, 1.000
No. of measured, independent and observed [*I* > 2σ(*I*)] reflections	3572, 3572, 2943	3603, 3603, 2910	3597, 3597, 2716	40540, 3120, 2563	139727, 6677, 5614
*R*_int_	–	–	–	0.064	0.086
θ values (°)	θ_max_ = 28.3, θ_min_ = 3.4	θ_max_ = 28.3, θ_min_ = 3.0	θ_max_ = 28.3, θ_min_ = 3.2	θ_max_ = 31.0, θ_min_ = 2.5	θ_max_ = 30.0, θ_min_ = 2.4
(sin θ/λ)_max_ (Å^−1^)	0.667	0.667	0.667	0.724	0.704

Refinement					
*R*[*F*^2^ > 2σ(*F*^2^)], *wR*(*F*^2^), *S*	0.024, 0.049, 0.93	0.025, 0.050, 1.00	0.028, 0.040, 0.80	0.026, 0.054, 1.04	0.026, 0.048, 1.05
No. of reflections	3572	3603	3597	3120	6677
No. of parameters	102	102	102	101	222
H-atom treatment	H-atom parameters constrained	H-atom parameters constrained	H-atom parameters constrained	H-atom parameters constrained	H-atom parameters constrained
Δρ_max_, Δρ_min_ (e Å^−3^)	1.86, −0.87	4.52, −0.87	1.26, −1.40	2.01, −1.71	1.39, −1.59

**Table:** 

	**5**	**6**	**7**	**8**
Crystal data				
Chemical formula	[AuCl_3_(C_7_H_9_N)]	[AuBr_3_(C_7_H_9_N)]	[AuCl_3_(C_7_H_9_N)]	[AuBr_3_(C_7_H_9_N)]·[AuBr_3_(C_6_H_7_N)]
*M*_r_	410.47	543.85	410.47	1073.67
Crystal system, space group	Monoclinic, *C*2/*c*	Triclinic, *P* 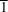	Monoclinic, *C*2/*c*	Triclinic, *P* 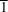
Temperature (K)	100	100	100	100
*a*, *b*, *c* (Å)	7.6240 (3), 15.9360 (6), 9.2538 (4)	8.2506 (3), 8.4726 (4), 9.4210 (4)	11.0184 (3), 10.6600 (2), 9.7760 (3)	9.1741 (9), 11.1922 (9), 11.4596 (7)
α, β, γ (°)	90, 112.333 (5), 90	113.828 (4), 103.543 (4), 98.368 (4)	90, 113.053 (3), 90	83.990 (6), 80.777 (6), 69.147 (8)
*V* (Å^3^)	1039.97 (8)	563.93 (5)	1056.55 (5)	1083.92 (16)
*Z*	4	2	4	2
Radiation type	Mo *K*α	Mo *K*α	Mo *K*α	Mo *K*α
μ (mm^−1^)	14.86	23.62	14.63	24.58
Crystal size (mm)	0.20 × 0.12 × 0.04	0.21 × 0.15 × 0.02	0.22 × 0.20 × 0.12	0.13 × 0.06 × 0.02

Data collection				
Diffractometer	Oxford Diffraction Xcalibur, Eos	Oxford Diffraction Xcalibur, Eos	Oxford Diffraction Xcalibur, Eos	Oxford Diffraction Xcalibur, Eos
Absorption correction	Multi-scan (*CrysAlis PRO*; Rigaku OD, 2020 ▸)	Analytical (*CrysAlis PRO*; Rigaku OD, 2020 ▸)	Multi-scan (*CrysAlis PRO*; Rigaku OD, 2020 ▸)	Multi-scan (*CrysAlis PRO*; Rigaku OD, 2020 ▸)
*T*_min_, *T*_max_	0.270, 1.000	0.050, 0.683	0.267, 1.000	0.052, 1.000
No. of measured, independent and observed [*I* > 2σ(*I*)] reflections	13810, 1563, 1496	30785, 3392, 3017	15246, 1600, 1553	7479, 7479, 5015
*R*_int_	0.037	0.071	0.030	–
θ values (°)	θ_max_ = 30.9, θ_min_ = 2.6	θ_max_ = 31.1, θ_min_ = 2.5	θ_max_ = 31.1, θ_min_ = 2.8	θ_max_ = 28.3, θ_min_ = 2.4
(sin θ/λ)_max_ (Å^−1^)	0.722	0.727	0.726	0.667

Refinement				
*R*[*F*^2^ > 2σ(*F*^2^)], *wR*(*F*^2^), *S*	0.014, 0.032, 1.07	0.027, 0.059, 1.06	0.013, 0.026, 1.12	0.035, 0.055, 0.82
No. of reflections	1563	3392	1600	7479
No. of parameters	58	111	59	212
H-atom treatment	H-atom parameters constrained	H-atom parameters constrained	H-atom parameters constrained	H-atom parameters constrained
Δρ_max_, Δρ_min_ (e Å^−3^)	1.26, −0.88	1.61, −1.50	0.83, −1.17	2.16, −1.91

Computer programs: *CrysAlis PRO* (Rigaku OD, 2020 ▸), *SHELXS97* (Sheldrick, 2008 ▸), *SHELXL2019/3* (Sheldrick, 2015 ▸) and *XP* (Bruker, 1998 ▸).

## supplementary crystallographic information

### Trichlorido(2-methylpyridine-κN)gold(III) (1a) Crystal data

**Table:**

[AuCl_3_(C_6_H_7_N)]	*F*(000) = 720
*M**_r_* = 396.44	*D*_x_ = 2.719 Mg m^−^^3^
Monoclinic, *P*2_1_/*n*	Mo *K*α radiation, λ = 0.71073 Å
*a* = 7.6852 (7) Å	Cell parameters from 9050 reflections
*b* = 14.3992 (12) Å	θ = 3.0–29.9°
*c* = 8.7963 (7) Å	µ = 15.96 mm^−^^1^
β = 95.860 (8)°	*T* = 100 K
*V* = 968.32 (14) Å^3^	Needle, yellow
*Z* = 4	0.30 × 0.03 × 0.02 mm

### Trichlorido(2-methylpyridine-κN)gold(III) (1a) Data collection

**Table:**

Oxford Diffraction Xcalibur, Eos diffractometer	3572 independent reflections
Radiation source: Enhance (Mo) X-ray Source	2943 reflections with *I* > 2σ(*I*)
Graphite monochromator	*R*_int_ = –
Detector resolution: 16.1419 pixels mm^-1^	θ_max_ = 28.3°, θ_min_ = 3.4°
ω scans	*h* = −10→10
Absorption correction: multi-scan (CrysAlisPro; Rigaku OD, 2020)	*k* = −19→19
*T*_min_ = 0.333, *T*_max_ = 1.000	*l* = −11→11
3572 measured reflections	

### Trichlorido(2-methylpyridine-κN)gold(III) (1a) Refinement

**Table:**

Refinement on *F*^2^	Primary atom site location: structure-invariant direct methods
Least-squares matrix: full	Secondary atom site location: difference Fourier map
*R*[*F*^2^ > 2σ(*F*^2^)] = 0.024	Hydrogen site location: inferred from neighbouring sites
*wR*(*F*^2^) = 0.049	H-atom parameters constrained
*S* = 0.93	*w* = 1/[σ^2^(*F*_o_^2^) + (0.0243*P*)^2^] where *P* = (*F*_o_^2^ + 2*F*_c_^2^)/3
3572 reflections	(Δ/σ)_max_ = 0.001
102 parameters	Δρ_max_ = 1.86 e Å^−^^3^
0 restraints	Δρ_min_ = −0.86 e Å^−^^3^

### Trichlorido(2-methylpyridine-κN)gold(III) (1a) Fractional atomic coordinates and isotropic or equivalent isotropic displacement parameters (Å^2^)

**Table:**

	*x*	*y*	*z*	*U*_iso_*/*U*_eq_	
Au1	0.31798 (3)	0.60929 (2)	0.91424 (2)	0.01336 (6)	
Cl1	0.3814 (2)	0.68716 (10)	1.13752 (16)	0.0235 (3)	
Cl2	0.60819 (18)	0.59459 (10)	0.88550 (16)	0.0216 (3)	
Cl3	0.02767 (19)	0.62595 (10)	0.93062 (17)	0.0228 (3)	
N11	0.2625 (6)	0.5372 (3)	0.7158 (5)	0.0145 (10)	
C12	0.2733 (7)	0.5754 (4)	0.5791 (7)	0.0180 (13)	
C13	0.2423 (8)	0.5203 (4)	0.4490 (7)	0.0213 (13)	
H13	0.249795	0.546607	0.350865	0.026*	
C14	0.2010 (8)	0.4280 (4)	0.4621 (7)	0.0240 (14)	
H14	0.181597	0.390143	0.373554	0.029*	
C15	0.1879 (8)	0.3911 (4)	0.6045 (7)	0.0268 (14)	
H15	0.157140	0.327702	0.615089	0.032*	
C16	0.2193 (8)	0.4459 (4)	0.7300 (7)	0.0217 (14)	
H16	0.211063	0.420448	0.828621	0.026*	
C17	0.3146 (8)	0.6764 (4)	0.5693 (7)	0.0251 (15)	
H17A	0.228129	0.712469	0.618885	0.038*	
H17B	0.311091	0.694740	0.461793	0.038*	
H17C	0.431574	0.688223	0.620934	0.038*	

### Trichlorido(2-methylpyridine-κN)gold(III) (1a) Atomic displacement parameters (Å^2^)

**Table:**

	*U*^11^	*U*^22^	*U*^33^	*U*^12^	*U*^13^	*U*^23^
Au1	0.01501 (10)	0.01177 (10)	0.01354 (10)	−0.00077 (10)	0.00270 (8)	−0.00073 (11)
Cl1	0.0311 (9)	0.0223 (8)	0.0167 (7)	−0.0007 (6)	0.0013 (6)	−0.0037 (6)
Cl2	0.0161 (7)	0.0276 (9)	0.0207 (7)	−0.0037 (6)	0.0009 (6)	−0.0014 (6)
Cl3	0.0179 (7)	0.0270 (9)	0.0239 (8)	0.0025 (6)	0.0035 (6)	−0.0025 (7)
N11	0.015 (3)	0.017 (2)	0.012 (2)	0.0020 (19)	0.0009 (19)	0.0006 (19)
C12	0.015 (3)	0.014 (3)	0.024 (3)	−0.001 (2)	0.003 (3)	0.003 (2)
C13	0.023 (4)	0.030 (4)	0.011 (3)	0.001 (3)	0.002 (2)	−0.001 (3)
C14	0.030 (4)	0.025 (3)	0.017 (3)	−0.001 (3)	0.002 (3)	−0.005 (3)
C15	0.038 (4)	0.015 (3)	0.028 (4)	−0.001 (3)	0.003 (3)	0.002 (3)
C16	0.027 (4)	0.020 (3)	0.017 (3)	−0.003 (3)	−0.001 (3)	0.001 (3)
C17	0.039 (4)	0.022 (3)	0.014 (3)	0.000 (3)	0.001 (3)	0.002 (3)

### Trichlorido(2-methylpyridine-κN)gold(III) (1a) Geometric parameters (Å, º)

**Table:**

Au1—N11	2.039 (4)	C13—H13	0.9500
Au1—Cl3	2.2632 (15)	C14—C15	1.374 (8)
Au1—Cl1	2.2708 (14)	C14—H14	0.9500
Au1—Cl2	2.2799 (15)	C15—C16	1.359 (8)
N11—C12	1.333 (7)	C15—H15	0.9500
N11—C16	1.364 (7)	C16—H16	0.9500
C12—C13	1.393 (8)	C17—H17A	0.9800
C12—C17	1.493 (8)	C17—H17B	0.9800
C13—C14	1.373 (8)	C17—H17C	0.9800
			
N11—Au1—Cl3	89.27 (13)	C13—C14—C15	119.3 (6)
N11—Au1—Cl1	178.94 (13)	C13—C14—H14	120.3
Cl3—Au1—Cl1	91.06 (6)	C15—C14—H14	120.3
N11—Au1—Cl2	88.81 (13)	C16—C15—C14	119.5 (6)
Cl3—Au1—Cl2	177.20 (5)	C16—C15—H15	120.2
Cl1—Au1—Cl2	90.91 (5)	C14—C15—H15	120.2
C12—N11—C16	121.2 (5)	C15—C16—N11	120.7 (6)
C12—N11—Au1	122.4 (4)	C15—C16—H16	119.7
C16—N11—Au1	116.4 (4)	N11—C16—H16	119.7
N11—C12—C13	119.0 (5)	C12—C17—H17A	109.5
N11—C12—C17	119.2 (5)	C12—C17—H17B	109.5
C13—C12—C17	121.8 (5)	H17A—C17—H17B	109.5
C14—C13—C12	120.2 (6)	C12—C17—H17C	109.5
C14—C13—H13	119.9	H17A—C17—H17C	109.5
C12—C13—H13	119.9	H17B—C17—H17C	109.5
			
Cl3—Au1—N11—C12	101.8 (4)	N11—C12—C13—C14	0.1 (9)
Cl2—Au1—N11—C12	−76.2 (4)	C17—C12—C13—C14	−178.6 (6)
Cl3—Au1—N11—C16	−80.4 (4)	C12—C13—C14—C15	1.0 (9)
Cl2—Au1—N11—C16	101.6 (4)	C13—C14—C15—C16	−1.2 (9)
C16—N11—C12—C13	−1.0 (8)	C14—C15—C16—N11	0.4 (9)
Au1—N11—C12—C13	176.6 (4)	C12—N11—C16—C15	0.8 (9)
C16—N11—C12—C17	177.8 (5)	Au1—N11—C16—C15	−177.0 (4)
Au1—N11—C12—C17	−4.6 (7)		

### Trichlorido(2-methylpyridine-κN)gold(III) (1a) Hydrogen-bond geometry (Å, º)

**Table:**

*D*—H···*A*	*D*—H	H···*A*	*D*···*A*	*D*—H···*A*
C13—H13···Cl1^i^	0.95	3.00	3.876 (6)	153
C14—H14···Cl1^ii^	0.95	2.96	3.617 (6)	127
C15—H15···Cl1^ii^	0.95	3.00	3.634 (6)	126
C17—H17*A*···Cl1^iii^	0.98	3.05	3.964 (6)	156
C17—H17*B*···Cl1^i^	0.98	2.96	3.888 (6)	159
C14—H14···Cl2^iv^	0.95	2.93	3.538 (6)	123
C16—H16···Cl2^v^	0.95	2.75	3.553 (6)	142
C17—H17*C*···Cl2	0.98	2.90	3.596 (6)	129
C14—H14···Cl3^vi^	0.95	2.99	3.793 (6)	144

Symmetry codes: (i) *x*, *y*, *z*−1; (ii) −*x*+1/2, *y*−1/2, −*z*+3/2; (iii) *x*−1/2, −*y*+3/2, *z*−1/2; (iv) −*x*+1, −*y*+1, −*z*+1; (v) −*x*+1, −*y*+1, −*z*+2; (vi) −*x*, −*y*+1, −*z*+1.

### Trichlorido(2-methylpyridine-κN)gold(III) (1b) Crystal data

**Table:**

[AuCl_3_(C_6_H_7_N)]	*F*(000) = 720
*M**_r_* = 396.44	*D*_x_ = 2.739 Mg m^−^^3^
Monoclinic, *P*2_1_/*c*	Mo *K*α radiation, λ = 0.71073 Å
*a* = 7.8077 (8) Å	Cell parameters from 5189 reflections
*b* = 16.4881 (15) Å	θ = 3.0–29.8°
*c* = 7.6929 (9) Å	µ = 16.07 mm^−^^1^
β = 103.920 (12)°	*T* = 100 K
*V* = 961.26 (18) Å^3^	Plate, yellow
*Z* = 4	0.22 × 0.10 × 0.02 mm

### Trichlorido(2-methylpyridine-κN)gold(III) (1b) Data collection

**Table:**

Oxford Diffraction Xcalibur, Eos diffractometer	3603 independent reflections
Radiation source: Enhance (Mo) X-ray Source	2910 reflections with *I* > 2σ(*I*)
Graphite monochromator	*R*_int_ = –
Detector resolution: 16.1419 pixels mm^-1^	θ_max_ = 28.3°, θ_min_ = 3.0°
ω scan	*h* = −10→10
Absorption correction: multi-scan (CrysAlisPro; Rigaku OD, 2020)	*k* = −21→21
*T*_min_ = 0.254, *T*_max_ = 1.000	*l* = −10→10
3603 measured reflections	

### Trichlorido(2-methylpyridine-κN)gold(III) (1b) Refinement

**Table:**

Refinement on *F*^2^	Primary atom site location: structure-invariant direct methods
Least-squares matrix: full	Secondary atom site location: difference Fourier map
*R*[*F*^2^ > 2σ(*F*^2^)] = 0.025	Hydrogen site location: inferred from neighbouring sites
*wR*(*F*^2^) = 0.050	H-atom parameters constrained
*S* = 1.00	*w* = 1/[σ^2^(*F*_o_^2^) + (0.0258*P*)^2^] where *P* = (*F*_o_^2^ + 2*F*_c_^2^)/3
3603 reflections	(Δ/σ)_max_ = 0.001
102 parameters	Δρ_max_ = 4.52 e Å^−^^3^
0 restraints	Δρ_min_ = −0.87 e Å^−^^3^

### Trichlorido(2-methylpyridine-κN)gold(III) (1b) Fractional atomic coordinates and isotropic or equivalent isotropic displacement parameters (Å^2^)

**Table:**

	*x*	*y*	*z*	*U*_iso_*/*U*_eq_	
Au1	0.25056 (3)	0.51656 (2)	0.21354 (3)	0.01380 (6)	
Cl1	0.31201 (18)	0.38204 (8)	0.21459 (18)	0.0207 (3)	
Cl2	0.2469 (2)	0.52510 (9)	−0.08269 (17)	0.0223 (3)	
Cl3	0.2480 (2)	0.51253 (9)	0.50724 (19)	0.0257 (3)	
N11	0.1889 (6)	0.6363 (3)	0.2071 (6)	0.0150 (9)	
C12	0.3133 (7)	0.6947 (4)	0.2214 (7)	0.0175 (12)	
C13	0.2617 (8)	0.7753 (4)	0.2035 (8)	0.0226 (12)	
H13	0.348279	0.816701	0.214521	0.027*	
C14	0.0857 (7)	0.7958 (3)	0.1698 (8)	0.0216 (12)	
H14	0.050262	0.851025	0.155956	0.026*	
C15	−0.0382 (8)	0.7352 (4)	0.1565 (10)	0.0230 (13)	
H15	−0.160284	0.747910	0.133755	0.028*	
C16	0.0171 (7)	0.6567 (4)	0.1764 (8)	0.0201 (12)	
H16	−0.068217	0.614881	0.168460	0.024*	
C17	0.5037 (7)	0.6680 (3)	0.2567 (10)	0.0287 (15)	
H17A	0.521166	0.635987	0.155039	0.043*	
H17B	0.532990	0.634864	0.365612	0.043*	
H17C	0.580432	0.715824	0.272402	0.043*	

### Trichlorido(2-methylpyridine-κN)gold(III) (1b) Atomic displacement parameters (Å^2^)

**Table:**

	*U*^11^	*U*^22^	*U*^33^	*U*^12^	*U*^13^	*U*^23^
Au1	0.01750 (10)	0.01050 (9)	0.01386 (9)	−0.00089 (10)	0.00467 (7)	−0.00015 (10)
Cl1	0.0253 (7)	0.0121 (7)	0.0248 (7)	0.0018 (5)	0.0061 (6)	0.0012 (5)
Cl2	0.0381 (8)	0.0163 (7)	0.0138 (7)	−0.0021 (6)	0.0088 (7)	−0.0005 (5)
Cl3	0.0375 (8)	0.0243 (8)	0.0174 (7)	0.0053 (7)	0.0105 (7)	0.0011 (6)
N11	0.019 (2)	0.008 (2)	0.017 (2)	0.0010 (17)	0.0030 (19)	−0.0011 (19)
C12	0.018 (3)	0.018 (3)	0.017 (3)	−0.002 (2)	0.005 (2)	−0.001 (2)
C13	0.025 (3)	0.015 (3)	0.027 (3)	−0.003 (3)	0.006 (3)	−0.001 (3)
C14	0.027 (3)	0.013 (3)	0.026 (3)	0.003 (2)	0.008 (3)	0.000 (2)
C15	0.021 (3)	0.022 (3)	0.026 (3)	0.004 (2)	0.005 (3)	−0.007 (3)
C16	0.018 (3)	0.017 (3)	0.026 (3)	−0.005 (2)	0.007 (2)	−0.006 (3)
C17	0.021 (3)	0.016 (3)	0.049 (4)	−0.004 (2)	0.007 (3)	0.001 (3)

### Trichlorido(2-methylpyridine-κN)gold(III) (1b) Geometric parameters (Å, º)

**Table:**

Au1—N11	2.030 (4)	C13—H13	0.9500
Au1—Cl3	2.2652 (14)	C14—C15	1.378 (8)
Au1—Cl1	2.2688 (13)	C14—H14	0.9500
Au1—Cl2	2.2766 (12)	C15—C16	1.361 (8)
N11—C16	1.347 (6)	C15—H15	0.9500
N11—C12	1.354 (7)	C16—H16	0.9500
C12—C13	1.385 (8)	C17—H17A	0.9800
C12—C17	1.512 (7)	C17—H17B	0.9800
C13—C14	1.378 (7)	C17—H17C	0.9800
			
N11—Au1—Cl3	89.65 (14)	C13—C14—C15	119.1 (6)
N11—Au1—Cl1	178.32 (13)	C13—C14—H14	120.4
Cl3—Au1—Cl1	91.17 (5)	C15—C14—H14	120.4
N11—Au1—Cl2	88.27 (13)	C16—C15—C14	118.9 (6)
Cl3—Au1—Cl2	177.79 (5)	C16—C15—H15	120.6
Cl1—Au1—Cl2	90.90 (5)	C14—C15—H15	120.6
C16—N11—C12	120.1 (5)	N11—C16—C15	122.2 (5)
C16—N11—Au1	117.9 (4)	N11—C16—H16	118.9
C12—N11—Au1	121.9 (4)	C15—C16—H16	118.9
N11—C12—C13	119.3 (5)	C12—C17—H17A	109.5
N11—C12—C17	117.5 (5)	C12—C17—H17B	109.5
C13—C12—C17	123.2 (5)	H17A—C17—H17B	109.5
C14—C13—C12	120.5 (5)	C12—C17—H17C	109.5
C14—C13—H13	119.8	H17A—C17—H17C	109.5
C12—C13—H13	119.8	H17B—C17—H17C	109.5
			
Cl3—Au1—N11—C16	−86.0 (4)	N11—C12—C13—C14	−0.6 (8)
Cl2—Au1—N11—C16	93.3 (4)	C17—C12—C13—C14	179.4 (6)
Cl3—Au1—N11—C12	98.2 (4)	C12—C13—C14—C15	0.8 (9)
Cl2—Au1—N11—C12	−82.5 (4)	C13—C14—C15—C16	−0.2 (10)
C16—N11—C12—C13	−0.2 (8)	C12—N11—C16—C15	0.9 (9)
Au1—N11—C12—C13	175.5 (4)	Au1—N11—C16—C15	−175.0 (5)
C16—N11—C12—C17	179.7 (5)	C14—C15—C16—N11	−0.6 (10)
Au1—N11—C12—C17	−4.6 (7)		

### Trichlorido(2-methylpyridine-κN)gold(III) (1b) Hydrogen-bond geometry (Å, º)

**Table:**

*D*—H···*A*	*D*—H	H···*A*	*D*···*A*	*D*—H···*A*
C13—H13···Cl1^i^	0.95	2.79	3.683 (6)	157
C15—H15···Cl1^ii^	0.95	2.88	3.527 (6)	126
C17—H17*C*···Cl1^i^	0.98	2.86	3.798 (6)	160
C16—H16···Cl2^iii^	0.95	2.70	3.610 (6)	162
C17—H17*B*···Cl3^iv^	0.98	3.00	3.771 (6)	137

Symmetry codes: (i) −*x*+1, *y*+1/2, −*z*+1/2; (ii) −*x*, *y*+1/2, −*z*+1/2; (iii) −*x*, −*y*+1, −*z*; (iv) −*x*+1, −*y*+1, −*z*+1.

### Tribromido(2-methylpyridine-κN)gold(III) (2) Crystal data

**Table:**

[AuBr_3_(C_6_H_7_N)]	*F*(000) = 936
*M**_r_* = 529.82	*D*_x_ = 3.322 Mg m^−^^3^
Monoclinic, *P*2_1_/*n*	Mo *K*α radiation, λ = 0.71073 Å
*a* = 8.0776 (4) Å	Cell parameters from 7970 reflections
*b* = 14.7230 (7) Å	θ = 3.2–29.2°
*c* = 8.9682 (4) Å	µ = 25.14 mm^−^^1^
β = 96.617 (5)°	*T* = 100 K
*V* = 1059.45 (9) Å^3^	Cube, red
*Z* = 4	0.07 × 0.07 × 0.07 mm

### Tribromido(2-methylpyridine-κN)gold(III) (2) Data collection

**Table:**

Oxford Diffraction Xcalibur, Eos diffractometer	3597 independent reflections
Radiation source: Enhance (Mo) X-ray Source	2716 reflections with *I* > 2σ(*I*)
Graphite monochromator	*R*_int_ = –
Detector resolution: 16.1419 pixels mm^-1^	θ_max_ = 28.3°, θ_min_ = 3.2°
ω scans	*h* = −10→10
Absorption correction: multi-scan (CrysAlisPro; Rigaku OD, 2020)	*k* = −19→19
*T*_min_ = 0.571, *T*_max_ = 1.000	*l* = −11→11
3597 measured reflections	

### Tribromido(2-methylpyridine-κN)gold(III) (2) Refinement

**Table:**

Refinement on *F*^2^	Primary atom site location: structure-invariant direct methods
Least-squares matrix: full	Secondary atom site location: difference Fourier map
*R*[*F*^2^ > 2σ(*F*^2^)] = 0.028	Hydrogen site location: inferred from neighbouring sites
*wR*(*F*^2^) = 0.040	H-atom parameters constrained
*S* = 0.80	*w* = 1/[σ^2^(*F*_o_^2^) + (0.0102*P*)^2^] where *P* = (*F*_o_^2^ + 2*F*_c_^2^)/3
3597 reflections	(Δ/σ)_max_ = 0.001
102 parameters	Δρ_max_ = 1.26 e Å^−^^3^
0 restraints	Δρ_min_ = −1.40 e Å^−^^3^

### Tribromido(2-methylpyridine-κN)gold(III) (2) Fractional atomic coordinates and isotropic or equivalent isotropic displacement parameters (Å^2^)

**Table:**

	*x*	*y*	*z*	*U*_iso_*/*U*_eq_	
Au1	0.32350 (4)	0.61053 (2)	0.91242 (3)	0.01172 (7)	
Br1	0.39636 (9)	0.69112 (5)	1.14322 (8)	0.01898 (18)	
Br2	0.61485 (9)	0.59456 (5)	0.87711 (8)	0.01859 (19)	
Br3	0.03134 (9)	0.62958 (5)	0.93519 (8)	0.01940 (19)	
N11	0.2616 (7)	0.5394 (3)	0.7174 (6)	0.0123 (14)	
C12	0.2659 (8)	0.5774 (5)	0.5798 (7)	0.0125 (16)	
C13	0.2314 (9)	0.5240 (5)	0.4544 (8)	0.0198 (19)	
H13	0.235011	0.549813	0.357770	0.024*	
C14	0.1919 (9)	0.4339 (5)	0.4666 (8)	0.0202 (19)	
H14	0.169881	0.397384	0.379219	0.024*	
C15	0.1846 (9)	0.3971 (5)	0.6052 (8)	0.0194 (16)	
H15	0.155268	0.335136	0.615523	0.023*	
C16	0.2203 (9)	0.4512 (5)	0.7299 (8)	0.0197 (18)	
H16	0.215791	0.425899	0.826817	0.024*	
C17	0.3074 (8)	0.6761 (4)	0.5701 (7)	0.0181 (18)	
H17A	0.226556	0.712064	0.618463	0.027*	
H17B	0.303266	0.693940	0.464416	0.027*	
H17C	0.419646	0.687021	0.620966	0.027*	

### Tribromido(2-methylpyridine-κN)gold(III) (2) Atomic displacement parameters (Å^2^)

**Table:**

	*U*^11^	*U*^22^	*U*^33^	*U*^12^	*U*^13^	*U*^23^
Au1	0.01248 (15)	0.01227 (12)	0.01070 (13)	−0.00019 (14)	0.00261 (11)	−0.00070 (14)
Br1	0.0246 (5)	0.0187 (4)	0.0136 (4)	−0.0007 (4)	0.0021 (3)	−0.0033 (3)
Br2	0.0137 (4)	0.0249 (5)	0.0175 (4)	−0.0014 (4)	0.0034 (3)	−0.0014 (4)
Br3	0.0139 (4)	0.0250 (4)	0.0197 (4)	0.0033 (3)	0.0039 (3)	−0.0007 (4)
N11	0.016 (4)	0.012 (3)	0.010 (3)	0.000 (3)	0.004 (3)	0.000 (3)
C12	0.009 (4)	0.015 (4)	0.014 (4)	0.006 (3)	0.005 (3)	0.001 (3)
C13	0.022 (5)	0.027 (5)	0.010 (4)	0.000 (4)	0.003 (4)	0.008 (3)
C14	0.025 (5)	0.019 (4)	0.015 (4)	−0.002 (4)	−0.003 (4)	−0.007 (3)
C15	0.022 (4)	0.013 (4)	0.023 (4)	−0.004 (4)	0.000 (3)	−0.003 (4)
C16	0.017 (5)	0.017 (4)	0.025 (4)	−0.001 (4)	0.003 (4)	0.005 (4)
C17	0.027 (5)	0.018 (4)	0.010 (4)	−0.004 (4)	0.005 (3)	0.006 (3)

### Tribromido(2-methylpyridine-κN)gold(III) (2) Geometric parameters (Å, º)

**Table:**

Au1—N11	2.050 (5)	C13—H13	0.9500
Au1—Br1	2.3996 (7)	C14—C15	1.363 (9)
Au1—Br3	2.4085 (8)	C14—H14	0.9500
Au1—Br2	2.4220 (8)	C15—C16	1.377 (8)
N11—C16	1.349 (7)	C15—H15	0.9500
N11—C12	1.359 (8)	C16—H16	0.9500
C12—C13	1.375 (8)	C17—H17A	0.9800
C12—C17	1.496 (9)	C17—H17B	0.9800
C13—C14	1.372 (9)	C17—H17C	0.9800
			
N11—Au1—Br1	178.93 (15)	C15—C14—C13	119.5 (7)
N11—Au1—Br3	89.24 (16)	C15—C14—H14	120.3
Br1—Au1—Br3	90.84 (3)	C13—C14—H14	120.3
N11—Au1—Br2	89.08 (16)	C14—C15—C16	119.0 (7)
Br1—Au1—Br2	90.88 (3)	C14—C15—H15	120.5
Br3—Au1—Br2	177.16 (3)	C16—C15—H15	120.5
C16—N11—C12	120.3 (6)	N11—C16—C15	121.4 (7)
C16—N11—Au1	117.3 (5)	N11—C16—H16	119.3
C12—N11—Au1	122.4 (4)	C15—C16—H16	119.3
N11—C12—C13	118.9 (6)	C12—C17—H17A	109.5
N11—C12—C17	118.8 (6)	C12—C17—H17B	109.5
C13—C12—C17	122.3 (6)	H17A—C17—H17B	109.5
C14—C13—C12	121.0 (7)	C12—C17—H17C	109.5
C14—C13—H13	119.5	H17A—C17—H17C	109.5
C12—C13—H13	119.5	H17B—C17—H17C	109.5
			
Br3—Au1—N11—C16	−80.7 (5)	N11—C12—C13—C14	0.5 (10)
Br2—Au1—N11—C16	101.6 (5)	C17—C12—C13—C14	−179.3 (7)
Br3—Au1—N11—C12	101.2 (5)	C12—C13—C14—C15	0.9 (11)
Br2—Au1—N11—C12	−76.5 (5)	C13—C14—C15—C16	−1.2 (11)
C16—N11—C12—C13	−1.5 (10)	C12—N11—C16—C15	1.2 (10)
Au1—N11—C12—C13	176.5 (5)	Au1—N11—C16—C15	−176.9 (5)
C16—N11—C12—C17	178.2 (6)	C14—C15—C16—N11	0.2 (11)
Au1—N11—C12—C17	−3.7 (8)		

### Tribromido(2-methylpyridine-κN)gold(III) (2) Hydrogen-bond geometry (Å, º)

**Table:**

*D*—H···*A*	*D*—H	H···*A*	*D*···*A*	*D*—H···*A*
C13—H13···Br1^i^	0.95	3.21	4.059 (7)	150
C14—H14···Br1^ii^	0.95	3.09	3.754 (7)	129
C15—H15···Br1^ii^	0.95	3.14	3.775 (7)	126
C17—H17*A*···Br1^iii^	0.98	3.05	3.972 (7)	156
C17—H17*B*···Br1^i^	0.98	3.06	3.982 (6)	157
C14—H14···Br2^iv^	0.95	3.04	3.636 (7)	122
C16—H16···Br2^v^	0.95	2.86	3.683 (7)	146
C17—H17*C*···Br2	0.98	2.96	3.690 (7)	132
C14—H14···Br3^vi^	0.95	3.11	3.951 (7)	148

Symmetry codes: (i) *x*, *y*, *z*−1; (ii) −*x*+1/2, *y*−1/2, −*z*+3/2; (iii) *x*−1/2, −*y*+3/2, *z*−1/2; (iv) −*x*+1, −*y*+1, −*z*+1; (v) −*x*+1, −*y*+1, −*z*+2; (vi) −*x*, −*y*+1, −*z*+1.

### Tribromido(3-methylpyridine-κN)gold(III) (3) Crystal data

**Table:**

[AuBr_3_(C_6_H_7_N)]	*F*(000) = 1872
*M**_r_* = 529.82	*D*_x_ = 3.433 Mg m^−^^3^
Monoclinic, *C*2/*c*	Mo *K*α radiation, λ = 0.71073 Å
*a* = 18.6763 (6) Å	Cell parameters from 7609 reflections
*b* = 6.78409 (14) Å	θ = 2.5–30.2°
*c* = 18.4807 (5) Å	µ = 25.99 mm^−^^1^
β = 118.887 (4)°	*T* = 100 K
*V* = 2050.18 (12) Å^3^	Plate, red
*Z* = 8	0.12 × 0.10 × 0.02 mm

### Tribromido(3-methylpyridine-κN)gold(III) (3) Data collection

**Table:**

Oxford Diffraction Xcalibur, Eos diffractometer	3120 independent reflections
Radiation source: Enhance (Mo) X-ray Source	2563 reflections with *I* > 2σ(*I*)
Detector resolution: 16.1419 pixels mm^-1^	*R*_int_ = 0.064
ω scan	θ_max_ = 31.0°, θ_min_ = 2.5°
Absorption correction: multi-scan (CrysAlisPro; Rigaku OD, 2020)	*h* = −26→26
*T*_min_ = 0.309, *T*_max_ = 1.000	*k* = −9→9
40540 measured reflections	*l* = −25→26

### Tribromido(3-methylpyridine-κN)gold(III) (3) Refinement

**Table:**

Refinement on *F*^2^	Primary atom site location: structure-invariant direct methods
Least-squares matrix: full	Secondary atom site location: difference Fourier map
*R*[*F*^2^ > 2σ(*F*^2^)] = 0.026	Hydrogen site location: inferred from neighbouring sites
*wR*(*F*^2^) = 0.054	H-atom parameters constrained
*S* = 1.04	*w* = 1/[σ^2^(*F*_o_^2^) + (0.0194*P*)^2^ + 10.7877*P*] where *P* = (*F*_o_^2^ + 2*F*_c_^2^)/3
3120 reflections	(Δ/σ)_max_ = 0.001
101 parameters	Δρ_max_ = 2.01 e Å^−^^3^
0 restraints	Δρ_min_ = −1.71 e Å^−^^3^

### Tribromido(3-methylpyridine-κN)gold(III) (3) Fractional atomic coordinates and isotropic or equivalent isotropic displacement parameters (Å^2^)

**Table:**

	*x*	*y*	*z*	*U*_iso_*/*U*_eq_	
Au1	0.25213 (2)	0.25929 (2)	0.37416 (2)	0.01140 (5)	
Br1	0.10545 (3)	0.26127 (7)	0.29684 (3)	0.01921 (10)	
Br2	0.25822 (3)	0.55197 (7)	0.30223 (3)	0.01973 (11)	
Br3	0.24913 (3)	−0.02193 (7)	0.45387 (3)	0.01697 (10)	
N11	0.3782 (2)	0.2527 (5)	0.4343 (2)	0.0120 (7)	
C12	0.4162 (3)	0.2409 (6)	0.3884 (3)	0.0135 (8)	
H12	0.384098	0.233363	0.329940	0.016*	
C13	0.4998 (3)	0.2393 (6)	0.4233 (3)	0.0142 (9)	
C14	0.5454 (3)	0.2506 (6)	0.5089 (3)	0.0151 (9)	
H14	0.603448	0.250258	0.534980	0.018*	
C15	0.5065 (3)	0.2623 (6)	0.5564 (3)	0.0150 (9)	
H15	0.537267	0.269624	0.614938	0.018*	
C16	0.4220 (3)	0.2631 (6)	0.5169 (3)	0.0141 (9)	
H16	0.394516	0.271021	0.548698	0.017*	
C17	0.5387 (3)	0.2290 (7)	0.3688 (3)	0.0205 (10)	
H17A	0.503831	0.151818	0.319215	0.031*	
H17B	0.592426	0.165859	0.398891	0.031*	
H17C	0.545228	0.362545	0.352579	0.031*	

### Tribromido(3-methylpyridine-κN)gold(III) (3) Atomic displacement parameters (Å^2^)

**Table:**

	*U*^11^	*U*^22^	*U*^33^	*U*^12^	*U*^13^	*U*^23^
Au1	0.00696 (8)	0.01682 (9)	0.00889 (8)	0.00109 (6)	0.00261 (6)	0.00037 (6)
Br1	0.0074 (2)	0.0358 (3)	0.0120 (2)	0.00234 (19)	0.00275 (17)	0.00061 (18)
Br2	0.0159 (2)	0.0234 (2)	0.0155 (2)	0.00211 (18)	0.00399 (18)	0.00782 (18)
Br3	0.0128 (2)	0.0176 (2)	0.0204 (2)	0.00011 (17)	0.00787 (18)	0.00323 (17)
N11	0.0085 (18)	0.0128 (16)	0.0128 (17)	0.0012 (14)	0.0035 (14)	0.0017 (14)
C12	0.013 (2)	0.015 (2)	0.0097 (19)	−0.0018 (17)	0.0032 (17)	−0.0018 (16)
C13	0.012 (2)	0.013 (2)	0.014 (2)	−0.0020 (17)	0.0037 (17)	−0.0006 (16)
C14	0.010 (2)	0.013 (2)	0.015 (2)	−0.0001 (17)	0.0000 (17)	−0.0013 (17)
C15	0.011 (2)	0.016 (2)	0.013 (2)	−0.0004 (17)	0.0026 (17)	−0.0010 (17)
C16	0.016 (2)	0.012 (2)	0.013 (2)	−0.0002 (17)	0.0065 (18)	0.0013 (16)
C17	0.015 (2)	0.030 (3)	0.019 (2)	−0.001 (2)	0.010 (2)	−0.0009 (19)

### Tribromido(3-methylpyridine-κN)gold(III) (3) Geometric parameters (Å, º)

**Table:**

Au1—N11	2.062 (4)	C13—C17	1.501 (6)
Au1—Br1	2.4009 (5)	C14—C15	1.386 (6)
Au1—Br2	2.4225 (5)	C14—H14	0.9500
Au1—Br3	2.4276 (5)	C15—C16	1.382 (6)
N11—C16	1.341 (6)	C15—H15	0.9500
N11—C12	1.346 (6)	C16—H16	0.9500
C12—C13	1.372 (6)	C17—H17A	0.9800
C12—H12	0.9500	C17—H17B	0.9800
C13—C14	1.391 (6)	C17—H17C	0.9800
			
N11—Au1—Br1	176.62 (10)	C15—C14—C13	120.3 (4)
N11—Au1—Br2	88.25 (10)	C15—C14—H14	119.9
Br1—Au1—Br2	90.649 (17)	C13—C14—H14	119.9
N11—Au1—Br3	90.63 (10)	C16—C15—C14	118.7 (4)
Br1—Au1—Br3	90.653 (17)	C16—C15—H15	120.7
Br2—Au1—Br3	176.494 (17)	C14—C15—H15	120.7
C16—N11—C12	120.3 (4)	N11—C16—C15	120.9 (4)
C16—N11—Au1	121.4 (3)	N11—C16—H16	119.5
C12—N11—Au1	118.3 (3)	C15—C16—H16	119.5
N11—C12—C13	122.1 (4)	C13—C17—H17A	109.5
N11—C12—H12	119.0	C13—C17—H17B	109.5
C13—C12—H12	119.0	H17A—C17—H17B	109.5
C12—C13—C14	117.8 (4)	C13—C17—H17C	109.5
C12—C13—C17	119.6 (4)	H17A—C17—H17C	109.5
C14—C13—C17	122.5 (4)	H17B—C17—H17C	109.5
			
Br2—Au1—N11—C16	120.5 (3)	N11—C12—C13—C17	−179.1 (4)
Br3—Au1—N11—C16	−56.1 (3)	C12—C13—C14—C15	0.2 (6)
Br2—Au1—N11—C12	−58.4 (3)	C17—C13—C14—C15	179.2 (4)
Br3—Au1—N11—C12	124.9 (3)	C13—C14—C15—C16	−0.2 (6)
C16—N11—C12—C13	−0.1 (6)	C12—N11—C16—C15	0.2 (6)
Au1—N11—C12—C13	178.8 (3)	Au1—N11—C16—C15	−178.8 (3)
N11—C12—C13—C14	0.0 (6)	C14—C15—C16—N11	0.0 (6)

### Tribromido(3-methylpyridine-κN)gold(III) (3) Hydrogen-bond geometry (Å, º)

**Table:**

*D*—H···*A*	*D*—H	H···*A*	*D*···*A*	*D*—H···*A*
C15—H15···Br1^i^	0.95	2.97	3.905 (5)	168
C16—H16···Br1^ii^	0.95	2.86	3.721 (5)	151
C12—H12···Br2^iii^	0.95	2.88	3.684 (4)	144
C14—H14···Br3^iv^	0.95	3.08	3.880 (5)	143
C17—H17*B*···Br3^iv^	0.98	3.06	3.984 (5)	159

Symmetry codes: (i) *x*+1/2, −*y*+1/2, *z*+1/2; (ii) −*x*+1/2, −*y*+1/2, −*z*+1; (iii) −*x*+1/2, *y*−1/2, −*z*+1/2; (iv) −*x*+1, −*y*, −*z*+1.

### Tribromido(2,4-dimethylpyridine-κN)gold(III) (4) Crystal data

**Table:**

[AuBr_3_(C_7_H_9_N)]	*F*(000) = 1936
*M**_r_* = 543.85	*D*_x_ = 3.166 Mg m^−^^3^
Monoclinic, *P*2_1_/*c*	Mo *K*α radiation, λ = 0.71073 Å
*a* = 8.07477 (17) Å	Cell parameters from 19274 reflections
*b* = 17.2853 (4) Å	θ = 2.5–30.3°
*c* = 16.3527 (4) Å	µ = 23.35 mm^−^^1^
β = 90.818 (2)°	*T* = 100 K
*V* = 2282.19 (8) Å^3^	Plate, red
*Z* = 8	0.10 × 0.10 × 0.02 mm

### Tribromido(2,4-dimethylpyridine-κN)gold(III) (4) Data collection

**Table:**

Oxford Diffraction Xcalibur, Eos diffractometer	6677 independent reflections
Radiation source: Enhance (Mo) X-ray Source	5614 reflections with *I* > 2σ(*I*)
Graphite monochromator	*R*_int_ = 0.086
Detector resolution: 16.1419 pixels mm^-1^	θ_max_ = 30.0°, θ_min_ = 2.4°
ω scan	*h* = −11→11
Absorption correction: multi-scan (CrysAlisPro; Rigaku OD, 2020)	*k* = −24→24
*T*_min_ = 0.338, *T*_max_ = 1.000	*l* = −22→22
139727 measured reflections	

### Tribromido(2,4-dimethylpyridine-κN)gold(III) (4) Refinement

**Table:**

Refinement on *F*^2^	Secondary atom site location: difference Fourier map
Least-squares matrix: full	Hydrogen site location: inferred from neighbouring sites
*R*[*F*^2^ > 2σ(*F*^2^)] = 0.026	H-atom parameters constrained
*wR*(*F*^2^) = 0.048	*w* = 1/[σ^2^(*F*_o_^2^) + (0.0166*P*)^2^ + 4.8838*P*] where *P* = (*F*_o_^2^ + 2*F*_c_^2^)/3
*S* = 1.05	(Δ/σ)_max_ = 0.001
6677 reflections	Δρ_max_ = 1.39 e Å^−^^3^
222 parameters	Δρ_min_ = −1.59 e Å^−^^3^
0 restraints	Extinction correction: SHELXL-2019/3 (Sheldrick, 2015), Fc^*^=kFc[1+0.001xFc^2^λ^3^/sin(2θ)]^-1/4^
Primary atom site location: structure-invariant direct methods	Extinction coefficient: 0.000318 (17)

### Tribromido(2,4-dimethylpyridine-κN)gold(III) (4) Fractional atomic coordinates and isotropic or equivalent isotropic displacement parameters (Å^2^)

**Table:**

	*x*	*y*	*z*	*U*_iso_*/*U*_eq_	
Au1	0.37066 (2)	0.53353 (2)	0.11463 (2)	0.01033 (4)	
Br1	0.35705 (5)	0.66385 (2)	0.06436 (3)	0.01563 (9)	
Br2	0.66835 (5)	0.53128 (2)	0.09789 (3)	0.01426 (9)	
Br3	0.07450 (5)	0.53012 (3)	0.12900 (3)	0.01837 (10)	
N11	0.3818 (4)	0.4216 (2)	0.1579 (2)	0.0121 (7)	
C12	0.4170 (5)	0.4049 (3)	0.2370 (3)	0.0130 (8)	
C13	0.4288 (5)	0.3282 (3)	0.2610 (3)	0.0151 (9)	
H13	0.454690	0.316512	0.316517	0.018*	
C14	0.4035 (5)	0.2683 (3)	0.2061 (3)	0.0162 (9)	
C15	0.3669 (5)	0.2879 (3)	0.1251 (3)	0.0150 (9)	
H15	0.348718	0.248629	0.085306	0.018*	
C16	0.3573 (5)	0.3649 (3)	0.1036 (3)	0.0166 (9)	
H16	0.332571	0.378020	0.048345	0.020*	
C17	0.4415 (6)	0.4704 (3)	0.2961 (3)	0.0183 (9)	
H17A	0.530902	0.503920	0.277120	0.027*	
H17B	0.470737	0.449597	0.350165	0.027*	
H17C	0.338832	0.500328	0.299634	0.027*	
C18	0.4170 (6)	0.1852 (3)	0.2307 (3)	0.0244 (11)	
H18A	0.531110	0.167388	0.223336	0.037*	
H18B	0.341891	0.153968	0.196547	0.037*	
H18C	0.386945	0.179641	0.288221	0.037*	
Au2	0.05459 (2)	0.61831 (2)	0.47994 (2)	0.01028 (4)	
Br4	0.04989 (6)	0.59206 (3)	0.33604 (3)	0.01625 (9)	
Br5	−0.23631 (5)	0.58457 (3)	0.48885 (3)	0.01582 (9)	
Br6	0.34824 (5)	0.64545 (3)	0.47627 (3)	0.01783 (9)	
N21	0.0611 (4)	0.6427 (2)	0.6029 (2)	0.0109 (7)	
C22	−0.0021 (5)	0.7083 (2)	0.6339 (3)	0.0125 (8)	
C23	0.0127 (5)	0.7219 (3)	0.7175 (3)	0.0141 (9)	
H23	−0.029797	0.768577	0.739524	0.017*	
C24	0.0882 (6)	0.6686 (3)	0.7695 (3)	0.0157 (9)	
C25	0.1492 (5)	0.6013 (2)	0.7343 (3)	0.0138 (9)	
H25	0.200804	0.563014	0.767731	0.017*	
C26	0.1350 (5)	0.5900 (2)	0.6520 (3)	0.0139 (9)	
H26	0.178270	0.544038	0.628684	0.017*	
C27	−0.0856 (6)	0.7651 (3)	0.5782 (3)	0.0166 (9)	
H27A	−0.015775	0.774566	0.530717	0.025*	
H27B	−0.103312	0.813776	0.607504	0.025*	
H27C	−0.192617	0.744148	0.559799	0.025*	
C28	0.1065 (6)	0.6835 (3)	0.8595 (3)	0.0225 (11)	
H28A	0.214812	0.707064	0.870927	0.034*	
H28B	0.098020	0.634500	0.889310	0.034*	
H28C	0.018761	0.718645	0.877136	0.034*	

### Tribromido(2,4-dimethylpyridine-κN)gold(III) (4) Atomic displacement parameters (Å^2^)

**Table:**

	*U*^11^	*U*^22^	*U*^33^	*U*^12^	*U*^13^	*U*^23^
Au1	0.00968 (8)	0.00956 (8)	0.01173 (8)	0.00064 (6)	−0.00049 (6)	0.00167 (6)
Br1	0.0168 (2)	0.0106 (2)	0.0194 (2)	0.00176 (16)	−0.00143 (17)	0.00387 (16)
Br2	0.00955 (19)	0.0156 (2)	0.0176 (2)	0.00043 (16)	−0.00044 (16)	0.00493 (16)
Br3	0.0101 (2)	0.0200 (2)	0.0250 (2)	0.00089 (17)	0.00161 (17)	0.00309 (18)
N11	0.0124 (17)	0.0114 (18)	0.0124 (17)	0.0013 (14)	0.0008 (14)	0.0008 (14)
C12	0.0079 (19)	0.017 (2)	0.014 (2)	−0.0007 (16)	0.0059 (16)	0.0008 (17)
C13	0.017 (2)	0.016 (2)	0.013 (2)	−0.0017 (17)	−0.0005 (17)	0.0037 (17)
C14	0.012 (2)	0.015 (2)	0.021 (2)	0.0010 (17)	0.0005 (17)	0.0049 (18)
C15	0.015 (2)	0.014 (2)	0.016 (2)	0.0001 (17)	−0.0012 (17)	−0.0027 (17)
C16	0.016 (2)	0.017 (2)	0.017 (2)	−0.0007 (17)	−0.0017 (18)	0.0011 (17)
C17	0.021 (2)	0.016 (2)	0.018 (2)	−0.0007 (19)	−0.0022 (18)	−0.0007 (18)
C18	0.032 (3)	0.015 (2)	0.026 (3)	0.001 (2)	−0.005 (2)	0.005 (2)
Au2	0.01084 (8)	0.00909 (8)	0.01092 (8)	0.00035 (6)	0.00056 (6)	−0.00007 (6)
Br4	0.0218 (2)	0.0154 (2)	0.0117 (2)	0.00044 (17)	0.00153 (17)	−0.00077 (16)
Br5	0.0116 (2)	0.0174 (2)	0.0184 (2)	−0.00171 (16)	0.00051 (16)	−0.00224 (17)
Br6	0.0121 (2)	0.0191 (2)	0.0223 (2)	−0.00219 (17)	0.00266 (17)	−0.00037 (18)
N21	0.0082 (16)	0.0091 (17)	0.0155 (18)	−0.0009 (13)	−0.0010 (14)	0.0004 (14)
C22	0.010 (2)	0.011 (2)	0.017 (2)	−0.0029 (16)	−0.0015 (16)	0.0004 (16)
C23	0.014 (2)	0.016 (2)	0.013 (2)	−0.0004 (17)	0.0030 (16)	−0.0002 (17)
C24	0.018 (2)	0.017 (2)	0.012 (2)	−0.0076 (18)	−0.0016 (17)	−0.0004 (17)
C25	0.014 (2)	0.013 (2)	0.014 (2)	−0.0005 (16)	−0.0019 (17)	0.0028 (16)
C26	0.014 (2)	0.011 (2)	0.017 (2)	0.0015 (16)	−0.0021 (17)	0.0010 (16)
C27	0.021 (2)	0.010 (2)	0.018 (2)	0.0028 (17)	−0.0009 (18)	−0.0024 (17)
C28	0.033 (3)	0.022 (3)	0.013 (2)	−0.002 (2)	−0.005 (2)	−0.0034 (18)

### Tribromido(2,4-dimethylpyridine-κN)gold(III) (4) Geometric parameters (Å, º)

**Table:**

Au1—N11	2.062 (3)	Au2—N21	2.054 (4)
Au1—Br1	2.3998 (4)	Au2—Br4	2.3963 (4)
Au1—Br3	2.4070 (5)	Au2—Br6	2.4187 (5)
Au1—Br2	2.4235 (4)	Au2—Br5	2.4266 (5)
N11—C16	1.336 (5)	N21—C22	1.346 (5)
N11—C12	1.350 (5)	N21—C26	1.348 (5)
C12—C13	1.385 (6)	C22—C23	1.390 (6)
C12—C17	1.500 (6)	C22—C27	1.493 (6)
C13—C14	1.384 (6)	C23—C24	1.388 (6)
C13—H13	0.9500	C23—H23	0.9500
C14—C15	1.394 (6)	C24—C25	1.392 (6)
C14—C18	1.496 (6)	C24—C28	1.499 (6)
C15—C16	1.379 (6)	C25—C26	1.364 (6)
C15—H15	0.9500	C25—H25	0.9500
C16—H16	0.9500	C26—H26	0.9500
C17—H17A	0.9800	C27—H27A	0.9800
C17—H17B	0.9800	C27—H27B	0.9800
C17—H17C	0.9800	C27—H27C	0.9800
C18—H18A	0.9800	C28—H28A	0.9800
C18—H18B	0.9800	C28—H28B	0.9800
C18—H18C	0.9800	C28—H28C	0.9800
			
N11—Au1—Br1	179.86 (11)	N21—Au2—Br4	178.91 (10)
N11—Au1—Br3	88.97 (10)	N21—Au2—Br6	88.46 (10)
Br1—Au1—Br3	90.901 (16)	Br4—Au2—Br6	90.810 (16)
N11—Au1—Br2	89.16 (10)	N21—Au2—Br5	90.10 (10)
Br1—Au1—Br2	90.974 (15)	Br4—Au2—Br5	90.672 (16)
Br3—Au1—Br2	177.518 (17)	Br6—Au2—Br5	176.590 (17)
C16—N11—C12	120.5 (4)	C22—N21—C26	120.8 (4)
C16—N11—Au1	117.0 (3)	C22—N21—Au2	122.5 (3)
C12—N11—Au1	122.5 (3)	C26—N21—Au2	116.7 (3)
N11—C12—C13	119.3 (4)	N21—C22—C23	119.0 (4)
N11—C12—C17	118.7 (4)	N21—C22—C27	119.6 (4)
C13—C12—C17	122.0 (4)	C23—C22—C27	121.4 (4)
C14—C13—C12	121.5 (4)	C24—C23—C22	121.4 (4)
C14—C13—H13	119.3	C24—C23—H23	119.3
C12—C13—H13	119.3	C22—C23—H23	119.3
C13—C14—C15	117.5 (4)	C23—C24—C25	117.1 (4)
C13—C14—C18	122.3 (4)	C23—C24—C28	121.6 (4)
C15—C14—C18	120.1 (4)	C25—C24—C28	121.3 (4)
C16—C15—C14	119.1 (4)	C26—C25—C24	120.1 (4)
C16—C15—H15	120.4	C26—C25—H25	119.9
C14—C15—H15	120.4	C24—C25—H25	119.9
N11—C16—C15	122.1 (4)	N21—C26—C25	121.5 (4)
N11—C16—H16	119.0	N21—C26—H26	119.3
C15—C16—H16	119.0	C25—C26—H26	119.3
C12—C17—H17A	109.5	C22—C27—H27A	109.5
C12—C17—H17B	109.5	C22—C27—H27B	109.5
H17A—C17—H17B	109.5	H27A—C27—H27B	109.5
C12—C17—H17C	109.5	C22—C27—H27C	109.5
H17A—C17—H17C	109.5	H27A—C27—H27C	109.5
H17B—C17—H17C	109.5	H27B—C27—H27C	109.5
C14—C18—H18A	109.5	C24—C28—H28A	109.5
C14—C18—H18B	109.5	C24—C28—H28B	109.5
H18A—C18—H18B	109.5	H28A—C28—H28B	109.5
C14—C18—H18C	109.5	C24—C28—H28C	109.5
H18A—C18—H18C	109.5	H28A—C28—H28C	109.5
H18B—C18—H18C	109.5	H28B—C28—H28C	109.5
			
Br3—Au1—N11—C16	84.7 (3)	Br6—Au2—N21—C22	106.4 (3)
Br2—Au1—N11—C16	−93.7 (3)	Br5—Au2—N21—C22	−76.7 (3)
Br3—Au1—N11—C12	−97.3 (3)	Br6—Au2—N21—C26	−72.5 (3)
Br2—Au1—N11—C12	84.3 (3)	Br5—Au2—N21—C26	104.4 (3)
C16—N11—C12—C13	0.4 (6)	C26—N21—C22—C23	1.2 (6)
Au1—N11—C12—C13	−177.5 (3)	Au2—N21—C22—C23	−177.6 (3)
C16—N11—C12—C17	−179.2 (4)	C26—N21—C22—C27	−179.1 (4)
Au1—N11—C12—C17	2.8 (5)	Au2—N21—C22—C27	2.1 (5)
N11—C12—C13—C14	−0.6 (6)	N21—C22—C23—C24	−1.2 (6)
C17—C12—C13—C14	179.0 (4)	C27—C22—C23—C24	179.1 (4)
C12—C13—C14—C15	0.4 (6)	C22—C23—C24—C25	0.3 (6)
C12—C13—C14—C18	179.4 (4)	C22—C23—C24—C28	179.1 (4)
C13—C14—C15—C16	−0.1 (6)	C23—C24—C25—C26	0.7 (6)
C18—C14—C15—C16	−179.1 (4)	C28—C24—C25—C26	−178.2 (4)
C12—N11—C16—C15	−0.1 (6)	C22—N21—C26—C25	−0.3 (6)
Au1—N11—C16—C15	178.0 (3)	Au2—N21—C26—C25	178.6 (3)
C14—C15—C16—N11	−0.1 (7)	C24—C25—C26—N21	−0.7 (7)

### Tribromido(2,4-dimethylpyridine-κN)gold(III) (4) Hydrogen-bond geometry (Å, º)

**Table:**

*D*—H···*A*	*D*—H	H···*A*	*D*···*A*	*D*—H···*A*
C16—H16···Br2^i^	0.95	2.86	3.755 (5)	158
C17—H17*C*···Br4	0.98	2.89	3.861 (5)	171
C23—H23···Br4^ii^	0.95	2.95	3.765 (4)	145
C25—H25···Br2^iii^	0.95	2.92	3.851 (4)	166
C26—H26···Br5^iv^	0.95	2.98	3.890 (4)	161
C27—H27*B*···Br3^ii^	0.98	3.07	3.855 (4)	138
C27—H27*C*···Br5	0.98	3.01	3.647 (4)	124

Symmetry codes: (i) −*x*+1, −*y*+1, −*z*; (ii) *x*, −*y*+3/2, *z*+1/2; (iii) −*x*+1, −*y*+1, −*z*+1; (iv) −*x*, −*y*+1, −*z*+1.

### Trichlorido(3,5-dimethylpyridine-κN)gold(III) (5) Crystal data

**Table:**

[AuCl_3_(C_7_H_9_N)]	*F*(000) = 752
*M**_r_* = 410.47	*D*_x_ = 2.622 Mg m^−^^3^
Monoclinic, *C*2/*c*	Mo *K*α radiation, λ = 0.71073 Å
*a* = 7.6240 (3) Å	Cell parameters from 6320 reflections
*b* = 15.9360 (6) Å	θ = 3.2–30.3°
*c* = 9.2538 (4) Å	µ = 14.86 mm^−^^1^
β = 112.333 (5)°	*T* = 100 K
*V* = 1039.97 (8) Å^3^	Block, yellow
*Z* = 4	0.20 × 0.12 × 0.04 mm

### Trichlorido(3,5-dimethylpyridine-κN)gold(III) (5) Data collection

**Table:**

Oxford Diffraction Xcalibur, Eos diffractometer	1563 independent reflections
Radiation source: Enhance (Mo) X-ray Source	1496 reflections with *I* > 2σ(*I*)
Graphite monochromator	*R*_int_ = 0.037
Detector resolution: 16.1419 pixels mm^-1^	θ_max_ = 30.9°, θ_min_ = 2.6°
ω scan	*h* = −10→10
Absorption correction: multi-scan (CrysAlisPro; Rigaku OD, 2020)	*k* = −22→22
*T*_min_ = 0.270, *T*_max_ = 1.000	*l* = −13→13
13810 measured reflections	

### Trichlorido(3,5-dimethylpyridine-κN)gold(III) (5) Refinement

**Table:**

Refinement on *F*^2^	Primary atom site location: structure-invariant direct methods
Least-squares matrix: full	Secondary atom site location: difference Fourier map
*R*[*F*^2^ > 2σ(*F*^2^)] = 0.014	Hydrogen site location: inferred from neighbouring sites
*wR*(*F*^2^) = 0.032	H-atom parameters constrained
*S* = 1.07	*w* = 1/[σ^2^(*F*_o_^2^) + (0.0144*P*)^2^ + 1.5857*P*] where *P* = (*F*_o_^2^ + 2*F*_c_^2^)/3
1563 reflections	(Δ/σ)_max_ = 0.002
58 parameters	Δρ_max_ = 1.26 e Å^−^^3^
0 restraints	Δρ_min_ = −0.88 e Å^−^^3^

### Trichlorido(3,5-dimethylpyridine-κN)gold(III) (5) Fractional atomic coordinates and isotropic or equivalent isotropic displacement parameters (Å^2^)

**Table:**

	*x*	*y*	*z*	*U*_iso_*/*U*_eq_	
Au1	0.500000	0.51448 (2)	0.750000	0.01045 (4)	
Cl1	0.500000	0.37193 (5)	0.750000	0.01757 (16)	
Cl2	0.74832 (9)	0.51503 (3)	0.66883 (7)	0.01690 (12)	
N11	0.500000	0.64233 (17)	0.750000	0.0120 (5)	
C12	0.4966 (3)	0.68410 (14)	0.6218 (3)	0.0131 (4)	
H12	0.493575	0.653448	0.532835	0.016*	
C13	0.4975 (3)	0.77107 (15)	0.6184 (3)	0.0148 (4)	
C14	0.500000	0.8142 (2)	0.750000	0.0152 (6)	
H14	0.500001	0.873853	0.750001	0.018*	
C17	0.4978 (4)	0.81692 (16)	0.4755 (3)	0.0205 (5)	
H17A	0.626920	0.818020	0.476847	0.031*	
H17B	0.452929	0.874520	0.475849	0.031*	
H17C	0.413758	0.787938	0.381216	0.031*	

### Trichlorido(3,5-dimethylpyridine-κN)gold(III) (5) Atomic displacement parameters (Å^2^)

**Table:**

	*U*^11^	*U*^22^	*U*^33^	*U*^12^	*U*^13^	*U*^23^
Au1	0.01446 (7)	0.00766 (7)	0.00989 (6)	0.000	0.00535 (5)	0.000
Cl1	0.0311 (5)	0.0091 (3)	0.0146 (4)	0.000	0.0111 (3)	0.000
Cl2	0.0189 (3)	0.0140 (3)	0.0222 (3)	0.00270 (19)	0.0128 (2)	0.0012 (2)
N11	0.0134 (13)	0.0101 (13)	0.0117 (13)	0.000	0.0039 (10)	0.000
C12	0.0157 (11)	0.0121 (10)	0.0121 (10)	−0.0004 (8)	0.0061 (9)	0.0006 (8)
C13	0.0134 (11)	0.0137 (11)	0.0174 (11)	0.0006 (8)	0.0061 (9)	0.0044 (9)
C14	0.0129 (15)	0.0099 (14)	0.0210 (17)	0.000	0.0046 (13)	0.000
C17	0.0239 (13)	0.0167 (12)	0.0218 (13)	0.0003 (9)	0.0098 (11)	0.0053 (10)

### Trichlorido(3,5-dimethylpyridine-κN)gold(III) (5) Geometric parameters (Å, º)

**Table:**

Au1—N11	2.037 (3)	C12—H12	0.9500
Au1—Cl1	2.2716 (8)	C13—C14	1.392 (3)
Au1—Cl2	2.2867 (6)	C13—C17	1.512 (3)
Au1—Cl2^i^	2.2867 (6)	C14—H14	0.9500
N11—C12	1.352 (3)	C17—H17A	0.9800
N11—C12^i^	1.352 (3)	C17—H17B	0.9800
C12—C13	1.386 (3)	C17—H17C	0.9800
			
N11—Au1—Cl1	180.0	C12—C13—C14	118.2 (2)
N11—Au1—Cl2	89.779 (14)	C12—C13—C17	120.3 (2)
Cl1—Au1—Cl2	90.221 (14)	C14—C13—C17	121.5 (2)
N11—Au1—Cl2^i^	89.779 (14)	C13—C14—C13^i^	120.8 (3)
Cl1—Au1—Cl2^i^	90.221 (14)	C13—C14—H14	119.6
Cl2—Au1—Cl2^i^	179.56 (3)	C13^i^—C14—H14	119.6
C12—N11—C12^i^	121.0 (3)	C13—C17—H17A	109.5
C12—N11—Au1	119.50 (14)	C13—C17—H17B	109.5
C12^i^—N11—Au1	119.50 (14)	H17A—C17—H17B	109.5
N11—C12—C13	120.9 (2)	C13—C17—H17C	109.5
N11—C12—H12	119.6	H17A—C17—H17C	109.5
C13—C12—H12	119.6	H17B—C17—H17C	109.5
			
Cl2—Au1—N11—C12	51.15 (11)	Au1—N11—C12—C13	−179.68 (16)
Cl2^i^—Au1—N11—C12	−128.85 (11)	N11—C12—C13—C14	−0.6 (3)
Cl2—Au1—N11—C12^i^	−128.85 (11)	N11—C12—C13—C17	178.70 (19)
Cl2^i^—Au1—N11—C12^i^	51.15 (11)	C12—C13—C14—C13^i^	0.31 (15)
C12^i^—N11—C12—C13	0.32 (16)	C17—C13—C14—C13^i^	−179.0 (3)

Symmetry code: (i) −*x*+1, *y*, −*z*+3/2.

### Trichlorido(3,5-dimethylpyridine-κN)gold(III) (5) Hydrogen-bond geometry (Å, º)

**Table:**

*D*—H···*A*	*D*—H	H···*A*	*D*···*A*	*D*—H···*A*
C12—H12···Cl1^ii^	0.95	2.67	3.564 (2)	158
C17—H17*C*···Cl1^ii^	0.98	3.00	3.666 (3)	126
C14—H14···Cl2^iii^	0.95	2.87	3.659 (3)	142
C14—H14···Cl2^iv^	0.95	2.87	3.659 (3)	142

Symmetry codes: (ii) −*x*+1, −*y*+1, −*z*+1; (iii) *x*−1/2, *y*+1/2, *z*; (iv) −*x*+3/2, *y*+1/2, −*z*+3/2.

### Tribromido(3,5-dimethylpyridine-κN)gold(III) (6) Crystal data

**Table:**

[AuBr_3_(C_7_H_9_N)]	*Z* = 2
*M**_r_* = 543.85	*F*(000) = 484
Triclinic, *P*1	*D*_x_ = 3.203 Mg m^−^^3^
*a* = 8.2506 (3) Å	Mo *K*α radiation, λ = 0.71073 Å
*b* = 8.4726 (4) Å	Cell parameters from 9505 reflections
*c* = 9.4210 (4) Å	θ = 2.5–30.5°
α = 113.828 (4)°	µ = 23.62 mm^−^^1^
β = 103.543 (4)°	*T* = 100 K
γ = 98.368 (4)°	Plate, red
*V* = 563.93 (5) Å^3^	0.21 × 0.15 × 0.02 mm

### Tribromido(3,5-dimethylpyridine-κN)gold(III) (6) Data collection

**Table:**

Oxford Diffraction Xcalibur, Eos diffractometer	3392 independent reflections
Radiation source: Enhance (Mo) X-ray Source	3017 reflections with *I* > 2σ(*I*)
Graphite monochromator	*R*_int_ = 0.071
Detector resolution: 16.1419 pixels mm^-1^	θ_max_ = 31.1°, θ_min_ = 2.5°
ω scan	*h* = −11→11
Absorption correction: analytical (CrysAlisPro; Rigaku OD, 2020)	*k* = −12→11
*T*_min_ = 0.050, *T*_max_ = 0.683	*l* = −13→13
30785 measured reflections	

### Tribromido(3,5-dimethylpyridine-κN)gold(III) (6) Refinement

**Table:**

Refinement on *F*^2^	Primary atom site location: structure-invariant direct methods
Least-squares matrix: full	Secondary atom site location: difference Fourier map
*R*[*F*^2^ > 2σ(*F*^2^)] = 0.027	Hydrogen site location: inferred from neighbouring sites
*wR*(*F*^2^) = 0.059	H-atom parameters constrained
*S* = 1.06	*w* = 1/[σ^2^(*F*_o_^2^) + (0.0212*P*)^2^ + 1.2689*P*] where *P* = (*F*_o_^2^ + 2*F*_c_^2^)/3
3392 reflections	(Δ/σ)_max_ < 0.001
111 parameters	Δρ_max_ = 1.61 e Å^−^^3^
0 restraints	Δρ_min_ = −1.50 e Å^−^^3^

### Tribromido(3,5-dimethylpyridine-κN)gold(III) (6) Special details

**Table:**

Geometry. All esds (except the esd in the dihedral angle between two l.s. planes) are estimated using the full covariance matrix. The cell esds are taken into account individually in the estimation of esds in distances, angles and torsion angles; correlations between esds in cell parameters are only used when they are defined by crystal symmetry. An approximate (isotropic) treatment of cell esds is used for estimating esds involving l.s. planes.

### Tribromido(3,5-dimethylpyridine-κN)gold(III) (6) Fractional atomic coordinates and isotropic or equivalent isotropic displacement parameters (Å^2^)

**Table:**

	*x*	*y*	*z*	*U*_iso_*/*U*_eq_	Occ. (<1)
Au1	0.12370 (2)	0.25661 (2)	0.49051 (2)	0.01349 (6)	
Br1	0.03437 (6)	0.14810 (6)	0.19990 (6)	0.01893 (10)	
Br2	0.40986 (6)	0.22579 (7)	0.48839 (6)	0.02386 (11)	
Br3	−0.16592 (6)	0.28402 (6)	0.48783 (6)	0.01976 (10)	
N11	0.2017 (4)	0.3438 (5)	0.7424 (5)	0.0147 (7)	
C12	0.2688 (6)	0.2381 (6)	0.8004 (6)	0.0168 (9)	
H12	0.279048	0.126752	0.726372	0.020*	
C13	0.3231 (5)	0.2900 (6)	0.9669 (6)	0.0158 (9)	
C14	0.3052 (6)	0.4523 (6)	1.0705 (6)	0.0190 (9)	
H14	0.340733	0.489516	1.185204	0.023*	
C15	0.2367 (6)	0.5623 (6)	1.0112 (6)	0.0151 (8)	
C16	0.1850 (6)	0.5024 (6)	0.8425 (6)	0.0166 (9)	
H16	0.137385	0.574253	0.797647	0.020*	
C17	0.4003 (6)	0.1710 (7)	1.0302 (6)	0.0214 (10)	
H17A	0.521624	0.232331	1.097981	0.032*	
H17B	0.394076	0.059044	0.937453	0.032*	
H17C	0.335545	0.144135	1.096319	0.032*	
C18	0.2204 (7)	0.7414 (6)	1.1209 (6)	0.0214 (10)	
H18A	0.124462	0.770022	1.061149	0.032*	0.5
H18B	0.328297	0.833284	1.156030	0.032*	0.5
H18C	0.197840	0.737961	1.217411	0.032*	0.5
H18D	0.309270	0.790823	1.228578	0.032*	0.5
H18E	0.105436	0.727561	1.133697	0.032*	0.5
H18F	0.235893	0.822884	1.072316	0.032*	0.5

### Tribromido(3,5-dimethylpyridine-κN)gold(III) (6) Atomic displacement parameters (Å^2^)

**Table:**

	*U*^11^	*U*^22^	*U*^33^	*U*^12^	*U*^13^	*U*^23^
Au1	0.01095 (9)	0.01390 (9)	0.01380 (9)	0.00301 (6)	0.00348 (6)	0.00499 (7)
Br1	0.0185 (2)	0.0214 (2)	0.0142 (2)	0.00721 (18)	0.00391 (17)	0.00575 (18)
Br2	0.0129 (2)	0.0346 (3)	0.0185 (2)	0.00827 (19)	0.00475 (18)	0.0063 (2)
Br3	0.0126 (2)	0.0245 (2)	0.0206 (2)	0.00569 (18)	0.00643 (18)	0.00792 (19)
N11	0.0085 (16)	0.0160 (18)	0.021 (2)	0.0032 (14)	0.0021 (15)	0.0115 (16)
C12	0.015 (2)	0.011 (2)	0.024 (2)	0.0024 (16)	0.0077 (18)	0.0066 (18)
C13	0.0098 (19)	0.017 (2)	0.020 (2)	−0.0006 (16)	0.0028 (17)	0.0105 (19)
C14	0.015 (2)	0.017 (2)	0.022 (2)	−0.0005 (18)	0.0044 (19)	0.0083 (19)
C15	0.016 (2)	0.012 (2)	0.017 (2)	0.0020 (16)	0.0054 (17)	0.0065 (17)
C16	0.015 (2)	0.013 (2)	0.022 (2)	0.0036 (17)	0.0055 (18)	0.0085 (18)
C17	0.019 (2)	0.024 (3)	0.029 (3)	0.008 (2)	0.007 (2)	0.019 (2)
C18	0.028 (3)	0.021 (2)	0.016 (2)	0.007 (2)	0.008 (2)	0.0075 (19)

### Tribromido(3,5-dimethylpyridine-κN)gold(III) (6) Geometric parameters (Å, º)

**Table:**

Au1—N11	2.078 (4)	C15—C16	1.391 (6)
Au1—Br1	2.3892 (5)	C15—C18	1.499 (6)
Au1—Br2	2.4167 (5)	C16—H16	0.9500
Au1—Br3	2.4295 (4)	C17—H17A	0.9800
N11—C16	1.344 (6)	C17—H17B	0.9800
N11—C12	1.344 (6)	C17—H17C	0.9800
C12—C13	1.382 (7)	C18—H18A	0.9800
C12—H12	0.9500	C18—H18B	0.9800
C13—C14	1.381 (6)	C18—H18C	0.9800
C13—C17	1.508 (6)	C18—H18D	0.9800
C14—C15	1.388 (6)	C18—H18E	0.9800
C14—H14	0.9500	C18—H18F	0.9800
			
N11—Au1—Br1	178.39 (10)	H17A—C17—H17B	109.5
N11—Au1—Br2	90.03 (10)	C13—C17—H17C	109.5
Br1—Au1—Br2	89.437 (17)	H17A—C17—H17C	109.5
N11—Au1—Br3	90.85 (10)	H17B—C17—H17C	109.5
Br1—Au1—Br3	89.674 (17)	C15—C18—H18A	109.5
Br2—Au1—Br3	179.045 (17)	C15—C18—H18B	109.5
C16—N11—C12	121.8 (4)	H18A—C18—H18B	109.5
C16—N11—Au1	120.0 (3)	C15—C18—H18C	109.5
C12—N11—Au1	118.2 (3)	H18A—C18—H18C	109.5
N11—C12—C13	120.5 (4)	H18B—C18—H18C	109.5
N11—C12—H12	119.7	C15—C18—H18D	109.5
C13—C12—H12	119.7	H18A—C18—H18D	141.1
C14—C13—C12	118.0 (4)	H18B—C18—H18D	56.3
C14—C13—C17	122.1 (4)	H18C—C18—H18D	56.3
C12—C13—C17	119.9 (4)	C15—C18—H18E	109.5
C13—C14—C15	121.6 (5)	H18A—C18—H18E	56.3
C13—C14—H14	119.2	H18B—C18—H18E	141.1
C15—C14—H14	119.2	H18C—C18—H18E	56.3
C14—C15—C16	117.5 (4)	H18D—C18—H18E	109.5
C14—C15—C18	122.9 (4)	C15—C18—H18F	109.5
C16—C15—C18	119.7 (4)	H18A—C18—H18F	56.3
N11—C16—C15	120.5 (4)	H18B—C18—H18F	56.3
N11—C16—H16	119.7	H18C—C18—H18F	141.1
C15—C16—H16	119.7	H18D—C18—H18F	109.5
C13—C17—H17A	109.5	H18E—C18—H18F	109.5
C13—C17—H17B	109.5		
			
Br2—Au1—N11—C16	124.0 (3)	C12—C13—C14—C15	−0.6 (7)
Br3—Au1—N11—C16	−56.3 (3)	C17—C13—C14—C15	178.9 (4)
Br2—Au1—N11—C12	−55.8 (3)	C13—C14—C15—C16	0.5 (6)
Br3—Au1—N11—C12	123.9 (3)	C13—C14—C15—C18	−178.2 (4)
C16—N11—C12—C13	0.1 (6)	C12—N11—C16—C15	−0.2 (6)
Au1—N11—C12—C13	179.9 (3)	Au1—N11—C16—C15	180.0 (3)
N11—C12—C13—C14	0.4 (6)	C14—C15—C16—N11	−0.1 (6)
N11—C12—C13—C17	−179.2 (4)	C18—C15—C16—N11	178.7 (4)

### Tribromido(3,5-dimethylpyridine-κN)gold(III) (6) Hydrogen-bond geometry (Å, º)

**Table:**

*D*—H···*A*	*D*—H	H···*A*	*D*···*A*	*D*—H···*A*
C16—H16···Br1^i^	0.95	2.91	3.792 (4)	154
C17—H17*C*···Br1^ii^	0.98	2.87	3.749 (5)	149
C18—H18*A*···Br1^i^	0.98	2.89	3.784 (5)	151
C18—H18*C*···Br3^iii^	0.98	2.93	3.902 (5)	174
C14—H14···Br2^iv^	0.95	3.02	3.826 (5)	144

Symmetry codes: (i) −*x*, −*y*+1, −*z*+1; (ii) *x*, *y*, *z*+1; (iii) −*x*, −*y*+1, −*z*+2; (iv) −*x*+1, −*y*+1, −*z*+2.

### Trichlorido(2,6-dimethylpyridine-κN)gold(III) (7) Crystal data

**Table:**

[AuCl_3_(C_7_H_9_N)]	*F*(000) = 752
*M**_r_* = 410.47	*D*_x_ = 2.580 Mg m^−^^3^
Monoclinic, *C*2/*c*	Mo *K*α radiation, λ = 0.71073 Å
*a* = 11.0184 (3) Å	Cell parameters from 11513 reflections
*b* = 10.6600 (2) Å	θ = 2.8–30.8°
*c* = 9.7760 (3) Å	µ = 14.63 mm^−^^1^
β = 113.053 (3)°	*T* = 100 K
*V* = 1056.55 (5) Å^3^	Block, yellow
*Z* = 4	0.22 × 0.20 × 0.12 mm

### Trichlorido(2,6-dimethylpyridine-κN)gold(III) (7) Data collection

**Table:**

Oxford Diffraction Xcalibur, Eos diffractometer	1600 independent reflections
Radiation source: Enhance (Mo) X-ray Source	1553 reflections with *I* > 2σ(*I*)
Graphite monochromator	*R*_int_ = 0.030
Detector resolution: 16.1419 pixels mm^-1^	θ_max_ = 31.1°, θ_min_ = 2.8°
ω scan	*h* = −15→15
Absorption correction: multi-scan (CrysAlisPro; Rigaku OD, 2020)	*k* = −15→15
*T*_min_ = 0.267, *T*_max_ = 1.000	*l* = −14→13
15246 measured reflections	

### Trichlorido(2,6-dimethylpyridine-κN)gold(III) (7) Refinement

**Table:**

Refinement on *F*^2^	Secondary atom site location: difference Fourier map
Least-squares matrix: full	Hydrogen site location: inferred from neighbouring sites
*R*[*F*^2^ > 2σ(*F*^2^)] = 0.013	H-atom parameters constrained
*wR*(*F*^2^) = 0.026	*w* = 1/[σ^2^(*F*_o_^2^) + (0.008*P*)^2^ + 2.0823*P*] where *P* = (*F*_o_^2^ + 2*F*_c_^2^)/3
*S* = 1.12	(Δ/σ)_max_ < 0.001
1600 reflections	Δρ_max_ = 0.83 e Å^−^^3^
59 parameters	Δρ_min_ = −1.17 e Å^−^^3^
0 restraints	Extinction correction: SHELXL-2019/3 (Sheldrick, 2015), Fc^*^=kFc[1+0.001xFc^2^λ^3^/sin(2θ)]^-1/4^
Primary atom site location: structure-invariant direct methods	Extinction coefficient: 0.00079 (4)

### Trichlorido(2,6-dimethylpyridine-κN)gold(III) (7) Fractional atomic coordinates and isotropic or equivalent isotropic displacement parameters (Å^2^)

**Table:**

	*x*	*y*	*z*	*U*_iso_*/*U*_eq_	
Au1	0.500000	0.07715 (2)	0.250000	0.00999 (4)	
Cl1	0.500000	−0.13530 (6)	0.250000	0.01876 (14)	
Cl2	0.67610 (5)	0.07948 (5)	0.47293 (6)	0.01738 (10)	
N11	0.500000	0.2681 (2)	0.250000	0.0116 (4)	
C12	0.5772 (2)	0.33032 (19)	0.1927 (2)	0.0134 (4)	
C13	0.5773 (2)	0.4602 (2)	0.1918 (2)	0.0188 (4)	
H13	0.630269	0.504531	0.151332	0.023*	
C14	0.500000	0.5250 (3)	0.250000	0.0209 (7)	
H14	0.500000	0.614152	0.249999	0.025*	
C17	0.6610 (2)	0.2555 (2)	0.1346 (3)	0.0209 (4)	
H17A	0.728384	0.210384	0.216707	0.031*	
H17B	0.703983	0.311872	0.087995	0.031*	
H17C	0.605857	0.195208	0.060762	0.031*	

### Trichlorido(2,6-dimethylpyridine-κN)gold(III) (7) Atomic displacement parameters (Å^2^)

**Table:**

	*U*^11^	*U*^22^	*U*^33^	*U*^12^	*U*^13^	*U*^23^
Au1	0.00944 (6)	0.00806 (5)	0.01165 (6)	0.000	0.00326 (4)	0.000
Cl1	0.0217 (4)	0.0089 (3)	0.0221 (4)	0.000	0.0047 (3)	0.000
Cl2	0.0149 (2)	0.0166 (2)	0.0153 (2)	0.00108 (17)	0.00006 (19)	−0.00011 (17)
N11	0.0128 (11)	0.0086 (10)	0.0119 (10)	0.000	0.0031 (9)	0.000
C12	0.0121 (9)	0.0150 (9)	0.0114 (9)	−0.0026 (7)	0.0028 (8)	−0.0004 (7)
C13	0.0205 (11)	0.0143 (9)	0.0171 (10)	−0.0062 (8)	0.0025 (9)	0.0015 (8)
C14	0.0222 (16)	0.0106 (13)	0.0226 (16)	0.000	0.0011 (13)	0.000
C17	0.0224 (11)	0.0214 (11)	0.0249 (11)	−0.0048 (9)	0.0157 (9)	−0.0040 (9)

### Trichlorido(2,6-dimethylpyridine-κN)gold(III) (7) Geometric parameters (Å, º)

**Table:**

Au1—N11	2.036 (2)	C12—C17	1.491 (3)
Au1—Cl1	2.2648 (7)	C13—C14	1.380 (3)
Au1—Cl2	2.2811 (5)	C13—H13	0.9500
Au1—Cl2^i^	2.2811 (5)	C14—H14	0.9500
N11—C12^i^	1.360 (2)	C17—H17A	0.9800
N11—C12	1.360 (2)	C17—H17B	0.9800
C12—C13	1.385 (3)	C17—H17C	0.9800
			
N11—Au1—Cl1	180.0	C14—C13—C12	119.8 (2)
N11—Au1—Cl2	89.375 (12)	C14—C13—H13	120.1
Cl1—Au1—Cl2	90.625 (12)	C12—C13—H13	120.1
N11—Au1—Cl2^i^	89.375 (12)	C13—C14—C13^i^	119.9 (3)
Cl1—Au1—Cl2^i^	90.625 (12)	C13—C14—H14	120.1
Cl2—Au1—Cl2^i^	178.75 (2)	C13^i^—C14—H14	120.1
C12^i^—N11—C12	121.7 (2)	C12—C17—H17A	109.5
C12^i^—N11—Au1	119.17 (12)	C12—C17—H17B	109.5
C12—N11—Au1	119.16 (12)	H17A—C17—H17B	109.5
N11—C12—C13	119.4 (2)	C12—C17—H17C	109.5
N11—C12—C17	118.50 (18)	H17A—C17—H17C	109.5
C13—C12—C17	122.0 (2)	H17B—C17—H17C	109.5
			
Cl2—Au1—N11—C12^i^	−92.76 (10)	C12^i^—N11—C12—C17	178.9 (2)
Cl2^i^—Au1—N11—C12^i^	87.24 (10)	Au1—N11—C12—C17	−1.1 (2)
Cl2—Au1—N11—C12	87.24 (10)	N11—C12—C13—C14	0.5 (3)
Cl2^i^—Au1—N11—C12	−92.76 (10)	C17—C12—C13—C14	−178.59 (17)
C12^i^—N11—C12—C13	−0.27 (15)	C12—C13—C14—C13^i^	−0.27 (15)
Au1—N11—C12—C13	179.73 (15)		

Symmetry code: (i) −*x*+1, *y*, −*z*+1/2.

### Trichlorido(2,6-dimethylpyridine-κN)gold(III) (7) Hydrogen-bond geometry (Å, º)

**Table:**

*D*—H···*A*	*D*—H	H···*A*	*D*···*A*	*D*—H···*A*
C17—H17*C*···Cl1^ii^	0.98	2.87	3.701 (2)	144
C14—H14···Cl1^iii^	0.95	2.67	3.621 (3)	180

Symmetry codes: (ii) −*x*+1, −*y*, −*z*; (iii) *x*, *y*+1, *z*.

### Tribromido(2-methylpyridine-κN)gold(III)–tribromido(3,5-dimethylpyridine-κN)gold(III) (8) Crystal data

**Table:**

[AuBr_3_(C_7_H_9_N)]·[AuBr_3_(C_6_H_7_N)]	*Z* = 2
*M**_r_* = 1073.67	*F*(000) = 952
Triclinic, *P*1	*D*_x_ = 3.290 Mg m^−^^3^
*a* = 9.1741 (9) Å	Mo *K*α radiation, λ = 0.71073 Å
*b* = 11.1922 (9) Å	Cell parameters from 9704 reflections
*c* = 11.4596 (7) Å	θ = 2.4–28.2°
α = 83.990 (6)°	µ = 24.58 mm^−^^1^
β = 80.777 (6)°	*T* = 100 K
γ = 69.147 (8)°	Plate, red
*V* = 1083.92 (16) Å^3^	0.13 × 0.06 × 0.02 mm

### Tribromido(2-methylpyridine-κN)gold(III)–tribromido(3,5-dimethylpyridine-κN)gold(III) (8) Data collection

**Table:**

Oxford Diffraction Xcalibur, Eos diffractometer	7479 independent reflections
Radiation source: Enhance (Mo) X-ray Source	5015 reflections with *I* > 2σ(*I*)
Graphite monochromator	*R*_int_ = –
Detector resolution: 16.1419 pixels mm^-1^	θ_max_ = 28.3°, θ_min_ = 2.4°
ω scans	*h* = −12→12
Absorption correction: multi-scan (CrysAlisPro; Rigaku OD, 2020)	*k* = −14→14
*T*_min_ = 0.052, *T*_max_ = 1.000	*l* = −15→15
7479 measured reflections	

### Tribromido(2-methylpyridine-κN)gold(III)–tribromido(3,5-dimethylpyridine-κN)gold(III) (8) Refinement

**Table:**

Refinement on *F*^2^	Primary atom site location: structure-invariant direct methods
Least-squares matrix: full	Secondary atom site location: difference Fourier map
*R*[*F*^2^ > 2σ(*F*^2^)] = 0.035	Hydrogen site location: inferred from neighbouring sites
*wR*(*F*^2^) = 0.055	H-atom parameters constrained
*S* = 0.82	*w* = 1/[σ^2^(*F*_o_^2^) + (0.0197*P*)^2^] where *P* = (*F*_o_^2^ + 2*F*_c_^2^)/3
7479 reflections	(Δ/σ)_max_ < 0.001
212 parameters	Δρ_max_ = 2.15 e Å^−^^3^
0 restraints	Δρ_min_ = −1.91 e Å^−^^3^

### Tribromido(2-methylpyridine-κN)gold(III)–tribromido(3,5-dimethylpyridine-κN)gold(III) (8) Fractional atomic coordinates and isotropic or equivalent isotropic displacement parameters (Å^2^)

**Table:**

	*x*	*y*	*z*	*U*_iso_*/*U*_eq_	
Au1	0.31055 (5)	0.18855 (4)	0.47765 (3)	0.01428 (9)	
Au2	0.26051 (4)	0.26264 (4)	0.06975 (3)	0.01319 (9)	
Br1	0.12467 (12)	0.08172 (11)	0.48592 (9)	0.0298 (3)	
Br2	0.48387 (11)	0.05181 (9)	0.32392 (8)	0.0193 (2)	
Br3	0.13402 (12)	0.33442 (10)	0.62169 (9)	0.0229 (2)	
Br4	0.44662 (11)	0.37133 (9)	0.03511 (8)	0.0208 (2)	
Br5	0.38703 (11)	0.14154 (9)	−0.10337 (8)	0.0235 (3)	
Br6	0.13174 (11)	0.38514 (9)	0.24300 (8)	0.0201 (2)	
N11	0.4748 (9)	0.2745 (7)	0.4755 (6)	0.0151 (19)	
C12	0.5284 (11)	0.3271 (8)	0.3743 (8)	0.019 (2)	
H12	0.486387	0.326809	0.303804	0.022*	
C13	0.6413 (10)	0.3808 (8)	0.3701 (7)	0.015 (2)	
C14	0.7044 (10)	0.3796 (8)	0.4740 (8)	0.017 (2)	
H14	0.784577	0.414986	0.473172	0.021*	
C15	0.6476 (11)	0.3256 (8)	0.5787 (8)	0.018 (2)	
C16	0.5329 (10)	0.2740 (8)	0.5777 (8)	0.017 (2)	
H16	0.493571	0.237639	0.648890	0.020*	
C17	0.6978 (11)	0.4381 (8)	0.2553 (7)	0.023 (2)	
H17A	0.672071	0.530073	0.262125	0.035*	
H17B	0.646182	0.424555	0.191795	0.035*	
H17C	0.812018	0.396959	0.236885	0.035*	
C18	0.7075 (11)	0.3281 (9)	0.6942 (8)	0.028 (3)	
H18A	0.661146	0.280832	0.756858	0.042*	
H18B	0.677659	0.417031	0.715550	0.042*	
H18C	0.822385	0.288205	0.684560	0.042*	
N21	0.1008 (9)	0.1688 (7)	0.1000 (6)	0.0181 (19)	
C22	−0.0324 (12)	0.2102 (9)	0.0472 (8)	0.024 (3)	
C23	−0.1378 (12)	0.1462 (10)	0.0739 (9)	0.027 (3)	
H23	−0.229091	0.172514	0.035102	0.032*	
C24	−0.1124 (11)	0.0427 (10)	0.1576 (9)	0.027 (3)	
H24	−0.186489	0.000153	0.178792	0.032*	
C25	0.0248 (12)	0.0055 (9)	0.2077 (8)	0.028 (3)	
H25	0.046356	−0.064793	0.264405	0.033*	
C26	0.1292 (12)	0.0668 (9)	0.1779 (7)	0.024 (2)	
H26	0.224149	0.037801	0.212418	0.028*	
C27	−0.0574 (11)	0.3212 (9)	−0.0393 (8)	0.025 (3)	
H27A	−0.054252	0.394707	−0.001573	0.037*	
H27B	−0.160173	0.342710	−0.066478	0.037*	
H27C	0.025593	0.299627	−0.107068	0.037*	

### Tribromido(2-methylpyridine-κN)gold(III)–tribromido(3,5-dimethylpyridine-κN)gold(III) (8) Atomic displacement parameters (Å^2^)

**Table:**

	*U*^11^	*U*^22^	*U*^33^	*U*^12^	*U*^13^	*U*^23^
Au1	0.0161 (2)	0.0168 (2)	0.01202 (19)	−0.00802 (17)	−0.00045 (15)	−0.00383 (15)
Au2	0.0150 (2)	0.0127 (2)	0.01178 (19)	−0.00456 (17)	−0.00136 (15)	−0.00158 (15)
Br1	0.0368 (7)	0.0457 (8)	0.0205 (6)	−0.0322 (6)	0.0043 (4)	−0.0086 (5)
Br2	0.0224 (5)	0.0190 (6)	0.0171 (5)	−0.0080 (4)	0.0021 (4)	−0.0074 (4)
Br3	0.0177 (5)	0.0311 (6)	0.0204 (5)	−0.0085 (5)	0.0045 (4)	−0.0138 (4)
Br4	0.0217 (6)	0.0206 (6)	0.0231 (5)	−0.0116 (5)	−0.0017 (4)	−0.0008 (4)
Br5	0.0271 (6)	0.0279 (6)	0.0173 (5)	−0.0121 (5)	0.0043 (4)	−0.0100 (5)
Br6	0.0213 (5)	0.0192 (5)	0.0189 (5)	−0.0057 (4)	0.0006 (4)	−0.0068 (4)
N11	0.012 (4)	0.014 (4)	0.021 (4)	−0.005 (4)	−0.001 (4)	−0.009 (3)
C12	0.018 (5)	0.012 (5)	0.019 (5)	0.002 (4)	−0.001 (4)	−0.007 (4)
C13	0.013 (5)	0.010 (5)	0.018 (5)	0.000 (4)	0.002 (4)	−0.007 (4)
C14	0.013 (5)	0.016 (5)	0.024 (5)	−0.004 (4)	−0.001 (4)	−0.008 (4)
C15	0.014 (5)	0.015 (5)	0.022 (5)	−0.001 (4)	−0.007 (4)	0.001 (4)
C16	0.018 (5)	0.018 (5)	0.013 (5)	−0.006 (4)	−0.003 (4)	0.000 (4)
C17	0.033 (6)	0.021 (6)	0.022 (5)	−0.015 (5)	−0.001 (4)	−0.010 (4)
C18	0.021 (6)	0.034 (6)	0.036 (6)	−0.016 (5)	−0.017 (5)	0.013 (5)
N21	0.019 (5)	0.017 (5)	0.013 (4)	−0.001 (4)	0.002 (3)	−0.003 (3)
C22	0.024 (6)	0.023 (6)	0.018 (5)	0.005 (5)	−0.006 (5)	−0.013 (5)
C23	0.014 (5)	0.035 (7)	0.031 (6)	−0.006 (5)	0.004 (5)	−0.019 (5)
C24	0.018 (6)	0.030 (7)	0.035 (6)	−0.012 (5)	0.013 (5)	−0.025 (5)
C25	0.036 (7)	0.023 (6)	0.026 (6)	−0.015 (5)	0.003 (5)	−0.002 (5)
C26	0.032 (6)	0.029 (6)	0.008 (5)	−0.008 (5)	−0.003 (4)	−0.001 (4)
C27	0.023 (6)	0.029 (6)	0.019 (5)	−0.002 (5)	−0.007 (4)	−0.003 (4)

### Tribromido(2-methylpyridine-κN)gold(III)–tribromido(3,5-dimethylpyridine-κN)gold(III) (8) Geometric parameters (Å, º)

**Table:**

Au1—N11	2.053 (8)	C17—H17B	0.9800
Au1—Br1	2.3922 (11)	C17—H17C	0.9800
Au1—Br3	2.4162 (9)	C18—H18A	0.9800
Au1—Br2	2.4306 (10)	C18—H18B	0.9800
Au2—N21	2.056 (8)	C18—H18C	0.9800
Au2—Br4	2.3901 (11)	N21—C26	1.348 (11)
Au2—Br5	2.4136 (9)	N21—C22	1.360 (11)
Au2—Br6	2.4253 (10)	C22—C23	1.376 (14)
N11—C12	1.347 (11)	C22—C27	1.480 (12)
N11—C16	1.360 (10)	C23—C24	1.400 (13)
C12—C13	1.362 (12)	C23—H23	0.9500
C12—H12	0.9500	C24—C25	1.377 (13)
C13—C14	1.402 (11)	C24—H24	0.9500
C13—C17	1.501 (12)	C25—C26	1.349 (13)
C14—C15	1.395 (12)	C25—H25	0.9500
C14—H14	0.9500	C26—H26	0.9500
C15—C16	1.371 (12)	C27—H27A	0.9800
C15—C18	1.519 (11)	C27—H27B	0.9800
C16—H16	0.9500	C27—H27C	0.9800
C17—H17A	0.9800		
			
N11—Au1—Br1	177.6 (2)	C13—C17—H17C	109.5
N11—Au1—Br3	90.3 (2)	H17A—C17—H17C	109.5
Br1—Au1—Br3	90.02 (4)	H17B—C17—H17C	109.5
N11—Au1—Br2	89.0 (2)	C15—C18—H18A	109.5
Br1—Au1—Br2	90.84 (4)	C15—C18—H18B	109.5
Br3—Au1—Br2	176.66 (4)	H18A—C18—H18B	109.5
N21—Au2—Br4	179.8 (2)	C15—C18—H18C	109.5
N21—Au2—Br5	89.90 (19)	H18A—C18—H18C	109.5
Br4—Au2—Br5	90.14 (4)	H18B—C18—H18C	109.5
N21—Au2—Br6	89.95 (19)	C26—N21—C22	120.6 (9)
Br4—Au2—Br6	90.01 (4)	C26—N21—Au2	117.7 (7)
Br5—Au2—Br6	179.59 (4)	C22—N21—Au2	121.6 (7)
C12—N11—C16	120.2 (9)	N21—C22—C23	119.1 (9)
C12—N11—Au1	120.9 (6)	N21—C22—C27	118.7 (10)
C16—N11—Au1	118.8 (6)	C23—C22—C27	122.2 (9)
N11—C12—C13	121.9 (9)	C22—C23—C24	120.9 (9)
N11—C12—H12	119.1	C22—C23—H23	119.5
C13—C12—H12	119.1	C24—C23—H23	119.5
C12—C13—C14	118.9 (9)	C25—C24—C23	117.1 (10)
C12—C13—C17	120.0 (8)	C25—C24—H24	121.5
C14—C13—C17	121.1 (9)	C23—C24—H24	121.5
C15—C14—C13	119.0 (9)	C26—C25—C24	121.2 (10)
C15—C14—H14	120.5	C26—C25—H25	119.4
C13—C14—H14	120.5	C24—C25—H25	119.4
C16—C15—C14	119.6 (8)	N21—C26—C25	121.0 (9)
C16—C15—C18	119.9 (9)	N21—C26—H26	119.5
C14—C15—C18	120.5 (9)	C25—C26—H26	119.5
N11—C16—C15	120.5 (9)	C22—C27—H27A	109.5
N11—C16—H16	119.7	C22—C27—H27B	109.5
C15—C16—H16	119.7	H27A—C27—H27B	109.5
C13—C17—H17A	109.5	C22—C27—H27C	109.5
C13—C17—H17B	109.5	H27A—C27—H27C	109.5
H17A—C17—H17B	109.5	H27B—C27—H27C	109.5
			
Br3—Au1—N11—C12	−121.6 (6)	Br5—Au2—N21—C26	96.6 (6)
Br2—Au1—N11—C12	55.2 (6)	Br6—Au2—N21—C26	−83.8 (6)
Br3—Au1—N11—C16	60.5 (6)	Br5—Au2—N21—C22	−86.0 (7)
Br2—Au1—N11—C16	−122.7 (6)	Br6—Au2—N21—C22	93.7 (7)
C16—N11—C12—C13	0.1 (12)	C26—N21—C22—C23	−0.4 (14)
Au1—N11—C12—C13	−177.7 (6)	Au2—N21—C22—C23	−177.7 (6)
N11—C12—C13—C14	0.8 (13)	C26—N21—C22—C27	−179.0 (8)
N11—C12—C13—C17	179.9 (7)	Au2—N21—C22—C27	3.6 (12)
C12—C13—C14—C15	−1.1 (12)	N21—C22—C23—C24	2.4 (14)
C17—C13—C14—C15	179.8 (8)	C27—C22—C23—C24	−178.9 (9)
C13—C14—C15—C16	0.6 (13)	C22—C23—C24—C25	−2.5 (14)
C13—C14—C15—C18	−177.1 (8)	C23—C24—C25—C26	0.5 (15)
C12—N11—C16—C15	−0.7 (12)	C22—N21—C26—C25	−1.6 (14)
Au1—N11—C16—C15	177.2 (6)	Au2—N21—C26—C25	175.8 (7)
C14—C15—C16—N11	0.3 (13)	C24—C25—C26—N21	1.6 (15)
C18—C15—C16—N11	178.1 (8)		

### Tribromido(2-methylpyridine-κN)gold(III)–tribromido(3,5-dimethylpyridine-κN)gold(III) (8) Hydrogen-bond geometry (Å, º)

**Table:**

*D*—H···*A*	*D*—H	H···*A*	*D*···*A*	*D*—H···*A*
C18—H18*C*···Br3^i^	0.98	3.05	3.892 (10)	145
C23—H23···Br4^ii^	0.95	3.01	3.815 (10)	143
C17—H17*C*···Br6^i^	0.98	2.90	3.792 (10)	152
C12—H12···Br4	0.95	3.13	4.023 (9)	158
C16—H16···Br5^iii^	0.95	3.06	4.007 (9)	172
C25—H25···Br1^iv^	0.95	3.06	3.721 (10)	128
C26—H26···Br2	0.95	2.94	3.835 (10)	159
C27—H27*C*···Br3^v^	0.98	3.14	4.010 (9)	149

Symmetry codes: (i) *x*+1, *y*, *z*; (ii) *x*−1, *y*, *z*; (iii) *x*, *y*, *z*+1; (iv) −*x*, −*y*, −*z*+1; (v) *x*, *y*, *z*−1.
